# Structure-Guided Identification of Resistance Breaking Antimalarial *N*‑Myristoyltransferase Inhibitors

**DOI:** 10.1016/j.chembiol.2019.03.015

**Published:** 2019-07-18

**Authors:** Anja C. Schlott, Stephen Mayclin, Alexandra R. Reers, Olivia Coburn-Flynn, Andrew S. Bell, Judith Green, Ellen Knuepfer, David Charter, Roger Bonnert, Brice Campo, Jeremy Burrows, Sally Lyons-Abbott, Bart L. Staker, Chun-Wa Chung, Peter J. Myler, David A. Fidock, Edward W. Tate, Anthony A. Holder

**Affiliations:** 1 Francis Crick Institute, 1 Midland Road, London NW1 1AT, UK; 2 Molecular Sciences Research Hub, Imperial College, White City Campus Wood Lane, London W12 0BZ, UK; 3 Seattle Structural Genomics Center for Infectious Disease (SSGCID), Seattle, WA 98109, USA; 4 UCB Pharma, 7869 NE Day Road West, Bainbridge Island, WA 98110, USA; 5 Center for Global Infectious Disease Research, Seattle Children's Research Institute, 307 Westlake Avenue North, Suite 500, Seattle, USA; 6 Department of Microbiology & Immunology, Columbia University Medical Center, New York, NY 10032, USA; 7 Structural and Biophysical Sciences, GlaxoSmithKline, Stevenage, Hertfordshire, UK; 8 Medicines for Malaria Venture, Route de Pré-Bois 20, Post Box 1826, 1215 Geneva 15, Switzerland; 9 Crick–GSK Biomedical LinkLabs, GSK Medicines Research Centre, Stevenage, UK; 10 Department of Biomedical Informatics & Medical Education, University of Washington, Seattle, USA; 11 Department of Global Health, University of Washington, Seattle, USA; 12 Division of Infectious Diseases, Department of Medicine, Columbia University Medical Center, New York, NY 10032, USA

**Keywords:** antimalarial target, crystal structure, drug resistance development, genetic manipulation, malaria, myristoylation, N-myristoyltransferase, *Plasmodium*, post-translational modification, protein lipidation

## Abstract

The attachment of myristate to the N-terminal glycine of certain proteins is largely a co-translational modification catalyzed by N-myristoyltransferase (NMT), and involved in protein membrane-localization. Pathogen NMT is a validated therapeutic target in numerous infectious diseases including malaria. In *Plasmodium falciparum*, NMT substrates are important in essential processes including parasite gliding motility and host cell invasion. Here, we generated parasites resistant to a particular NMT inhibitor series and show that resistance in an *in vitro* parasite growth assay is mediated by a single amino acid substitution in the NMT substrate-binding pocket. The basis of resistance was validated and analyzed with a structure-guided approach using crystallography, in combination with enzyme activity, stability, and surface plasmon resonance assays, allowing identification of another inhibitor series unaffected by this substitution. We suggest that resistance studies incorporated early in the drug development process help selection of drug combinations to impede rapid evolution of parasite resistance.

## Highlights

• Discovery of a mutant offering resistance against *P. falciparum* NMT inhibitor IMP-1002
• Structural basis of the mechanism of resistance revealed by X-ray crystallography
• Genetic and chemical validation of resistance using CRISPR-Cas9 and enzyme assays
• Crystal structures of PvNMT[G386E] with IMP-1002 versus DDD85646, overcoming resistance

## Graphical Abstract

**Figure:**
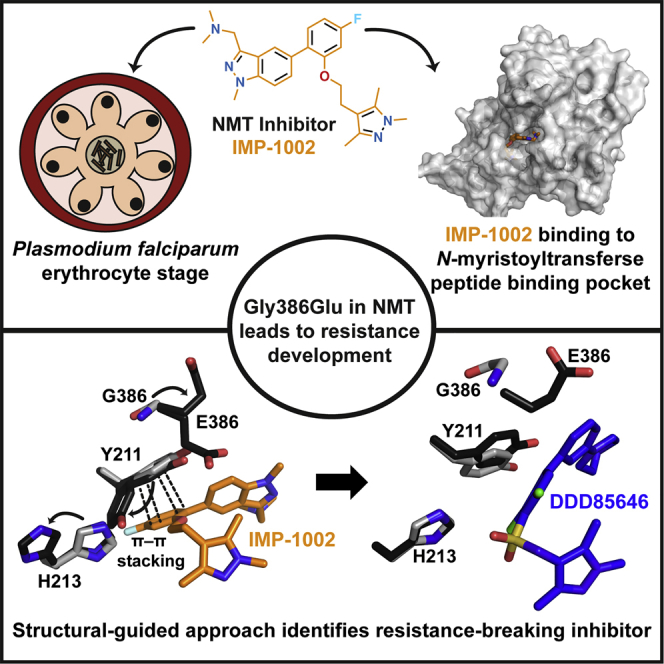

## Teaser

Structural studies of N-myristoyltransferase (NMT) of the malaria parasite *Plasmodium falciparum* combined with inhibitors decipher how a point mutation in *nmt* leads to the development of resistance against an inhibitor series, and enables identification of an NMT inhibitor that can overcome this resistance phenotype.

## Introduction

Malaria, caused by parasitic protozoa of the genus *Plasmodium*, led to an estimated 216 million clinical cases and nearly half a million deaths in 2016 (World Health Organization, 2017). In humans, malaria is caused by five species of the genus *Plasmodium* (Sutherland et al., 2010), of which *P. falciparum* is the most important. The escalating incidence of resistance to front-line drugs including artemisinin-based combination therapies threatens global efforts to control and eliminate malaria and highlights the urgent need to identify new chemotherapeutic strategies. In an experimental research setting, selection of drug resistance can be exploited to identify and validate drug targets, understand the molecular mechanisms underlying decreased parasite killing, guide targeted drug discovery efforts, and to improve inhibitor binding, specificity and selectivity (Ariey et al., 2014, Cowell et al., 2018, Flannery et al., 2013).

The enzyme NMT, which acylates the N-terminal glycine of substrate proteins, is a promising target for antimalarial drug development (Wright et al., 2014). This enzyme is important for the survival and viability of a wide range of parasites (*Plasmodium*, *Leishmania*, and *Trypanosoma* species) and fungi (Bell et al., 2012, Brannigan et al., 2010, Fang et al., 2015, Frearson et al., 2010, Goncalves et al., 2012b, Hutton et al., 2014, Mousnier et al., 2018, Olaleye et al., 2014, Rackham et al., 2014, Schlott et al., 2018, Tate et al., 2013, Wright et al., 2014, Wright et al., 2016, Wright et al., 2015, Yu et al., 2012), which each encode a single *nmt* gene (PF3D7_1412800). Using coenzyme A (CoA)-activated lipid, NMT transfers myristate to the substrate protein, after removal of the initiator methionine, in a predominantly co-translational process (Ritzefeld et al., 2017, Schlott et al., 2018). Given the importance of myristoylation for several vital processes in *P. falciparum* (Wright et al., 2014), the development of selective and potent small-molecule NMT inhibitors (NMTi) could form the basis of new medicines against malaria. The design of inhibitors against both *P. falciparum* (Pf) and *P. vivax* (Pv) NMTs has focused on generating potent compounds selective over the two human (Hs) NMTs known as HsNMT1 and HsNMT2, targeting the peptide-binding pocket, and optimizing the pharmacokinetic profile. Recent developments include exploitation of inhibitor scaffolds identified in two large high-throughput screens (Bell et al., 2012, Goncalves et al., 2012b, Mousnier et al., 2018). These compounds include a quinoline series (Goncalves et al., 2012b) and four additional series used to address the issue of selectivity over host NMTs. Unfortunately, several of these chemical starting points lacked cell-based activity, potentially because of poor cellular uptake (Bell et al., 2012), as reviewed by Ritzefeld et al. (2017). A further series was explored against *Leishmania donovani* (Ld) NMT as well as PfNMT, showing improved selectivity over HsNMT1 and HsNMT2 and with promising activity against liver-stage parasites (Rackham et al., 2014, Rackham et al., 2015, Yu et al., 2015).

Here we report discovery of an enzyme variant, PfNMT[G386E] that gives rise to parasite resistance to NMTi IMP-1002, and demonstrate its sufficiency for resistance using CRISPR-Cas9-based genome editing. The effect of this amino acid substitution on enzyme function and inhibition is explored biochemically and in parasites using a range of NMTi series. This allowed selection of compounds to solve the X-ray structure for the corresponding PvNMT wild type (WT) and variant in complex with inhibitor. Structure-guided analysis of these data enabled discovery of an inhibitor series that can overcome parasite resistance to NMTi. Collectively, our studies identify a mechanism of NMTi resistance early in the drug-development process, and provide a strategy to overcome parasite resistance to NMTi.

## Results

### A Single Amino Acid Substitution in the *P. falciparum* N-Myristoyltransferase Active Site Leads to Parasite Resistance to the NMT Inhibitor IMP-1002

**Figure 1:**
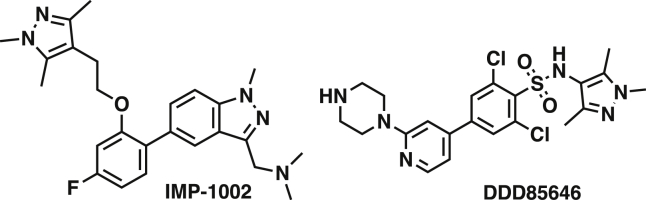
Chemical Structures of NMT Inhibitors IMP-1002 and DDD86646

We first sought to generate a *P. falciparum* mutant line resistant to NMTi IMP-1002 (Figure 1). IMP-1002 is a previously undisclosed member of our recently reported series of human NMT inhibitors (Mousnier et al., 2018), and a close analog of IMP-0917 (Figure S1) that was discovered through a fragment reconstruction approach based on hits from screens against PvNMT and PfNMT. With parasite growth half maximal effective concentration (EC_50_) inhibitory activity of 34 nM (Figure S2A), IMP-1002 is 4-fold more potent in killing parasites than the most potent previously reported PfNMT inhibitor DDD85646 (Figure S2A) (Wright et al., 2014). We have shown previously that *P. falciparum* 3D7 parasites normally progress to schizont forms that contain 20–30 nuclei at the end of the 48-h intracellular development in the red blood cell. Treatment at the beginning of this cycle with four times the EC_50_ concentration of DDD85646 caused the parasite to develop abnormally and stall at a morphologically distinct “pseudoschizont” stage with 4–6 nuclei (Wright et al., 2014) (Figure S2B). Furthermore, NMTi blocked formation of the parasite inner membrane complex (IMC), a critical subcellular compartment comprising a set of flattened vesicular sacs, which form part of the parasite's surface pellicle essential for red blood cell invasion. Consistent with a conserved mode of action, at four times the EC_50_ value IMP-1002 phenocopied this stage-specific block in 3D7 parasite development, preventing parasite growth beyond the four- to five-nuclei stage and inhibiting accumulation of the NMT substrate glideosome-associated protein 45 (GAP45). This protein bridges the parasite plasma membrane and the IMC. GAP45 is anchored at the plasma membrane through myristoylation and palmitoylation of its N terminus and at the IMC through palmitoylation of its C terminus, and serves as a marker for correct IMC formation and recruitment of other proteins, such as MyoA and MTIP, to form the glideosome involved in invasion (Perrin et al., 2018) (Figures S2B and S2C).

**Figure 2:**
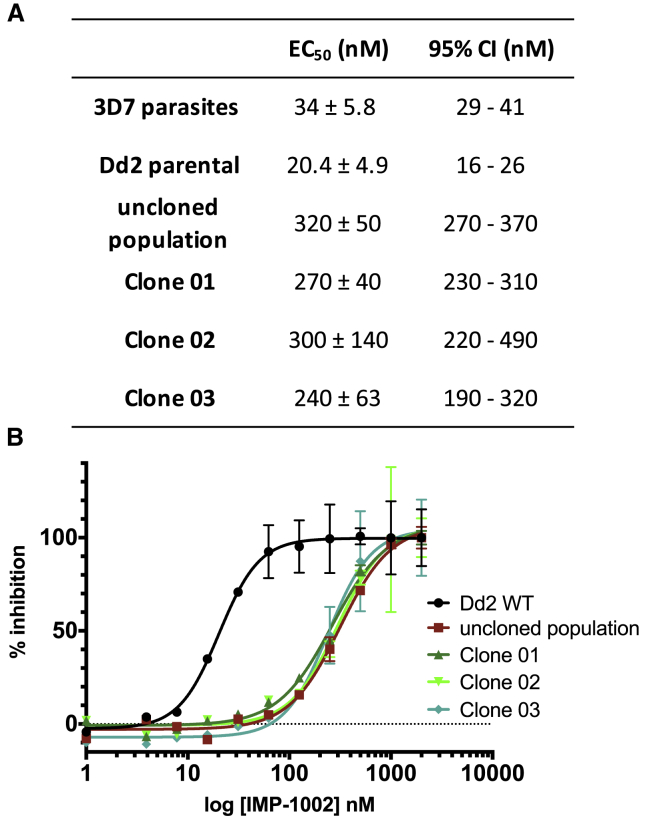
IMP-1002 Inhibition of Parental and Resistant Parasite Lines (A) Inhibition of *P. falciparum* 3D7 and Dd2 parental parasites used to raise the resistant clones 01, 02, 03, and the uncloned population, showing the respective EC_50_ and confidence interval values rounded to two significant figures. (B) Measurement of the inhibition of growth of the Dd2 parental line, the resistant uncloned population and three IMP-1002-resistant clones to determine the EC_50_ after 72 h (n = 3). There is a clear shift of the EC_50_ for the resistant bulk population and the three parasite clones. Error bars indicate standard deviation of the mean.

The *P. falciparum* Dd2-B2 parasite line (recloned from the Dd2 parental line by limiting dilution) was used to select for resistant parasites (Flannery et al., 2013). IMP-1002 EC_50_ for Dd2 parasites was comparable with that of the 3D7 line (Figure 2A). After 36 days in the presence of 120 nM IMP-1002 (3.4 times EC_50_ in this parasite line) parasite growth was detected in one culture with an initial 10^6^ parasite inoculum. Resistance to IMP-1002 was confirmed in the bulk population and three parasite clones (Figure 2B). Both IMP-1002-resistant clones showed a 14-fold increase in IMP-1002 EC_50_ values (Figure 2B). To discover the genetic basis for this shift in EC_50_ values, the *nmt* gene of two parasite clones as well as the resistant Dd2 bulk population was sequenced and compared with that of the Dd2 parental line. The same single non-synonymous point mutation (G1544A), leading to an amino acid substitution from glycine to glutamic acid (G386E) in the NMT protein, was present in the bulk population and each individual clone.

### CRISPR-Cas Gene Editing of *P. falciparum* 3D7 Demonstrates that PfNMT[G386E] Is Sufficient to Mediate Resistance to IMP-1002

**Figure 3:**
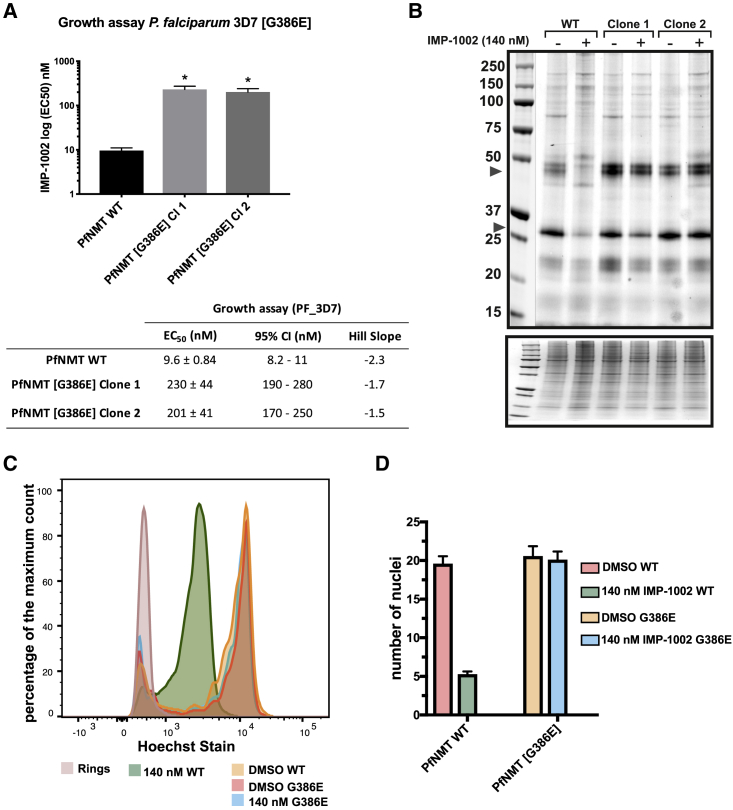
NMTi IMP-1002 Shows On-Target Specificity (A) Growth assay with *P. falciparum* 3D7[G386E] clones and [WT] showing shift in EC_50_ for both clones from transfections with independent guides (n = 3). Unpaired Welch t test not assuming equal standard deviations, p = 0.01. (B) NMT substrates labeled with YnMyr in WT and G386E parasites after treatment with NaOH to hydrolyze the base-labile ester-linkage incorporation of YnMyr into GPI-anchored proteins. Although WT parasites show a decreased band intensity in response to the NMTi treatment, G386E parasite proteins remain unchanged compared with those of the DMSO control. The Coomassie blue-stained gel shows proportional loading of total protein for each sample. (C) Percentage of maximum count of Hoechst-stained WT and G386E parasites used to determine the number of nuclei per sample. (D) *P. falciparum* [G386E] parasites show 0% inhibition at 140 nM. Graph shows flow cytometry data of Hoechst-stained parasites in (C) treated with 140 nM IMP-1002 from 1 to 45 h post-invasion. The median fluorescent intensity (MFI) of the Hoechst-positive parasites was normalized to the MFI of parasites containing one nucleus (ring sample) in (C) to determine the average number of nuclei per sample. While WT parasites show a drop-in the number of nuclei and therefore an arrest in development at 140 nM of NMTi, G386E parasites show no arrest in development. All errors bars in this figure indicate standard deviation of the mean.

To confirm the sufficiency of the G386E substitution for IMP-1002 resistance, we introduced the mutation into the *nmt* gene in 3D7 parasites. Genetic modification of *P. falciparum* 3D7 (Figure S3) was performed using a CRISPR-Cas9 approach (Ghorbal et al., 2014, Wagner et al., 2014, Zhang et al., 2014) as described by Knuepfer et al. (2017), using two independent guide RNA sequences to target Cas9 nuclease to the *nmt* locus. Parasites were visible 17 days post-transfection, and were subsequently screened for DNA integration, treated with 5-fluorocytosine, and cloned by limiting dilution using the plaque assay to confirm single parasites per well (Thomas et al., 2016). Integration of the single nucleotide change leading to the G386E substitution was confirmed by sequence analysis following PCR amplification of genomic DNA. The SYBR Green-based growth assay revealed a 22-fold increase in IMP-1002 EC_50_ over the parental 3D7 line for the two tested clones from independent transfections (Figure 3A). These data confirm that this amino acid substitution is responsible for resistance to IMP-1002.

Parasites expressing PfNMT[G386E] had been selected by growth *in vitro* in the presence of IMP-1002, and it was possible that there would be a fitness cost associated with this amino acid change in absence of inhibitor, for example, by interference with the binding of one or more of the large number of substrates that are myristoylated. Therefore, we searched for any resultant phenotype at the level of protein myristoylation and long-term growth rate. To investigate potential differences in substrate engagement and myristoylation pattern, we biosynthetically labeled both WT parasites and two PfNMT[G386E] parasite clones with the myristic acid analog YnMyr throughout the complete intraerythrocytic cycle (Wright et al., 2014). There was very little difference in labeling pattern, and therefore no detectable effect of the G386E substitution on global myristoylation (Figure S4A), while continuous culture of the two PfNMT[G386E] clones revealed no significant growth defect over six generations as assessed by relative parasitemia, normalized to the WT population (Figure S4B). These data provide evidence that enzyme function is not compromised in the G386E variant.

### PfNMT[G386E] Parasites Resist Pharmacological Inhibition of Myristoylation and Parasite Development, Confirming On-Target Specificity of IMP-1002

To address the on-target specificity of IMP-1002 we investigated whether PfNMT[G386E] parasites also resist inhibition of N-myristoylation. WT and PfNMT[G386E] parasite clones were labeled with YnMyr in the presence or absence of 140 nM IMP-1002 (approximately 4-fold EC_50_ for WT 3D7 parasites) during the period 36–45 h post-invasion, when myristoylation is at its peak (Wright et al., 2014). Although there was a clear decrease in YnMyr labeling in WT parasites treated with IMP-1002, there was no reduction in labeling of the PfNMT[G386E] parasite clones with the inhibitor present (Figure 3B).

To determine whether PfNMT[G386E] parasite clones develop normally in the presence of 140 nM IMP1002, synchronized ring stages from WT parasites and one PfNMT[G386E] clone were treated with either 140 nM IMP-1002 or control DMSO for a period of 45 h. We then determined the number of nuclei within the cells by flow cytometry (Figure 3C). In the presence of IMP-1002, the WT parasite population contained a reduced number of nuclei compared with the DMSO control, while PfNMT[G386E] parasites showed no difference between IMP-1002-treated parasites at 140 nM and DMSO-treated control parasites (Figures 3C and 3D).

### PvNMT[G386E] Is Refractory to Binding and Inhibition by IMP-1002

**Figure 4:**
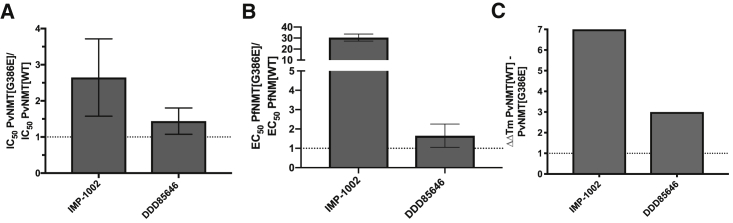
Consequences of G386E Substitution for NMT Function Bar graphs showing the effect of the G386E substitution on (A) IC_50_ in the biochemical assay, (B) EC_50_ in the parasite growth assay, and (C) thermal shift in a protein stability assay with IMP-1002 (n = 2–3). For comparison, the effect of the substitution on these values is shown for another NMT inhibitor, DDD85646, which is not affected. All errors bars in this figure indicate standard deviation of the mean. For Figure 4C refer to the Tables S4A and S4B.

We next turned our attention to the biochemical basis for resistance to IMP-1002 *in vitro* using *P. vivax* NMT (PvNMT) as a tractable model for PfNMT. PvNMT possesses 80% sequence identity and 93% similarity to PfNMT and is an excellent platform for structural studies, while to date PfNMT has proven intractable for X-ray crystallography. Activity measurements with PvNMT[WT] and PvNMT[G386E] enzymes used MyrCoA and a 15-residue NMT substrate peptide. The enzymes were of similar purity (Figure S5B) and had similar kinetic profiles over 45 min in a continuous assay (Figure S5A). Reactions used a coumarin derivative containing a thiol-reactive maleimide called 7-diethylamino-3-(4-maleimidophenyl)-4-methylcoumarin (CPM), to monitor the formation of the CoA thiol product (Goncalves et al., 2012a). A quenched assay after 30 min was used to determine IMP-1002 half maximal inhibitory concentration (IC_50_) for PvNMT[G386E] compared with PvNMT[WT], which indicated a shift to higher concentration for the variant enzyme visualized through a ratio of G386E/WT of 2.6 on average (Figure 4A). In comparison, the ratio of G386E/WT EC_50_ values in the parasite growth assay, was approximately 20-fold (Figures 3A and 4B). With the high potency of IMP-1002, the CPM assay is at its detection limit resulting in a reduced shift in the variant enzyme. To complement the CPM assay we performed a direct binding thermal shift assay with PvNMT[WT] and PvNMT[G386E]. Consistent with the high affinity of IMP-1002 for the PvNMT[WT] this protein was stabilized by >8.8°C with 5 μM of IMP-1002 even in the absence of MyrCoA. In contrast, the thermal stabilization was only 1.8°C with PvNMT[G386E], leading to a ΔΔT_m_ of >7.0°C between the two enzymes (Figure 4C; Table S4). The same assay was conducted in the presence of 4 μM MyrCoA, which showed a similar trend; however, because of the higher baseline melting temperature for the MyrCoA-bound proteins the stabilization window was compressed (Table S4B).

### X-Ray Crystallography Identifies the Structural Basis for IMP-1002 Resistance in PvNMT[G386E]

**Figure 5:**
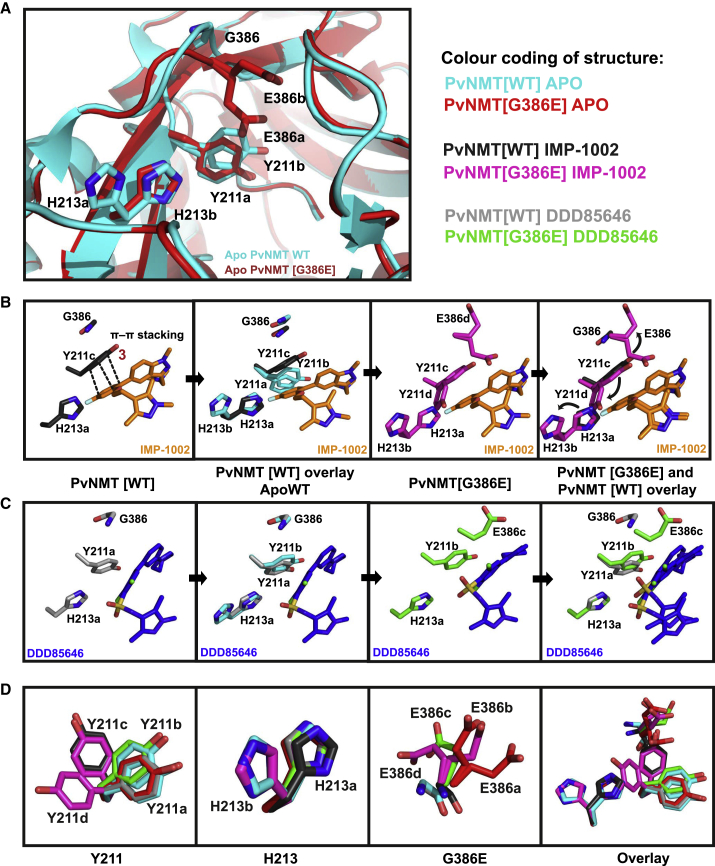
Crystallographic Analysis of NMT Inhibitor Interaction with PvNMT[WT] and PvNMT[G386E] (A) Crystal structure of PvNMT[WT] active site without inhibitor bound, overlaid with PvNMT[G386E]. (B) Movement of residues in the active site of PvNMT[G386E] compared with PvNMT[WT] on interaction with IMP-1002. (C and D) (C) Movement of residues in the active site of PvNMT[G386E] compared with PvNMT[WT] on DDD85646 binding (D) Y211 movement in the active site in the six different crystal structures. Y211 can adopt four distinct rotamers depending on the G386E mutation and type of inhibitor bound. This is followed by H213 that can adopt two different rotamer occupancies to accommodate the extra space taken up by Y211 when G386 is mutated to E386.

Having demonstrated that the G386E substitution in NMT was responsible for parasite resistance to NMTi, we next focused on understanding the structural basis for resistance. G386 does not directly contact the inhibitor in previously reported NMT co-crystal structures (Bell et al., 2012, Mousnier et al., 2018, Wright et al., 2014) leading us to consider a hypothesis based on indirect conformational changes in the inhibitor binding site. In contrast to G386, E386 places a large, charged side chain in direct proximity to the nearby Y211 residue. From comparisons of PvNMT and HsNMT1, Y211 is known to adopt at least two different preferred rotamers, with HsNMT showing a strong preference for one of these conformations; compounds which disfavor this preferred rotamer can achieve a high degree of selectivity for parasite NMT over HsNMT (Brannigan et al., 2014, Yu et al., 2012). The binding mode of IMP-1002 suggests that it packs tightly against Y211 (Figure 5B) and shows good selectivity over HsNMT, but this binding mode leaves little space for a glutamate side chain at position 386.

To test this hypothesis experimentally, recombinant PvNMT[G386E] was crystallized in the presence of MyrCoA ± IMP-1002 (PDB: 6MB0) (Table S1). There is minimal difference in the position of Y211 between the crystal structure of PvNMT[G386E] with bound MyrCoA (but no inhibitor) (PDB: 6MAY) and that of PvNMT[WT] (PDB: 2YNC) (Wright et al., 2014) (Figure 5A), with the tyrosine adopting one of two observed rotamers (designated “rotamer Y211a”) in the PvNMT[G386E] protein crystal and both rotamers Y211a and Y211b in the WT, indicating a degree of flexibility. Interestingly, the nearby His213 displays a larger degree of flexibility in the PvNMT[WT] structure, modeled as two possible rotamers (H213a, H213b), whereas the residue adopts a single conformation in the G386E variant (H213a). On IMP-1002 interaction with PvNMT[WT] (PDB: 6MB1), Y211 moves to a previously unobserved third rotamer conformation (Y211c) to form a π-π stacking interaction with the pendant phenyl ring of IMP-1002, while H213 remains in the unliganded position H213a (Figure 5B). On IMP-1002 binding to PvNMT[G386E], the glutamate side chain prevents formation of the more favorable π-π stacking interaction with the pendant phenyl ring, and forces H213 to move from rotamer position H213a to the more constrained H213b (Figure 5B). Both Y211 and H213 thus adopt alternative rotamers from those seen in PvNMT[WT] to create space for the inhibitor, leading to the loss of beneficial π-π stacking interactions consistent with the observed weaker affinity of IMP-1002 for the G386E variant (Figure 5B).

### Predictive Structure-Guided Identification of NMT Inhibitor that Overcomes G386E Resistance Both Biochemically and in the Parasite

Based on these structural insights, we explored NMTi binding modes that might deliver resistance-breaking activity. Examination of our previously reported structure of NMTi DDD85646 in complex with PvNMT[WT] (PDB: 2YND) (Wright et al., 2014) revealed a binding mode distinct from that of IMP-1002, which largely avoids direct interactions with Y211 (Figure 5C). Therefore, we measured the IC_50_ of DDD85646 with PvNMT[WT] and [G386E] enzyme and its EC_50_ with Pf3D7 [WT] and [G386E] parasite clones; compared with IMP-1002, the ratio of G386E/WT for DDD85646 was between 1 and 1.7 in both assays (CPM assay, IC_50_ ratio = 1.4 ± 0.35; growth assay, EC_50_ ratio = 1 ± 0.1), strengthening the hypothesis that DDD85646 binding is unaffected by G386E (Figures 4A and 4B; Tables S2 and S3). This was supported by thermal shift data with DDD85646 that confirmed the difference in stabilization (ΔΔT_m_) between PvNMT[WT] and PvNMT[G386E] was only 3°C, significantly less than the >7.0°C in favor of WT observed for IMP-1002 (Table S4A).

Structural studies supported this concept. We solved the structure of PvNMT[G386E] with bound DDD85646 (PDB: 6MAZ) (Table S1), and discovered that there is minimal movement of either Y211 or H213 in the inhibitor-bound form compared with PvNMT[WT], confirming the molecular basis for similar affinity (Figure 5C). Analysis of the PvNMT[G386E] structure with either IMP-1002 or DDD85646 bound demonstrated that the positions of all three amino acids (E386, Y211, and H213) were influenced by the binding mode of the inhibitor (Figure 5D). By including the PvNMT[WT] structure in the comparison, Y211 was observed to adopt at least four different conformations. A preferred conformation (rotamer Y211a) was adopted in both the unliganded PvNMT[WT] and [G386E] enzymes, and, on binding of DDD85646 to PvNMT[WT], Y211 moved only slightly to rotamer Y211b when DDD85646 was bound to PvNMT[G386E]. For PvNMT[WT] with bound IMP-1002, Y211 adopts rotamer Y211c, allowing the π-π stacking interaction with IMP-1002, which likely results in the increased potency and selectivity (over HsNMT) of this compound compared with DDD85646. However, for PvNMT[G386E] with bound IMP-1002, Y211 moves to rotamer position Y211d to avoid a steric clash with E386: this restricts the ability of Y211 to adopt its preferred conformation, preventing the π-π stacking interaction with IMP-1002 and leading to the shift of H213 from H213a to H213b. Figure 5D also shows the considerable flexibly of E386 in the different structures. We found, depending on the inhibitor bound (IMP-1002, DDD85646 or in the apo form), that E386 can adopt up to four distinct occupancies showing its flexible nature. Overall the minimal disruption of Y211 and H213 by DDD85646 binding results in the conserved affinity and binding mode for both WT and G386E variants (Figure 5C), and these data together with minimally perturbed IC_50_, EC_50_, and thermal stability (Figure 4; Tables S2, S3, and S5) lead us to conclude that DDD85646 can break resistance in NMT[G386E] parasites.

**Figure 6:**
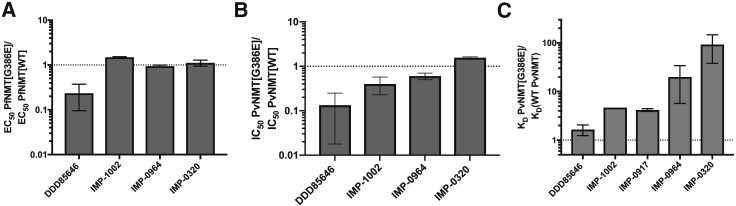
The NMT[G386E] Variant is Affected Differently by a Range of NMT Inhibitors (A) Mean increase of EC_50_ between PfNMT[G386E] and PfNMT[WT] parasites measured through SYBR Green growth assay (visualized on a log scale) (n = 2). A mean increase of 10 is indicated by a dotted line. (B) Enzymatic CPM assay measuring the mean increase in IC_50_ with different NMTi with purified PvNMT[WT] and PvNMT[G386E] (visualized on a log scale) (n = 3). A mean increase of 10 is indicated by a dotted line. (C) Mean increase in binding affinity of NMTi for PvNMT[WT] and PvNMT[G386E] (visualized on a log scale) (n = 1–3). The K_D_ of each compound with PvNMT[G386E] was divided by the K_D_ with PvNMT[WT] to calculate the fold difference. K_D_ is the equilibrium dissociation constant of NMTi calculated from fitted response-concentration plots measured by SPR analyses. Only one K_D_ could be accurately determined for IMP-1002 and so error bars are not shown. A mean increase of 10 is indicated by a dotted line. All errors bars in this figure indicate standard deviation of the mean.

To analyze further the binding mode of other NMT inhibitors, the published crystal structures of PvNMT[WT] with two bound NMTi, IMP-0320 (PDB: 2YNE) (Wright et al., 2014) and IMP-0856 (PDB: 4UFX) (Yu et al., 2015) (Figures 1 and S6B) were overlaid on the PvNMT[G386E] structure to examine whether the binding of these inhibitors would be affected by the G386E substitution. The movement of Y211 from position Y211c to Y211d, accompanied by the prevention of favorable interactions with both inhibitors, led us to hypothesize that their binding would also be affected by the G386E substitution, unlike DDD85646. To test this, we performed the same EC_50_ shift assay with the parasite and IC_50_ determination with the CPM assay using IMP-0964 (a close derivative of IMP-0856) and IMP-0320. We also included the NMTi IMP-0917, a close derivative of IMP-1002 that has recently been reported (Mousnier et al., 2018), which we expected to behave like IMP-1002 because of its similar structural properties (Figure S1). Shifts in EC_50_ (Figure 6A; Table S2) correlated very well with shifts in IC_50_ (Figure 6B; Table S3). These results confirmed that binding of both IMP-0320 and IMP-0964 were affected by the G386E substitution. In the same experiments, the potency of DDD85646 in killing parasites in the cell-based assay (Figure 6A) and binding in the enzyme assay (Figure 6B) was unaffected by the G386E change.

To complement the CPM assay and potentially increase the accuracy of measuring IC_50_ values below 10 nM in the case of highly potent compounds, we used surface plasmon resonance (SPR) to determine the relative K_D_ values of different NMTi with the two forms of the enzyme. SPR improved differentiation between the binding affinities of NMTi for PvNMT[WT] and PvNMT[G386E], as summarized in Figure S5C and Table S6. The affinities of IMP-1002 and IMP-0917 decreased by approximately 5-fold because of the substitution, whereas binding of DDD85646 was relatively insensitive. IMP-0320 and IMP-0964 showed the most marked decrease in affinity, almost 100- and 20-fold, respectively, between the PvNMT[G386E] and [WT] protein (Figures 6C and S5C; Table S6).

The kinetic differences between the compounds with the WT and G386E enzymes reflected this generally weaker binding, with faster off rates apparent from the sensorgram traces. IMP-1002 and IMP-0917 exhibited interesting kinetics: both showed a slow dissociation rate with PvNMT[WT] (Figure S5C), suggesting that several hours may be required for complete dissociation. Although the effect was largest for IMP-1002, the long dissociation rates of both compounds probably led to an underestimate of their affinities because of reduced binding capacity in sequential injections during a standard equilibrium K_D_ SPR analysis. In combination with the shift in EC_50_, IC_50_, and thermal stability, the SPR data clearly show that DDD85646 can overcome resistance mediated by G386E.

## Discussion

The current study describes identification and validation of the consequence of a single amino acid substitution (G386E) in the substrate-binding pocket of *Plasmodium* NMT, identified by drug selection of a non-synonymous point mutation in the *nmt* open reading frame. The importance of this mutation in *P. falciparum* parasites for this drug target, was confirmed by CRISPR-Cas9-mediated substitution of this single nucleotide in the WT parasite, recapitulating resistance against three chemically distinct NMT inhibitor series. Data from the PfNMT[G386E]-resistant parasite line were used to guide biochemical and structural analysis of this variant in recombinant PvNMT, providing both enzymatic inhibition data and the crystal structures of the WT and G386E variant with bound MyrCoA and the NMT inhibitor (NMTi) used for mutation selection. Structural, IC_50_ and K_D_ data for PvNMT[G386E] are consistent and correlated with the EC_50_ shift data obtained in G386E parasites, although the active site of PvNMT differs from PfNMT in 2 out of 23 residues (F212Y and F334Y, PfNMT to PvNMT) (Yu et al., 2012). These structural data provide insights into changes in the active site induced by the G386E substitution, including the modes of binding for different inhibitors and the flexibility required within the peptide-binding pocket to accommodate them. Using these data, a structure-guided approach was used to identify compounds that can overcome resistance mediated by the G386E substitution, exploiting the differential binding mode of DDD85646. This predictive structural model can guide further discovery of drugs active against NMT, and current work is focused on optimizing a new lead inhibitor series unaffected by G386E. The identification of NMTi that circumvent the G386E mutation suggests opportunities to combine different inhibitors to overcome selective resistance, and thus thwart the emergence of resistant variants.

Interestingly, the G386E variant parasite shows no apparent reduction of fitness in growth at the asexual blood stage, although it is currently unknown whether this observation would apply throughout the complete parasite life cycle. Even though G386E reduces the space in the peptide-binding pocket, the flexibility of Y211, E386, and H213 (Figure 5C) provides a plausible explanation for why substrate myristoylation is unaffected, leading to no reduction in fitness or change in global myristoylation (Figure S3). Future studies on the potential of various substrates with different sequences downstream of the N-terminal glycine to differentially stabilize intermediate states during the catalytic cycle of NMT, in combination with studies on the flexibility of turn-over of different substrates in the WT and G386E variant NMTs, could give further insights into substrate specificity.

Together, these data provide direct genetic evidence for the specific on-target activity of each of the NMTi classes described here, which, in combination with our previously reported chemical biology studies (Wright et al., 2014), further supports the validity of this target in malaria. The relatively fast selection of resistance to NMTi may suggest the potential for rapid development of resistance in the clinic. However, the SNP responsible for G386E is not present in any of the field isolates cataloged in PlasmoDB. In the 202 available isolates, there are 6 non-synonymous mutations in the coding region resulting in five amino acid changes, but these are all distal to the substrate-binding site and unlikely to interfere with NMTi binding. Furthermore, the repeated selection of this particular mutant in the Dd2 cloned line over a short period of drug pressure may imply the rapid acquisition of this mutation. We would therefore expect to see this mutation arising in the field when drug pressure is applied, considering that there was no fitness cost associated with G386E *in vitro*. Structural biology has been applied extensively to drug resistance in other fields including influenza (Wu et al., 2016), hepatitis C (Lagacé et al., 2011), and HIV (Slaughter et al., 2014), highlighting the general importance of structure-guided design for the discovery of resistance-breaking inhibitors.

In the future it would be of interest to attempt selection of parasites resistant to DDD85646, because the on-target activity of DDD85646 (compound 1a in Wright et al., 2014) rules out an off-target killing of PfNMT[G386E] parasites. DDD85646 is insensitive to the resistance mechanism generated in response to IMP-1002 treatment, but at a cost of lower selectivity over HsNMT (Table S5), because Y211 needs to move from the “in” Y211c rotamer occupancy, which provides improved selectivity over HsNMT (Yu et al., 2012), to the “out” Y211d rotamer, leaving room for G386E. Future studies will focus on identification of an inhibitor series that can overcome the G386E-mediated resistance without compromising selectivity, based on either IMP-1002 analogs or, more likely, a new chemical series with a distinct binding mode. For example, the series that includes IMP-1002 has recently been adapted to increase HsNMT selectivity with a relatively simple adaptation (Mousnier et al., 2018). This shows that the flexibility of the series has the scope to find analogs of IMP-1002 that can also overcome G386E-mediated resistance. In summary, this structure-guided analysis provides mechanistic rationalization and enables discovery of an NMTi that can overcome the identified NMT resistance mechanism resulting from the G386E substitution. This approach defines a strategy to overcome acquisition of NMTi resistance, and will facilitate further drug development efforts.

## Significance

**Malaria is one of the most significant infectious diseases worldwide, and the escalating threat of parasite resistance to all current antimalarial drugs highlights the need for drugs with new modes of action. The enzyme NMT is an antimalarial target, and here we provide genetic evidence for the on-target activity of several classes of NMT inhibitors through resistance selection. High-resolution crystal structures of NMT and the resistant variant provide deep insights into the flexibility of the substrate-binding pocket and allowed us to identify an inhibitor to overcome resistance. This work reveals the possibility of optimizing NMT inhibitors by incorporating resistance studies early in the drug discovery process, an approach with wider application in drug discovery and development.**

## STAR★Methods

### Key Resources Table

**Table:** 

REAGENT or RESOURCE	SOURCE	IDENTIFIER
**Antibodies**		

Rabbit anti GAP45 antibody used in Figure S2B.	Rabbit anti GAP45 antibody has been validated in: Rees-Channer et al., 2006	N/A

**Experimental Models: Cell Lines**		

Dd2 Plasmodium falciparum	Clone B2 from David Fidock's Lab - recloned from the Dd2 parental line by limiting dilution.	N/A
3D7 Plasmodium falciparum	parasite line originating from National Institute for Medical Research.	N/A

**Chemicals, Peptides, and Recombinant Proteins**		

YnMyr, AzTB	Heal et al., 2011	N/A
DDD85646	Frearson et al., 2010	N/A
IMP-0964	Yu et al., 2012	N/A
IMP-0856	Yu et al., 2012	N/A
IMP-0320	Wright et al., 2014	N/A
IMP-0917	Mousnier et al., 2018	N/A
IMP-1002	synthesis route in STAR Methods section of this study	N/A
Myristoyl coenzyme A lithium salt	Sigma Aldrich	Cat# M4414
Hoechst 33342 viability stain	New England biolabs,	Cat# 4082S
SYBR Green	Life Technologies	Cat# S7563
HsSrc_15AA_peptide	Synthesied at the Francis Crick Institute Nicola O'Reilly and Ganka Bineva-Todd	N/A
PvNMT G386E and PvNMT WT enzyme	expressed and purified by Alexandra R. Reers at Seattle Structural Genomics Center for Infectious Disease (SSGCID); Center for Global Infectious Disease Research, Seattle Children’s Research Institute	N/A
CPM	Sigma Aldrich	Cat# C1484

**Deposited Data**		

Crystal structure deposition	The PDB files that support the findings of this study have been deposited in Protein Data Bank	accession codes 6MAY (PvNMT G386E + MyrCoA), 6MAZ (PvNMT G386E, + MyrCoA + IMP-0366) 6MB0 (PvNMT G386E + MyrCoA + IMP-1002), and 6MB1 (PvNMT WT + MyrCoA + IMP-1002).
Crystal structure previously publsihed	The PDB files used in this study from previously published work	5O6H (PvNMT WT + MyrCoA + IMP-0917), 2YND (PvNMT WT + MyrCoA + IMP-0366), 2YNE PvNMT WT + MyrCoA + IMP-0320), 4UFX (PvNMT WT + MyrCoA + IMP-0856)

**Oligonucleotides**		

G386E-guide-01_F (Figure S3)	Sigma Aldrich Oligos	sequence: ATTGTTAAAATTTGGA GAAGGAGA
G386E-guide-01_R (Figure S3)	Sigma Aldrich Oligos	sequence: AAACTCTCCTTCTCC AAATTTTAA
G386E-guide-01_F (Figure S3)	Sigma Aldrich Oligos	sequence: ATTGTTTTAATGCCTTAG AAGTAA
G386E-guide-01_R (Figure S3)	Sigma Aldrich Oligos	sequence: AAACTTACTTCTAAGGC ATTAAAA
P1 (Figure S3)	Sigma Aldrich Oligos	sequence: TATGGCAAGCTATATATA CAGCAGG
P2 (Figure S3)	Sigma Aldrich Oligos	sequence: CCTCAGACTATATAACA TTAGAATG

**Software and Algorithms**		

Flow cytometry data collection in Figure 3B	FACSDiva software v8.0.1	N/A
Flow cytometry data analysis of Figure 3B	FlowJo 10.3.	N/A
CPM assay in Figures 4A and 6B	EnVision Workstation version 1.13.3009.1409	N/A
EC50 determination in Figures 4B and 6A	FLUOStar Omega Plate reader software was used from BMG Labtech	N/A
SPR data in Figure 6C	collected using Biacore T200 Evaluation Software v2.0	N/A
Immunofluorescence images Figure S2B	collected using the Nikon’s NIS Elements imaging software	N/A
Analysis of data from Figures 2, 3A, 3D, 4, 6, S2A, S2C, S4B, S5A, and S5C	Prism 7 GraphPad and Microsoft Excel	N/A
Chemical structure in Figures 1A and S1.	ChemDraw Professional 17.0	N/A
Crystallography in Figures 5, S6A, and S6B, Table S1	The structures were refined in Phenix, with manual model building in Coot. Quality of the models was assessed with MolProbity. Figures were generated using Pymol and CCP4MG. SPR data (Figure 6C) was analyzed using Biacore T200 Evaluation Software v2.0.	N/A

### Contact for Reagent and Resource Sharing

Further information and requests for resources and reagents should be directed to and will be fulfilled by the Lead Contact Prof. Edward W. Tate, e.tate@imperial.ac.uk.

### Experimental Model and Subject Details

#### Parasite Culture

*P. falciparum* 3D7 parasites were cultured *in vitro* in RPMI 1640 medium containing 0.5 % w/v Albumax II at 2-5 % hematocrit as described (Trager and Jensen, 2005). Parasites cultures were gassed with 90 % N_2_, 5 % CO_2_ and 5 % O_2_ and incubated at 37°C. Parasites were synchronized using 70 % Percoll gradients to purify schizont stages with a subsequent reinvasion followed by sorbitol treatment as described (Knuepfer et al., 2017).

### Method Details

#### Synthesis Route of IMP-1002

**Figure:**
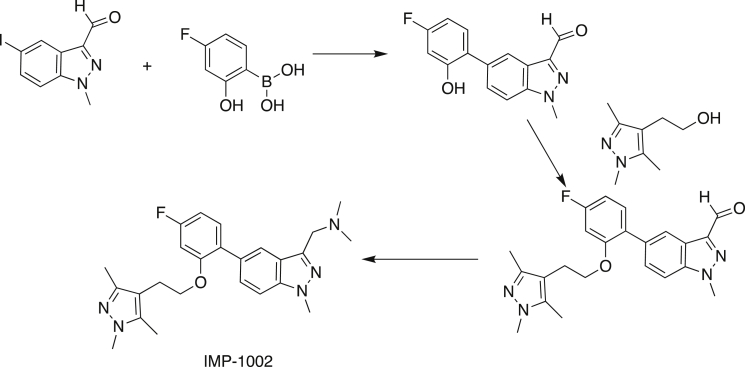

##### Step 1

A solution of 5-iodo-1-methyl-1*H*-indazole-3-carbaldehyde (500 mg, 1.7mmol) was dissolved in dioxane (5 mL) and treated with 4-fluoro-2-hydroxybenzene boronic acid (360 mg, 2.2 mmol) and tetrakis(triphenylphosphine) palladium(0) (20 mg), followed by a solution of potassium phosphate (560 mg, 2.6 mmol) in water (1 mL). The reaction mixture was heated under reflux for 2 h, cooled to room temperature and evaporated under reduced pressure. The residue was partitioned between EtOAc (20 mL) and saturated sodium bicarbonate solution (20 mL). The organic phase was dried over Na_2_SO_4,_ concentrated under reduced pressure and the crude product purified by flash column chromatography by elution with DCM/EtOAc (100:0, then 95:5) to give 5-(4-fluoro-2-hydroxyphenyl)-1methyl-1*H*-indazole-3-carbaldehyde as a colourless solid (450mg, yield: 95%); mp 212-214°C. ^1^H NMR (CD_3_OD, δ, ppm) 10.15 (s, 1H), 8.30 (d, *J* = 1.3 Hz, 1H), 7.73 (d, *J* = 1.5 Hz, 1H), 7.69 (s, 1H), 7.32 (dd, *J* = 9.2, 6.7 Hz, 1H), 6.74 – 6.64 (m, 2H), 4.62 (s, 1H), 4.24 (s, 3H).

##### Step 2

A solution of 5-(4-fluoro-2-hydroxyphenyl)-1-methyl-1*H*-indazole-3-carbaldehyde (135 mg; 0.5 mmol) and triphenylphosphine (262 mg; 1.0 mmol) in THF (4 mL) was treated with a solution of 2-(1,3,5-trimethyl-1*H*-pyrazol-4-yl)ethanol (154 mg, 1.0 mmol) in THF (3 mL). The reaction mixture was cooled to 0°C before being treated with di-isopropyl azodicarboxylate (291 mL, 1.48 mmol). The reaction mixture was allowed to warm to room temperature, stirred overnight and concentrated under reduced pressure. The crude product was purified by column chromatography by elution with hexane/IPA (gradient 80:20 to 70:30) to give a mixture of the desired product and unreacted alcohol. The crude product was further purified by elution with DMC/methanol (97:3) to give 5-(4-fluoro-2-(2-(1,3,5-trimethyl-1H-pyrazol-4-yl)ethoxy)phenyl)-1-methyl-1H-indazole-3-carbaldehyde as a colourless oil (86 mg, 42%). ^1^H NMR (CDCl_3_, δ, ppm) 10.22 (s, 1H), 8.37 (d, *J* = 1.5 Hz, 1H), 7.57 (dd, *J* = 8.8, 1.5 Hz, 1H), 7.46 (d, *J* = 8.7 Hz, 1H), 7.29 (dd, *J* = 8.4, 6.8 Hz, 1H), 6.72 (td, *J* = 8.3, 2.5 Hz, 1H), 6.67 (dd, *J* = 11.1, 2.4 Hz, 1H), 4.21 (s, 3H), 3.94 (t, *J* = 7.2 Hz, 2H), 3.62 (s, 3H), 2.74 (t, *J* = 7.1 Hz, 2H), 2.04 (s, 3H), 1.95 (s, 3H).

##### Step 3

A solution of 5-(4-fluoro-2-(2-(1,3,5-trimethyl-1H-pyrazol-4-yl)ethoxy)phenyl)-1-methyl-1H-indazole-3-carbaldehyde (43 mg, 0.11 mmol) in THF (3 mL) was treated with a solution of dimethylamine in THF (2 M, 160 mL 0.33 mmol), followed by glacial acetic acid (145 mL, 0.66 mmol). The solution was stirred at room temperature for 10 min before addition of solid sodium triacetoxy-borohydride (70 mg, 0.33 mmol) and DCE (2 mL). The reaction mixture was stirred at room temperature for 18 h before being quenched with sodium carbonate solution (2 M, 5 mL). DCM (20 mL) was added and the layers were separated. The organic extract was dried (Na_2_SO_4_) and concentrated under reduced pressure. The crude product was purified by column chromatography by elution with EtOAc/diethylamine (95:5) to provide **1**-(5-(4-fluoro-2-(2-(1,3,5-trimethyl-1H-pyrazol-4-yl)ethoxy)phenyl)-1-methyl-1H-indazol-3-yl)-N,N-dimethylmethanamine as a colourless oil (13.5 mg, 31%). LC-MS Rt = 11.6min, MH^+^ 436; ^1^H NMR (CDCl_3_, δ, ppm) 7.88 (d, *J* = 1.8 Hz, 1H), 7.48 (dd, *J* = 8.7, 1.5 Hz, 1H), 7.35 (d, *J* = 8.7 Hz, 1H), 7.33 – 7.26 (m, 1H), 6.73 (td, *J* = 8.3, 2.5 Hz, 1H), 6.67 (dd, *J* = 10.8, 2.4 Hz, 1H), 4.08 (s, 3H), 3.93 (t, *J* = 7.2 Hz, 2H), 3.83 (s, 2H), 3.63 (s, 3H), 2.75 (t, *J* = 7.3 Hz, 2H), 2.33 (s, 6H), 2.08 (s, 3H), 1.92 (s, 3H); ^13^C NMR (CDCl_3_, δ, ppm) 162.76 (d, *J* = 245.2 Hz), 156.81 (d, *J* = 9.9 Hz), 145.75, 142.38, 140.07, 136.91, 131.89 (d, *J* = 10.0 Hz), 130.14, 128.66, 127.19, 123.46, 121.30, 111.62, 108.19, 107.20 (d, *J* = 21.0 Hz), 100.39 (d, *J* = 25.8 Hz), 68.63, 55.81, 45.59, 35.75, 35.41, 23.66, 11.71, 9.34; ESI HRMS, found 436.2510 (C_25_H_31_FN_5_O, [M + H]^+^, requires 436.2513).

#### Indirect Immunofluorescence Assay (IFA)

For IFA, slides were air dried, fixed in 4 % paraformaldehyde (PFA) in PBS for 10–20 min, permeabilized in 0.1 % Triton X-100 in PBS for 10 min, and blocked with 3 % BSA in PBS for at least 30 min at 4°C. GAP45 detection was achieved using a primary rabbit anti-GAP45-antibody (Rees-Channer et al., 2006). Slides were mounted in ProLong® Gold Antifade mounting medium containing DAPI (4’,6-diamidino-2-phenylindole), and viewed on a Nikon Eclipse Ni-E imaging system with a Hamamatsu Orca-flash 4.0 digital camera and a Plan apo λ 100x/1.45 oil immersion objective. Images were captured using Nikon’s NIS-Elements imaging software generating Z-stack images of individual parasites, using deconvolution options and exporting the image as a tiff file, which was further edited using Adobe Photoshop.

#### Drug Treatment and EC_50_ Determination Using the SYBR Green Assay

Aliquots of 100 μL ring-stage parasites (1–6 h post invasion [PI]) were diluted in 96-well flat bottom black plates to 0.3 % parasitemia and 2 % hematocrit. Inhibitors were added in doubling dilutions ranging from 7.8 nM – 5 μM for DDD85646 and 0.54 nM – 400 nM for IMP-1002 for the initial EC_50_ determination in 3D7 in a final volume of 100 μL. For the EC_50_ of PfNMT[G386E] compared to PfNMT[WT] a range from 0.2 nM – 10 μM for IMP-1002 and DDD85646 and 0.6 nM - 40 μM for IMP-0964 and IMP-0320 was used. The final DMSO percentage of 0.08% did not interfere with parasite development determined by Giemsa-stained thin blood smears. Cultures were incubated in gassed humidity chambers (90 % N_2_, 5 % CO_2_ and 5 % O_2_) at 37°C for a further 96 h to increase the dynamic range and processed as described (Green et al., 2015). After 96 h, parasites were lysed and DNA was stained by adding to each well 25 μl lysis buffer (20 mM Tris-HCl, pH 8.0, 2 mM EDTA, pH 8.0, 1.6 % Triton X-100, 0.16 % saponin, containing 0.1% SYBR Green [Life Technologies]) (final SYBR Green concentration of 0.02%) (Smilkstein et al., 2004). Plates were incubated for 1 h in the dark at room temperature (RT) before measurements on a FLUOStar Omega Plate reader (BMG Labtech) with excitation and emission filters of 485 nm and 520 nm, respectively. EC_50_ values were calculated from a four-parameter logistical fit of the data using Prism software (GraphPad Software, Inc.), with subtraction of the background signal from uninfected RBC. When comparing the EC_50_ of two parasite lines, Prism software was used to fit and constrain EC_50_ curves at the top and bottom to improve plateau definition through a shared value of all data sets.

#### Selection of IMP-1002-Resistant *P. falciparum* Dd2 Parasites Using a Single-Step Strategy

Prior to selection, genomic DNA was harvested and a frozen stock of the parental Dd2 parasite line was generated. Selection was initiated with three wells of 10^6^ parasite inocula and three wells of 10^7^ parasite inocula. A final concentration of 120 nM IMP-1002 (3.4 x the 35 nM EC_50_) was added to a predominantly ring stage culture. The culture was fed daily with drug-containing complete medium for six days and every other day thereafter. The parasitemia was monitored and kept below 10 % over the next days to prevent overgrowth. Fresh RBCs (equivalent to 0.5 % hematocrit) were added at day 7. At day 14, and weekly thereafter, the cultures were passaged (¾ cultured cells were replaced ¼ with fresh RBC and new medium) to prevent lysis. Drug pressure was maintained for 60 days.

#### NMT gDNA Amplification and Sequencing from IMP-1002 Resistant *P. falciparum*

The genomic DNA sequence of the *nmt* gene in *P. falciparum* 3D7 and Dd2 is identical (www.PlasmoDB.org) and was used for primer design. Following PCR amplification, the product was sequenced and cloned into pGEM-T Easy vector after ligation of a 3'-A overhang using Amplitaq polymerase to improve the sequence quality. Sequence data were analyzed using DNASTAR SeqMAN Pro and a consensus sequence for each of the parasite lines was fed into CLC Sequence Viewer 7 to create a nucleotide alignment. The nucleotide sequence was also translated into protein, which was aligned to the WT sequence using CLC Sequence Viewer 7.

#### Cloning of Constructs and Transfection of 3D7 *P. falciparum*

The construct to generate the G386E NMT mutation was generated using Gene Art and provided on a pMK-RQ kanamycin resistance plasmid. The two independent guides were designed using protospacer.com and cloned into the pDC2-cam-Cas9-U6-hDHFRyFCU-plasmid (Knuepfer et al., 2017, Lim et al., 2016, Macpherson and Scherf, 2015). Guide and rescue plasmids were paired and ethanol precipitated prior to transfection. Asexual blood-stage cultures of *P. falciparum* clone 3D7 were maintained *in vitro* and synchronized according to standard procedures (Gerold et al., 1996, Yeoh et al., 2007). Transfection DNA was added to mature schizonts, enriched from highly synchronous cultures using Percoll (GE Healthcare) gradients as described (Harris et al., 2005). Parasites were transfected by electroporation using the Amaxa 4D electroporator (Lonza) and the P3 Primary cell 4D Nucleofector X Kit L (Lonza) and program FP158, as recently described for *P. knowlesi* (Moon et al., 2013). Electroporations were performed with 60 μg of linearized rescue plasmid and 20 μg of the CRISPR/Cas9 plasmid carrying the respective guide RNA. Selections were carried out as recently described (Knuepfer et al., 2017). Parasites were cultured in the presence of 2.5 nM WR99210 drug pressure for five days to select for parasites with the Cas9/guide plasmid. Transfected parasites reappeared after 17 days. After 29 days, positive selection with 1 μM ancotil was initiated to minimize the existence of excess Cas9/guide plasmid. Transfected parasites were cloned by limiting dilution after 37 days (Rosario, 1981) and screened for single parasites with a plaque assay (Thomas et al., 2016). Individual clones were screened for integration by PCR amplification and digestion with BglII (guide 1 transfection) and XhoI (guide 2 transfection).

#### Parasite Growth Assay

Analysis of the growth of WT and two NMT[G386E] parasite clones over eight generations was performed by first equalizing the starting parasitemia (0.67 % WT, 0.73 % Clone 1 and 0.61 % Clone 2). Three biological replicates were generated in three different blood dilutions with three technical replicates each. WT parasitemia was determined following Giemsa staining after each cycle and used to determine the dilution factor to dilute all three lines down to 0.8%. Parasitemia was measured after each cycle using flow cytometry and Hoechst-stained parasites. Multiple t-tests were performed for each generation (two-stage linear step-up procedure of Benjamini, Krieger and Yekutieli, with false-discovery rate = 1 %).

#### Metabolic Tagging of Parasites and CuAAC Labelling

Synchronized populations of stage-specific parasites were labelled with 25 μM of YnMyr for the specified length of time. Parasites were then lysed in 0.15 % saponin for 10 min on ice. The pellet was washed until the supernatant was colorless and then stored at -80°C. Thawed parasite pellets were extracted in 1 % Triton X-100, 0.1 % SDS in PBS on ice for 20 min with 1 min sonication. The protein concentration of the lysate was measured by BCA assay and adjusted to 1 mg/mL with PBS. The capture reagent was ‘clicked’ (100 μM capture reagent AzRB2, 1 mM CuSO_4_, 1 mM TCEP, 100 μM TBTA), the reaction was quenched with EDTA, and then precipitated proteins were washed with ice cold methanol as described previously (Heal et al., 2011, Wright et al., 2015). Proteins were re-dissolved in 2 % SDS, 10 mM DTT, in PBS at 1 mg/mL and heated for 7 min at 95°C in sample loading buffer prior to SDS-PAGE.

#### Base Treatment to Cleave the Esters of GPI-Anchored Proteins

Following the click reaction, the protein solution in PBS containing 2 % SDS, 10 mM DTT was treated with 0.2 M NaOH for 1 h at room temperature with vortexing. Samples were neutralized with an equivalent volume and concentration of HCl and diluted with PBS to 1 mg/mL prior to gel analysis.

#### Flow Cytometry

Synchronized parasites were incubated with DMSO or NMTi and samples were fixed in 2% PFA, 0.2 % glutaraldehyde for 1 hour at 45 h PI. Subsequently samples were washed in PBS and labelled with 1:500 of 10 mg/mL Hoechst 33342 for 10 min with a subsequent wash in PBS. For analysis, a Flow Cytometry CL1 Fortessa D Analyzer from BD with FACSDiva software v8.0.1 was used with the 450-50 filter counting 50,000 RBC per sample. Data were analyzed using FlowJo LLC 2006-2015 to determine the median fluorescence intensity (MFI) of each sample after gating out the RBC population using an RBC only sample as a control. The MFI of each sample was divided by the MFI of a control sample of synchronized rings with a known MFI of one nucleus.

#### Cloning, Expression and Purification of PvNMT

The purification method has been adapted from earlier work (Bryan et al., 2011, Choi et al., 2011). A region of the PvNMT gene encoding residues 27-410 and tagged at the N-terminus with a hexaHis sequence was cloned into a pET11a expression vector. The G386E mutation was generated using QuickChange II mutagenesis by exchanging base pairs 1079 and 1080 of the PvNMT open reading frame from GC to AA, which changes glycine at position 386 to glutamic acid. The constructs were confirmed by sequencing and the plasmid DNAs were used to transform Rosetta-gami (DE3) *E. coli* competent cells, using ampicillin for pET11a selection and chloramphenicol for the pRARE2 plasmid present in the Rosetta-gami DE3 cells to supply the tRNAs for rare codons used to codon-optimize PvNMT. PvNMT[WT] and PvNMT[G386E] were expressed following inoculation of a single colony overnight at 37°C in 5 mL Lysogeny Broth. The PvNMT[WT] protein was expressed in 8 x 500 mL auto-induction medium (92 % ZY, 0.1 % 1M MgSO_4_, 0.1 % 1000x Trace Metals, 5 % 20xNPS, 2 % 50x 5052) (2x 500mL for small scale) and the PvNMT[G386E] protein in 19 L due to low yield. Auto-induction medium was combined with appropriate antibiotics and incubated with 1:100 dilution factor from the overnight culture at 37 C for 4 h. Temperature was then reduced to 18°C for 18-22 h. Bacterial cells were harvested by centrifugation and pellets from 4 L (for the WT protein) re-suspended in 180 mL of lysis buffer (20 mM HEPES, 5 % glycerol, 10 mM imidazole, 0.5 % CHAPS, 21 mM MgCl_2_, 1 mM DTT and Complete Protease Inhibitor Cocktail EDTA-free). For the PvNMT[G386E] protein cells from the 15 L were resuspended in 2x 300 mL, sonicated separately and then combined prior to application to the column. They were lysed using a BRANSON 550 sonication probe (20 % strength, 5 sec on 10 sec off for 10 min, 15 min total of sonication). The lysate was cleared by centrifugation at 48,384*g* RCF for 60 min at 4°C in a 25.50 JA rotor.

For large scale preparations the lysate was filtered through a 0.45 μM syringe filter and applied to a HisTRAP FF 5 mL column at 4°C and eluted with a linear gradient over 15 column volumes with 20 mM HEPES, 0.3 M NaCl, 5 % glycerol, 0.5 M imidazole; pH 7.0. Fractions containing the target protein were pooled and concentrated to about 2-3 mL using 10 kDa-cut off Pall centrifuge concentrators.

For small scale preps the lysate was incubated in batch with 20 mL of Ni-NTA Agarose beads (QIAGEN) equilibrated with lysis buffer in a 500 mL Duran bottle at 4°C for 1 hour. The beads were washed using a re-usable glass Fritted Funnel column and a pressure pump with 20x bead volume of wash buffer using a pressure pump (20 mM HEPES, 5 % glycerol, 30 mM imidazole, 0.5 % CHAPS, 21 mM MgCl_2_). The bound protein was eluted from the beads using 3x bead volume of elution buffer through gravity (0.3 M NaCl, 20 mM HEPES, 5 % glycerol, 0.5 M imidazole; pH 7.0). Eluted proteins were analyzed using instant blue (Expedeon Prod # ISB1L). Nickel elution was filtered and concentrated using Centricon Plus 70 with a 30kDa cut-off.

For both small and large scale preparations concentrated protein was diluted 100-fold with anion exchange buffer (20 mM Tris–HCl, 1 mM TCEP; pH 8.9) and applied directly to a HiTRAP Q HP 5 mL column (1 mL column for small scale) at 2 mL/min. Unbound sample was washed away with 5-8 column volumes (CV) at 2 mL/min for the 1 mL column and 3 mL/min for the 5mL column and eluted with 2 %-50 % of the elution buffer over 15 CV at 0.5 mL/min for the 1 mL column and 1 mL/min for the 5mL column. The 2 mL fraction (5 mL column) or 0.5 mL fractions (1 mL column) were analysed by applying 10 μL to an SDS PAGE gel and visualized using instant blue. For protein for crystallography, the His tag was cleaved from the PvNMT[WT] and PvNMT[G386E] proteins via 3C protease (His-MBP-3C) at a 1:50 protease: protein ratio overnight at 4°C in dialysis buffer (25 mM HEPES pH 7.5, 500 mM NaCl, 5 % (v/v) glycerol, 1 mM TCEP, and 0.025 % sodium azide). NMT was recovered by applying the dialysate to a second IMAC column. Fractions containing target protein were pooled and concentrated to 70 μL for the small scale or 4 mL for large scale and loaded onto Superdex 75 10/300 or 26/600 columns and eluted with size exclusion buffer (0.3 M NaCl, 20 mM HEPES, 5 %(v/v) glycerol, 1 mM TCEP; pH 7.0). Fractions were analyzed on an SDS-PAGE gel and fractions containing the target protein were either pooled in the case of PvNMT[WT] or because of limited material kept separate for the PvNMT[G386E] protein. Fractions were concentrated and flash frozen and stored at – 80°C until further use.

For small scale expression the purified protein was detected by Western blotting, using the anti-PvNMT antibody (PvNMT protein made by Jim Brannigan at York University and antibodies raised by David Moss, MRC National Institute for Medical Research). For Western blots, proteins were transferred from the gel to nitrocellulose membrane with the iBlot™ Transfer System. Following blocking overnight at 4°C, membranes were incubated with primary antibody for 1 h at room temperature in blocking solution and incubated with secondary antibody (goat-anti-rabbit/rat/mouse IgG-HRP, Invitrogen 1:2500) for 1 h in blocking solution. Detection was carried out using GE Healthcare ECL Western Blotting Detection reagents or BioRad Clarity Western ECL Substrate and visualized on a ChemiDoc MP BIO-RAD Imaging System.

#### CPM-Based PvNMT IC_50_ Determination

To measure the activity of the purified PvNMT[WT] and PvNMT[G386E], an assay was used with thiol-selective fluorescent dye (CPM) to monitor the formation of coenzyme A. The assay was performed as published in (Goncalves et al., 2012a). Reagents were prepared in 20 mM sodium phosphate, 0.5 mM EDTA, 0.1 % Triton X-100 at pH 7.90-7.95 with the corresponding DMSO concentrations. First, 10 μL of a 10 % DMSO/water (v/v) solution containing a dilution series of NMT inhibitor, 50 μL of NMT in 1 % DMSO (final concentration of 6.3 nM), and 25 μL of myristoyl–CoA solution in 1 % DMSO (4 μM final concentration), were combined in Black microplate 96-well non-sterile flat bottom polypropylene plates (655209). The reaction was started by adding 25 μL of CPM and a 15-residue HsSrc peptide solution in 5 % DMSO (final concentration CPM 8 μM, HsSrc peptide 4 μM). For the continuous assay the fluorescence intensity was monitored over 45 min at 0.5-min intervals (excitation at 380 nm, emission at 470 nm) at 25°C using the EnVision Xcite Plate Reader (exported with EnVision Workstation version 1.13.3009.1409). For the quenched assay the reaction was stopped after 30 min at which point the initial reaction rate was still in the linear range by adding 60 μL of the Quench Solution (50 mM acetic acid, 50 mM sodium acetate, pH 4.74-4.78).

#### Thermal Shift Assay

Purified recombinant PvNMT[WT] and PvNMT[G386E] were diluted to 0.1 mg/mL in PBS buffer containing 10% glycerol and 1:1000 dilution of Sypro Orange in the presence or absence of 4 μM myristoylCoA. Test compounds were diluted to 500 μM in DMSO, and 0.5 μL of diluted compound was added to 49 ul of enzyme in a 96-well plate resulting in two technical replicates with a final volume of 20 μL and 1% (final) DMSO with a compound concentration of 5 μM. Each compound was tested twice to give two experimental replicates, each with two technical replicates. Reference thermal melting temperatures (Tms) were obtained using 1% DMSO without any compound present. The plates were subjected to a temperature gradient from 300K to 348K at a rate of 1K/min using a FluoDia fluorescent plate reader. Fluorescence data were acquired with an excitation filter of 486nm and an emission filter at 610nm, and the raw data were exported to Prism or Excel for analysis. Data were processed to identify the fluorescence maxima and minima, and the midpoints of melting curves were determined by fitting to a Boltzmann equation, where Tm is the temperature corresponding to the half maximum response. (Y=y_min_ + (y_max_-y_min_)/ (1+exp ((Tm-T)/slope)). Results were expressed as ΔTm in °C, where ΔTm is the difference between Tm in the presence of compound subtracted from that in the reference without compound present.

#### Surface Plasmon Resonance (SPR) Assay

The surface plasmon resonance experiments were performed using a Biacore T200 (GE Healthcare) equipped with research-grade CM5 sensor chips. PvNMT (5.14 mg/mL, 45.0 kDa, 20 mM HEPES pH 7.0, 300 mM NaCl, 5 % glycerol, 1 mM TCEP) was diluted to 0.0514 mg/mL in 10 mM sodium acetate pH 6.0. PvNMT[G386E] (0.95 mg/mL, 47.3 kDa, 20 mM HEPES pH 7.0, 300 mM NaCl, 5 % glycerol, 1 mM TCEP) was diluted to 0.0428 mg/mL in 10 mM sodium acetate pH 6.0. Each ligand was immobilized onto separate flow cells using amine-coupling chemistry. The surfaces of all four flow cells were activated for 7 min with a 1:1 mixture of 0.1 M NHS (N-hydroxysuccinimide) and 0.1 M EDC (3-(N,N-dimethylamino) propyl-N-ethylcarbodiimide) at a flow rate of 10 μL/min. PvNMT was immobilized at a density of 900 - 1100 RU onto flow cell 3, and PvNMT[G386E] at 600-1700 RU onto flow cell 4. Flow cell 1 was left blank to serve as a reference surface. All four surfaces were blocked with a 7 min injection of 1 M ethanolamine, pH 8.0. IMP-0320 was used as a positive control in the assay as it has been extensively used by GSK for routine NMT SPR assays.

The analytes (IMP compounds [400-550 Da] dissolved in DMSO to a concentration of 500 μM,) were serially diluted 1:3 in DMSO, and then 1-3 μL of each dilution was dissolved in an assay buffer of 10 mM HEPES pH 7.4, 150 mM NaCl, 0.005 % Tween 20, 4 μM myristoylCoA to a final DMSO concentration of 1 %. To collect binding affinity data, the analyte concentrations (0-0.4 μM except from DDD85646: 0-1 μM) were injected sequentially over all four flow cells at a flow rate of 30 μL/min and at a temperature of 25°C. The complexes formed were allowed to associate for 200-700 sec and then dissociate for 600-2400 sec, dependent on the analyte kinetics observed. The surfaces were washed with an injection of 50 % DMSO between each analyte injection. The data were analyzed within Biacore T200 Evaluation Software v2.0.

Equilibrium sensorgram values were exported and the offset set to zero before fitting to a global ‘One site – Specific binding’ nonlinear regression model (Motulsky and Brown, 2006) in GraphPad Prism 7. As differential binding of compounds to PvNMT[G386E] and PvNMT[WT] were of greatest interest, the ratio of K_D_ PvNMT[G386E] to that of PvNMT [WT] was calculated for each compound dose response on the same chip within an experiment.

#### Crystallography

PvNMT[WT] and PvNMT[G386E] were crystallized using the sitting drop vapor diffusion method at 7.8 mg/mL and 12.2 mg/mL respectively against a focused screen generated from a published literature condition (Yu et al., 2012). Drops contained 0.4 μL protein (supplemented with either 0.5 mM MyrCoA or 0.5 mM MyrCoA and 0.5 mM inhibitor) and 0.4 μL crystallant. Drops were equilibrated against 80 μL of the crystallant in Compact Jr crystallization trays (Rigaku Reagents) and incubated at 16°C. Large, rod-morphology crystals appeared between one and two weeks and were cryo-protected with 20-25 % (v/v) ethylene glycol supplemented with ligand(s) prior to vitrification in liquid nitrogen for X-ray data collection.

X-ray diffraction data was collected at the LS-CAT beamline 21-ID-F at the Advanced Photon Source. Collection information and processing statistics are reported in Supplemental Information appendix Table S1. The data were integrated with XDS and reduced with XSCALE (Kabsch, 2010). Data quality assessment and space group assignment were performed using POINTLESS (Evans, 2006). Structures were solved by molecular replacement using MoRDa (Vagin and Lebedev, 2015), using PvNMT chain A (PDB accession code 4B14) in the same crystal form as a search model. The structures were refined in Phenix (Adams et al., 2010), with manual model building in Coot (Emsley and Cowtan, 2004). Quality of the models was assessed with MolProbity (Chen et al., 2010). Figures were generated using Pymol (Schrodinger, 2014) and CCP4MG (McNicholas et al., 2011). Inhibitor omit maps (Figure S6A) were generated by complete removal of inhibitor atoms from fully refined structures and running an additional refinement cycle in Phenix. Coordinates and structure factors have been deposited in the Protein Data Bank under accession codes 6MAY, 6MAZ, 6MB0, and 6MB1.

### Quantification and Statistical Analysis

Data were analyzed using Prism 7.0 by GraphPad. Quantitative data are presented as mean ± standard deviation. Dose response curve fitting in Figures 2, 3A, 4A, 4B, 6A, 6B, and S2A, to calculate IC_50_s EC_50_, Hill coefficients and confidence intervals, was done using four-parameter [Inhibitor] vs. response. For KD determination through SPR in Figures 4C and 6C a ‘One site – Specific binding’ nonlinear regression model (Motulsky and Brown, 2006) was used. Replicate number and definition of n is indicated in figure legends. For Figure 3A an unpaired Welch T-test was performed not assuming equal SDs. For Figure S4B a multiple t-tests was performed for each parasite cycle (two-stage linear step-up procedure of Benjamini, Krieger and Yekutieli, with false-discovery rate = 1 %).

### Data and Software Availability

The PDB files that support the findings of this study have been deposited in Protein Data Bank under accession codes 6MAY, 6MAZ, 6MB0, and 6MB1.

## Supplemental Information

Document S1. Figures S1–S6 and Tables S1–S6

Document S2. Article plus Supplemental Information

## References

[bib1] Adams P.D., Afonine P.V., Bunkóczi G., Chen V.B., Davis I.W., Echols N., Headd J.J., Hung L.-W., Kapral G.J., Grosse-Kunstleve R.W. (2010). PHENIX: a comprehensive Python-based system for macromolecular structure solution. Acta Crystallogr. D Biol. Crystallogr..

[bib2] Ariey F., Witkowski B., Amaratunga C., Beghain J., Langlois A.-C., Khim N., Kim S., Duru V., Bouchier C., Ma L. (2014). A molecular marker of artemisinin-resistant *Plasmodium falciparum* malaria. Nature.

[bib3] Bell A.S., Mills J.E., Williams G.P., Brannigan J.A., Wilkinson A.J., Parkinson T., Leatherbarrow R.J., Tate E.W., Holder A., Smith D.F. (2012). Selective inhibitors of protozoan protein N-myristoyltransferases as starting points for tropical disease medicinal chemistry programs. PLoS Negl. Trop. Dis..

[bib4] Brannigan J.A., Roberts S.M., Bell A.S., Hutton J.A., Hodgkinson M.R., Tate E.W., Leatherbarrow R.J., Smith D.F., Wilkinson A.J. (2014). Diverse modes of binding in structures of *Leishmania* major N-myristoyltransferase with selective inhibitors. IUCrJ.

[bib5] Brannigan J.A., Smith B.A., Yu Z., Brzozowski A.M., Hodgkinson M.R., Maroof A., Price H.P., Meier F., Leatherbarrow R.J., Tate E.W. (2010). N-Myristoyltransferase from *Leishmania donovani*: structural and functional characterisation of a potential drug target for visceral leishmaniasis. J. Mol. Biol..

[bib6] Bryan C.M., Bhandari J., Napuli A.J., Leibly D.J., Choi R., Kelley A., Van Voorhis W.C., Edwards T.E., Stewart L.J. (2011). High-throughput protein production and purification at the Seattle structural genomics center for infectious disease. Acta Crystallogr. Sect. F Struct. Biol. Cryst. Commun..

[bib7] Chen V.B., Arendall W.B., Headd J.J., Keedy D.A., Immormino R.M., Kapral G.J., Murray L.W., Richardson J.S., Richardson D.C. (2010). MolProbity: all-atom structure validation for macromolecular crystallography. Acta Crystallogr. D Biol. Crystallogr..

[bib8] Choi R., Kelley A., Leibly D., Hewitt S.N., Napuli A., Van Voorhis W. (2011). Immobilized metal-affinity chromatography protein-recovery screening is predictive of crystallographic structure success. Acta Crystallogr. Sect. F Struct. Biol. Cryst. Commun..

[bib9] Cowell A.N., Istvan E.S., Lukens A.K., Gomez-Lorenzo M.G., Vanaerschot M., Sakata-Kato T., Flannery E.L., Magistrado P., Owen E., Abraham M. (2018). Mapping the malaria parasite druggable genome by using in vitro evolution and chemogenomics. Science.

[bib10] Emsley P., Cowtan K. (2004). Coot: model-building tools for molecular graphics. Acta Crystallogr. D Biol. Crystallogr..

[bib11] Evans P. (2006). Scaling and assessment of data quality. Acta Crystallogr. D Biol. Crystallogr..

[bib12] Fang W., Robinson D.A., Raimi O.G., Blair D.E., Harrison J.R., Lockhart D.E.A., Torrie L.S., Ruda G.F., Wyatt P.G., Gilbert I.H. (2015). N-Myristoyltransferase is a cell wall target in *Aspergillus fumigatus*. ACS Chem. Biol..

[bib13] Flannery E.L., Fidock D.A., Winzeler E.A. (2013). Using genetic methods to define the targets of compounds with antimalarial activity. J. Med. Chem..

[bib14] Frearson J.A., Brand S., McElroy S.P., Cleghorn L.A.T., Smid O., Stojanovski L., Price H.P., Guther M.L.S., Torrie L.S., Robinson D.A. (2010). N-Myristoyltransferase inhibitors as new leads to treat sleeping sickness. Nature.

[bib15] Gerold P., Schofield L., Blackman M.J., Holder A.A., Schwarz R.T. (1996). Structural analysis of the glycosyl-phosphatidylinositol membrane anchor of the merozoite surface proteins-1 and -2 of *Plasmodium falciparum*. Mol. Biochem. Parasitol..

[bib16] Ghorbal M., Gorman M., Macpherson C.R., Martins R.M., Scherf A., Lopez-Rubio J.J. (2014). Genome editing in the human malaria parasite *Plasmodium falciparum* using the CRISPR-Cas9 system. Nat. Biotechnol..

[bib17] Goncalves V., Brannigan J.A., Thinon E., Olaleye T.O., Serwa R., Lanzarone S., Wilkinson A.J., Tate E.W., Leatherbarrow R.J. (2012). A fluorescence-based assay for N-myristoyltransferase activity. Anal. Biochem..

[bib18] Goncalves V., Brannigan J.A., Whalley D., Ansell K.H., Saxty B., Holder A., Wilkinson A.J., Tate E.W., Leatherbarrow R.J. (2012). Discovery of *Plasmodium vivax* N-myristoyltransferase inhibitors: screening, synthesis, and structural characterization of their binding mode. J. Med. Chem..

[bib19] Green J.L., Moon R.W., Whalley D., Bowyer P.W., Wallace C., Rochani A., Kumar R., Howell S.A., Grainger M., Jones H.M. (2015). Imidazopyridazine inhibitors of *Plasmodium falciparum* calcium-dependent protein kinase 1 also target cyclic GMP-dependent protein kinase and heat shock protein 90 to kill the parasite at different stages of intracellular development. Antimicrob. Agents Chemother..

[bib20] Harris P.K., Yeoh S., Dluzewski A.R., O'Donnell R.A., Withers-Martinez C., Hackett F., Bannister L.H., Mitchell G.H., Blackman M.J. (2005). Molecular identification of a malaria merozoite surface sheddase. PLoS Pathog..

[bib21] Heal W.P., Wright M.H., Thinon E., Tate E.W. (2011). Multifunctional protein labeling via enzymatic N-terminal tagging and elaboration by click chemistry. Nat. Protoc..

[bib22] Hutton J.A., Goncalves V., Brannigan J.A., Paape D., Wright M.H., Waugh T.M., Roberts S.M., Bell A.S., Wilkinson A.J., Smith D.F. (2014). Structure-based design of potent and selective *Leishmania* N-myristoyltransferase inhibitors. J. Med. Chem..

[bib23] Kabsch W. (2010). Integration, scaling, space-group assignment and post-refinement. Acta Crystallogr. D Biol. Crystallogr..

[bib24] Knuepfer E., Napiorkowska M., van Ooij C., Holder A. (2017). Generating conditional gene knockouts in *Plasmodium* - a toolkit to produce stable DiCre recombinase-expressing parasite lines using CRISPR/Cas9. Sci. Rep..

[bib25] Lagacé L., White P.W., Bousquet C., Dansereau N., Dô F., Llinas-Brunet M., Marquis M., Massariol M.-J., Maurice R., Spickler C. (2011). In vitro resistance profile of the hepatitis C virus NS3 protease inhibitor BI 201335. Antimicrob. Agents Chemother..

[bib26] Lim M.Y.-X., LaMonte G., Lee M.C.S., Reimer C., Tan B.H., Corey V., Tjahjadi B.F., Chua A., Nachon M., Wintjens R. (2016). UDP-galactose and acetyl-CoA transporters as *Plasmodium* multidrug resistance genes. Nat. Microbiol..

[bib27] Macpherson C.R., Scherf A. (2015). Flexible guide-RNA design for CRISPR applications using Protospacer Workbench. Nat. Biotechnol..

[bib28] McNicholas S., Potterton E., Wilson K.S., Noble M.E.M. (2011). Presenting your structures: the CCP4mg molecular-graphics software. Acta Crystallogr. D Biol. Crystallogr..

[bib29] Moon R.W., Hall J., Rangkuti F., Ho Y.S., Almond N., Mitchell G.H., Pain A., Holder A., Blackman M.J. (2013). Adaptation of the genetically tractable malaria pathogen *Plasmodium knowlesi* to continuous culture in human erythrocytes. Proc. Natl. Acad. Sci. U S A.

[bib30] Motulsky H.J., Brown R.E. (2006). Detecting outliers when fitting data with nonlinear regression - a new method based on robust nonlinear regression and the false discovery rate. BMC Bioinformatics.

[bib31] Mousnier A., Bell A.S., Swieboda D.P., Morales-Sanfrutos J., Pérez-Dorado I., Brannigan J.A., Newman J., Ritzefeld M., Hutton J.A., Guedán A. (2018). Fragment-derived inhibitors of human N-myristoyltransferase block capsid assembly and replication of the common cold virus. Nat. Chem..

[bib32] Olaleye T.O., Brannigan J.A., Roberts S.M., Leatherbarrow R.J., Wilkinson A.J., Tate E.W. (2014). Peptidomimetic inhibitors of N-myristoyltransferase from human malaria and leishmaniasis parasites. Org. Biomol. Chem..

[bib33] Perrin A.J., Collins C.R., Russell M.R.G., Collinson L.M., Baker D.A., Blackman M.J. (2018). The actinomyosin motor drives malaria parasite red blood cell invasion but not egress. MBio.

[bib34] Rackham M.D., Brannigan J.A., Rangachari K., Meister S., Wilkinson A.J., Holder A., Leatherbarrow R.J., Tate E.W. (2014). Design and synthesis of high affinity inhibitors of *Plasmodium falciparum* and *Plasmodium vivax* N-myristoyltransferases directed by ligand efficiency dependent lipophilicity (LELP). J. Med. Chem..

[bib35] Rackham M.D., Yu Z., Brannigan J.A., Heal W.P., Paape D., Barker K.V., Wilkinson A.J., Smith D.F., Leatherbarrow R.J., Tate E.W. (2015). Discovery of high affinity inhibitors of *Leishmania donovani* N-myristoyltransferase. MedChemComm.

[bib36] Rees-Channer R.R., Martin S.R., Green J.L., Bowyer P.W., Grainger M., Molloy J.E., Holder A. (2006). Dual acylation of the 45 kDa gliding-associated protein (GAP45) in *Plasmodium falciparum* merozoites. Mol. Biochem. Parasitol..

[bib37] Ritzefeld M., Wright M.H., Tate E.W. (2017). New developments in probing and targeting protein acylation in malaria, leishmaniasis and African sleeping sickness. Parasitology.

[bib38] Rosario V. (1981). Cloning of naturally occurring mixed infections of malaria parasites. Science.

[bib39] Schlott A.C., Holder A.A., Tate E.W. (2018). N-Myristoylation as a drug target in malaria: exploring the role of N-myristoyltransferase substrates in inhibitor mode of action. ACS Infect. Dis..

[bib40] Schrodinger (2014). The PyMOL Molecular Graphics System. Version 1.7.

[bib41] Slaughter A., Jurado K.A., Deng N., Feng L., Kessl J.J., Shkriabai N., Larue R.C., Fadel H.J., Patel P.A., Jena N. (2014). The mechanism of H171T resistance reveals the importance of Nδ-protonated His171 for the binding of allosteric inhibitor BI-D to HIV-1 integrase. Retrovirology.

[bib42] Smilkstein M., Sriwilaijaroen N., Kelly J.X., Wilairat P., Riscoe M. (2004). Simple and inexpensive fluorescence-based technique for high-throughput antimalarial drug screening. Antimicrob. Agents Chemother..

[bib43] Sutherland C.J., Tanomsing N., Nolder D., Oguike M., Jennison C., Pukrittayakamee S., Dolecek C., Hien T.T., do Rosário V.E., Arez A.P. (2010). Two nonrecombining sympatric forms of the human malaria parasite *Plasmodium ovale* occur globally. J. Infect. Dis..

[bib44] Tate E.W., Bell A.S., Rackham M.D., Wright M.H. (2013). N-Myristoyltransferase as a potential drug target in malaria and leishmaniasis. Parasitology.

[bib45] Thomas J.A., Collins C.R., Das S., Hackett F., Graindorge A., Bell D., Deu E., Blackman M.J. (2016). Development and application of a simple plaque assay for the human malaria parasite *Plasmodium falciparum*. PLoS One.

[bib46] Trager W., Jensen J.B. (2005). Human malaria parasites in continuous culture. J. Parasitol..

[bib47] Vagin A., Lebedev A. (2015). MoRDa, an automatic molecular replacement pipeline. Acta Crystallogr. Sect. A.

[bib48] Wagner J.C., Platt R.J., Goldfless S.J., Zhang F., Niles J.C. (2014). Efficient CRISPR-Cas9-mediated genome editing in *Plasmodium falciparum*. Nat. Methods.

[bib49] World Health Organization (2017). World Malaria Report 2017.

[bib50] Wright M.H., Clough B., Rackham M.D., Rangachari K., Brannigan J.A., Grainger M., Moss D.K., Bottrill A.R., Heal W.P., Broncel M. (2014). Validation of N-myristoyltransferase as an antimalarial drug target using an integrated chemical biology approach. Nat. Chem..

[bib51] Wright M.H., Paape D., Price H.P., Smith D.F., Tate E.W. (2016). Global profiling and inhibition of protein lipidation in vector and host stages of the sleeping sickness parasite *Trypanosoma brucei*. ACS Infect. Dis..

[bib52] Wright M.H., Paape D., Storck E.M., Serwa R.A., Smith D.F., Tate E.W. (2015). Global analysis of protein N-myristoylation and exploration of N-myristoyltransferase as a drug target in the neglected human pathogen *Leishmania donovani*. Chem. Biol..

[bib53] Wu Y., Gao F., Qi J., Bi Y., Fu L., Mohan S., Chen Y., Li X., Pinto B.M., Vavricka C.J. (2016). Resistance to mutant group 2 influenza virus neuraminidases of an oseltamivir-zanamivir hybrid inhibitor. J. Virol..

[bib54] Yeoh S., O'Donnell R.A., Koussis K., Dluzewski A.R., Ansell K.H., Osborne S.A., Hackett F., Withers-Martinez C., Mitchell G.H., Bannister L.H. (2007). Subcellular discharge of a serine protease mediates release of invasive malaria parasites from host erythrocytes. Cell.

[bib55] Yu Z., Brannigan J.A., Moss D.K., Brzozowski A.M., Wilkinson A.J., Holder A., Tate E.W., Leatherbarrow R.J. (2012). Design and synthesis of inhibitors of *Plasmodium falciparum* N-myristoyltransferase, a promising target for anti-malarial drug discovery. J. Med. Chem..

[bib56] Yu Z., Brannigan J.A., Rangachari K., Heal W.P., Wilkinson A.J., Holder A., Leatherbarrow R.J., Tate E.W. (2015). Discovery of pyridyl-based inhibitors of *Plasmodium falciparum* N-myristoyltransferase. MedChemComm.

[bib57] Zhang C., Xiao B., Jiang Y., Zhao Y., Li Z., Gao H., Ling Y., Wei J., Li S., Lu M. (2014). Efficient editing of malaria parasite genome using the CRISPR/Cas9 system. MBio.

