# Supplementary material for: Structure-Guided Identification of Resistance Breaking Antimalarial N‑Myristoyltransferase Inhibitors
Source: Cell Chem Biol. 2019 Jul 18;26(7):991–1000.e7. doi: 10.1016/j.chembiol.2019.03.015 (PMC6658617; doi:10.1016/j.chembiol.2019.03.015)
Supplement: Document S1. Figures S1–S6 and Tables S1–S6 [file mmc1.pdf]

Cell Chemical Biology, Volume *26*

Supplemental Information

Structure-Guided Identification of Resistance Breaking Antimalarial *N*-Myristoyltransferase Inhibitors

Anja C. Schlott, Stephen Mayclin, Alexandra R. Reers, Olivia Coburn-Flynn, Andrew S. Bell, Judith Green, Ellen Knuepfer, David Charter, Roger Bonnert, Brice Campo, Jeremy Burrows, Sally Lyons-Abbott, Bart L. Staker, Chun-Wa Chung, Peter J. Myler, David A. Fidock, Edward W. Tate, and Anthony A. Holder

Supplementary Information for

A novel antimalarial resistance mechanism leads to structure-guided identification of

resistance-breaking Plasmodium N-myristoyltransferase inhibitors

Author list in order of appearance on the paper:

Anja C. Schlott, <anja.schlott@crick.ac.uk> Francis Crick Institute, 1 Midland Rd, London NW1 1AT, UK; Molecular Sciences Research Hub, Imperial College, White City Campus Wood Lane, London W12 0BZ, UK, ORCID: 0000-0002-7139-8699

Stephen Mayclin <stephen.mayclin@ucb.com>, Seattle Structural Genomics Center for Infectious Disease (SSGCID), Seattle, WA 98109, - UCB Pharma, 7869 NE Day Road West, Bainbridge Island, WA 98110, ORCID: 0000-0002-1600-4554

Alexandra R. Reers, alexandra.reers@seattlechildrens.org, Seattle Structural Genomics Center for Infectious Disease (SSGCID); Center for Global Infectious Disease Research, Seattle Children's Research Institute, 307 Westlake Avenue North, Suite 500, Seattle, ORCID: 0000-0003-2561-6648

Olivia Coburn-Flynn <ocoburnflynn@gmail.com>, Department of Microbiology & Immunology, Columbia University Medical Center, New York, NY 10032

Andrew S. Bell<a.bell11@imperial.ac.uk>, Molecular Sciences Research Hub, Imperial College, White City Campus Wood Lane, London W12 0BZ, UK, ORCID: 0000-0002-0581-9387

Judith Green <judith.green@crick.ac.uk>, Francis Crick Institute, 1 Midland Rd, London NW1 1AT, UK, ORCID: 0000-0001-6825-9404

Ellen Knuepfer <Ellen.Knuepfer@crick.ac.uk>, Francis Crick Institute, 1 Midland Rd, London NW1 1AT, UK, ORCID: 0000-0002-6090-1877

David Charter <davidjcharter@gmail.com>, Structural and Biophysical Sciences, GlaxoSmithKline, Stevenage, Hertfordshire, UK

Roger Bonnert, rogerbonnert@aol.com, Medicines for Malaria Venture, Route de Pré-Bois 20, Post Box 1826, CH-1215 Geneva 15, Switzerland, ORCID: 0000-0002-2976-7374

Brice Campo, campob@mmv.org, Medicines for Malaria Venture, Route de Pré-Bois 20, Post Box 1826, CH-1215 Geneva 15, Switzerland, ORCID: 0000-0002-6425-5577

Jeremy Burrows, burrowsj@mmv.org, Medicines for Malaria Venture, Route de Pré-Bois 20, Post Box 1826, CH-1215 Geneva 15, Switzerland, ORCID: 0000-0001-8448-6068

*Sally Lyons-Abbott, saox@novonordisk.com, Seattle Structural Genomics Center for Infectious Disease (SSGCID); Center for Global Infectious Disease Research, Seattle Children's Research Institute, 307 Westlake Avenue North, Suite 500, Seattle

Bart L. Staker, bart.staker@seattlechildrens.org, Seattle Structural Genomics Center for Infectious Disease (SSGCID); Center for Global Infectious Disease Research, Seattle Children's Research Institute, 307 Westlake Avenue North, Suite 500, Seattle, ORCID: 0000-0001-9570-5086

Chun-Wa Chung, <chun-wa.h.chung@gsk.com>, Structural and Biophysical Sciences, GlaxoSmithKline, Stevenage, Hertfordshire, UK; Crick–GSK Biomedical LinkLabs, GSK Medicines Research Centre, Stevenage, UK, ORCID: 0000-0002-2480-3110

Peter J. Myler, peter.myler@seattlechildrens.org, Seattle Structural Genomics Center for Infectious Disease (SSGCID); Center for Global Infectious Disease Research, Seattle Children's Research Institute, 307 Westlake Avenue North, Suite 500, Seattle, Department of Biomedical Informatics & Medical Education and Department of Global Health, University of Washington, ORCID: 0000-0002-0056-0513

David A. Fidock, <df2260@cumc.columbia.edu>, Department of Microbiology & Immunology, Columbia University Medical Center, New York, NY 10032 USA and Division of Infectious Diseases, Department of Medicine, Columbia University Medical Center, New York, NY 10032, ORCID: 0000-0001-6753-8938

**Edward W Tate, <e.tate@imperial.ac.uk> Molecular Sciences Research Hub, Imperial College, White City Campus Wood Lane, London W12 0BZ, UK, ORCID: 0000-0003-2213-5814

Anthony A. Holder, <Tony.Holder@crick.ac.uk>, 1 Midland Rd, London NW1 1AT, UK, ORCID: 0000-0002-8490-6058

*Current address: Novo Nordisk Research Center, Seattle, WA 98109, USA

**Lead Contact: Tate, Edward W., e.tate@imperial.ac.uk

Corresponding Authors: Tate, Edward W., e.tate@imperial.ac.uk; and Holder, Anthony A., Tony.Holder@crick.ac.uk , and Anja C Schlott anja.schlott@crick.ac.uk

This PDF file includes:

Figure S1
Figure S2
Figure S3
Figure S4
Figure S5
Figure S6
Table S1
Table S2
Table S3
Table S4
Table S5
Table S6

Figures and legends SI Appendix

a)

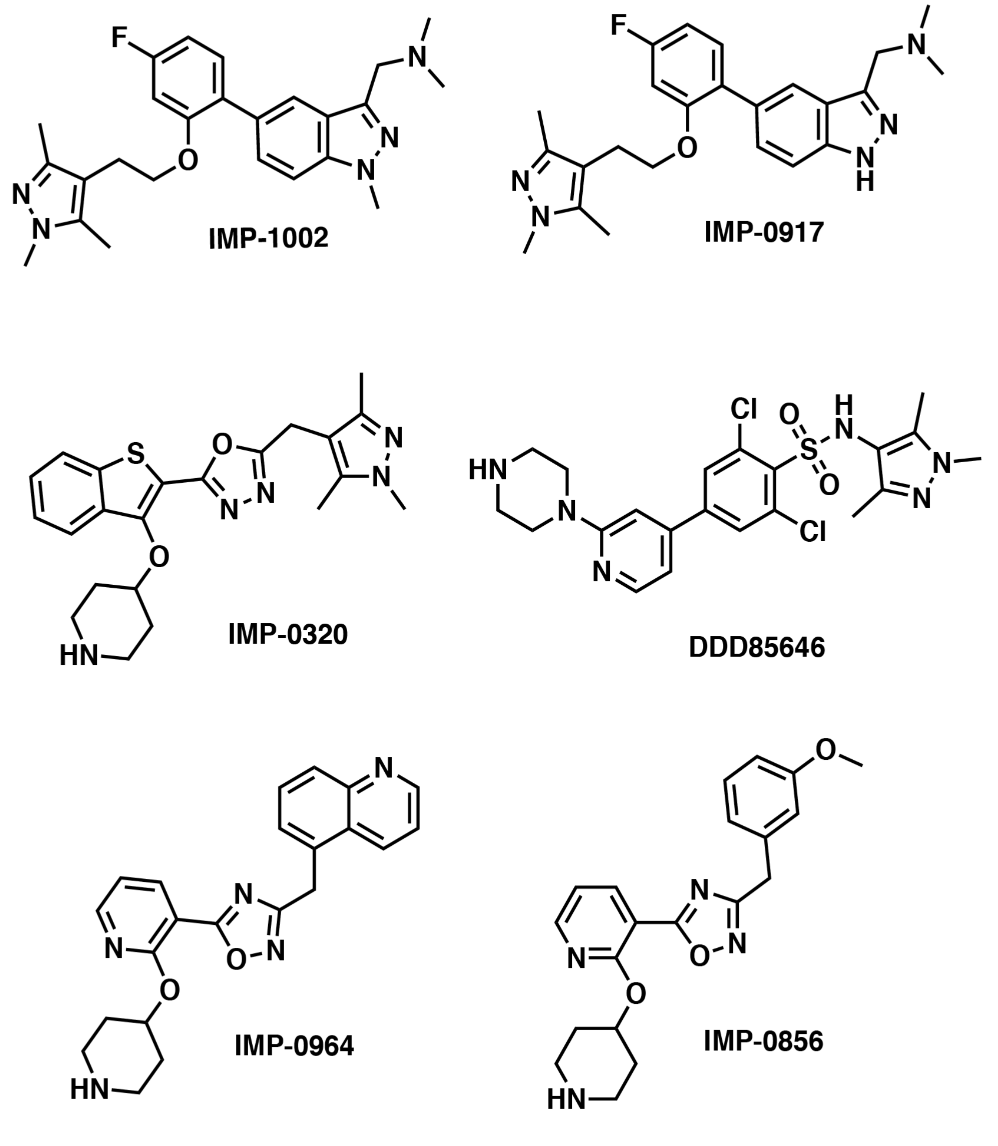

b)

NMTi name	Series name	Publication	DOI of paper	PDB # WT	PDB # G386E
IMP-1002	Series A			6MB1	6MB0
IMP-0917	Series A	Mousnier et al., 2018	10.1038/s41557-018-0039-2	5O6H	–
DDD8646	Series B	Wright et al. 2014	10.1038/nchem.1830	2YND	6MAZ
IMP-0320	Series C	Wright et al. 2014	10.1038/nchem.1830	2YNE	–
IMP-0964	Series D	Yu et al., 2015	10.1039/c5md00242g	–	–
IMP-0856	Series D	Yu et al., 2015	10.1039/c5md00242g	4UFX	–

Figure S1. a) Chemical structures of NMT inhibitors used in this study related to figure 1 and 6. b) Table of NMT inhibitors relating to their series name indicating closely related compounds, publication, and PDB # if a crystal structure is available.

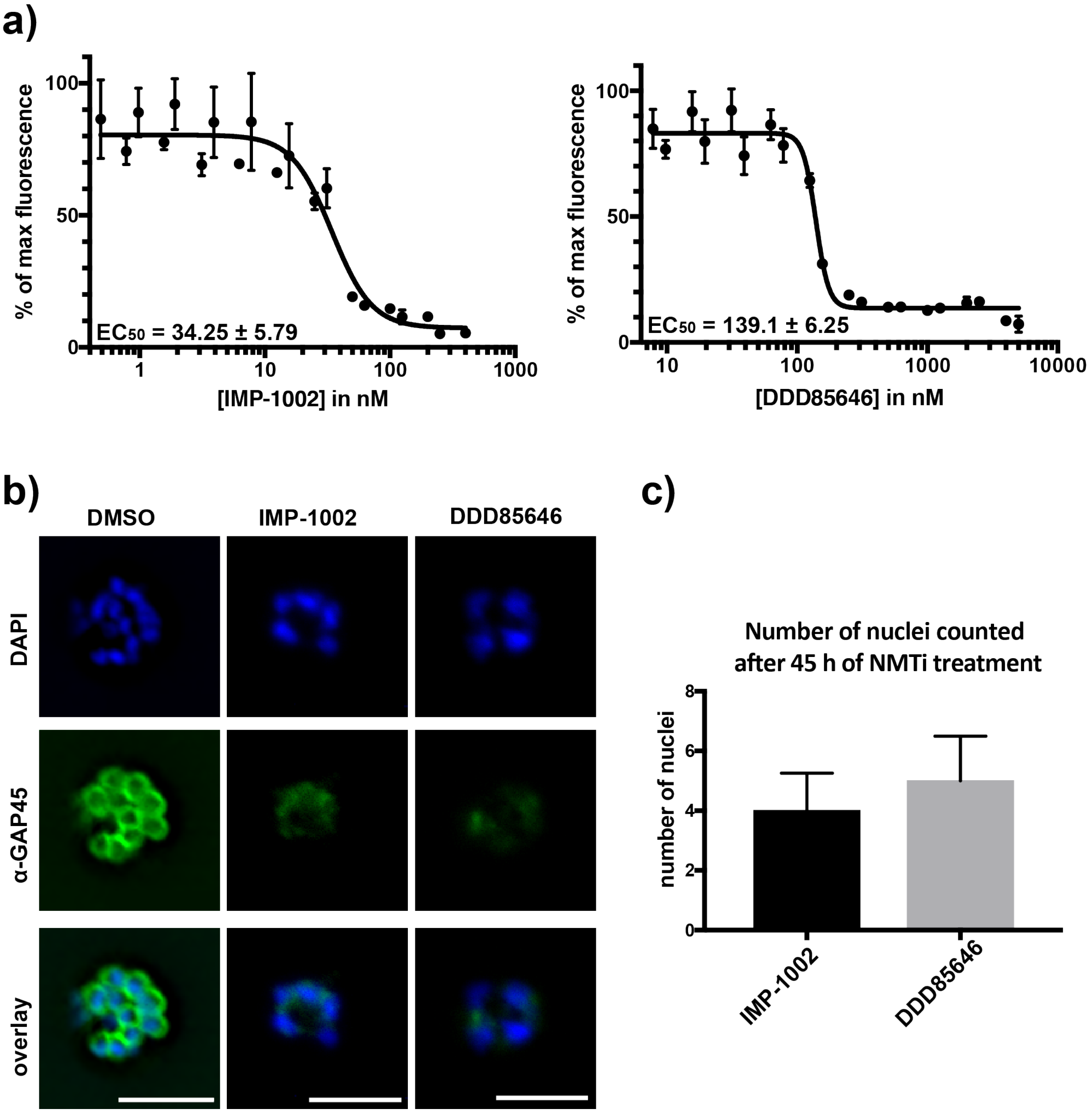

Figure S2. *P. falciparum* growth and development is inhibited by treatment with two NMT inhibitors related to figure 1 and 2.

a) *P. falciparum* growth, measured by fluorescence of SYBR Green bound to genomic DNA, in the presence of increasing concentrations of the NMT inhibitors. EC_{50} values for compounds IMP-1002 and DDD85646 are indicated on the respective graphs. b) Indirect immunofluorescence assay of parasites incubated from the ring stage with a control (DMSO)

or four times the EC_{50} concentration of each NMT inhibitor (1 µM DDD85646 and 140 nM IMP1002) and examined at 45 hours post invasion (h PI): nuclei stained by DAPI (blue) and GAP45 localization in green. GAP45 is a well-known NMT substrate and an indicator of the Inner membrane complex (IMC). The number of nuclei in each cell and the abundance and location of GAP45 were affected by drug treatment. All images were acquired with a Nikon PlanApo 100x/1.45 oil immersion objective. Scale bar = 5 µm. c) Number of nuclei counted at 45 h PI for parasites treated with IMP-1002 or DDD85646; error bars equal standard deviation (SD) of the median.

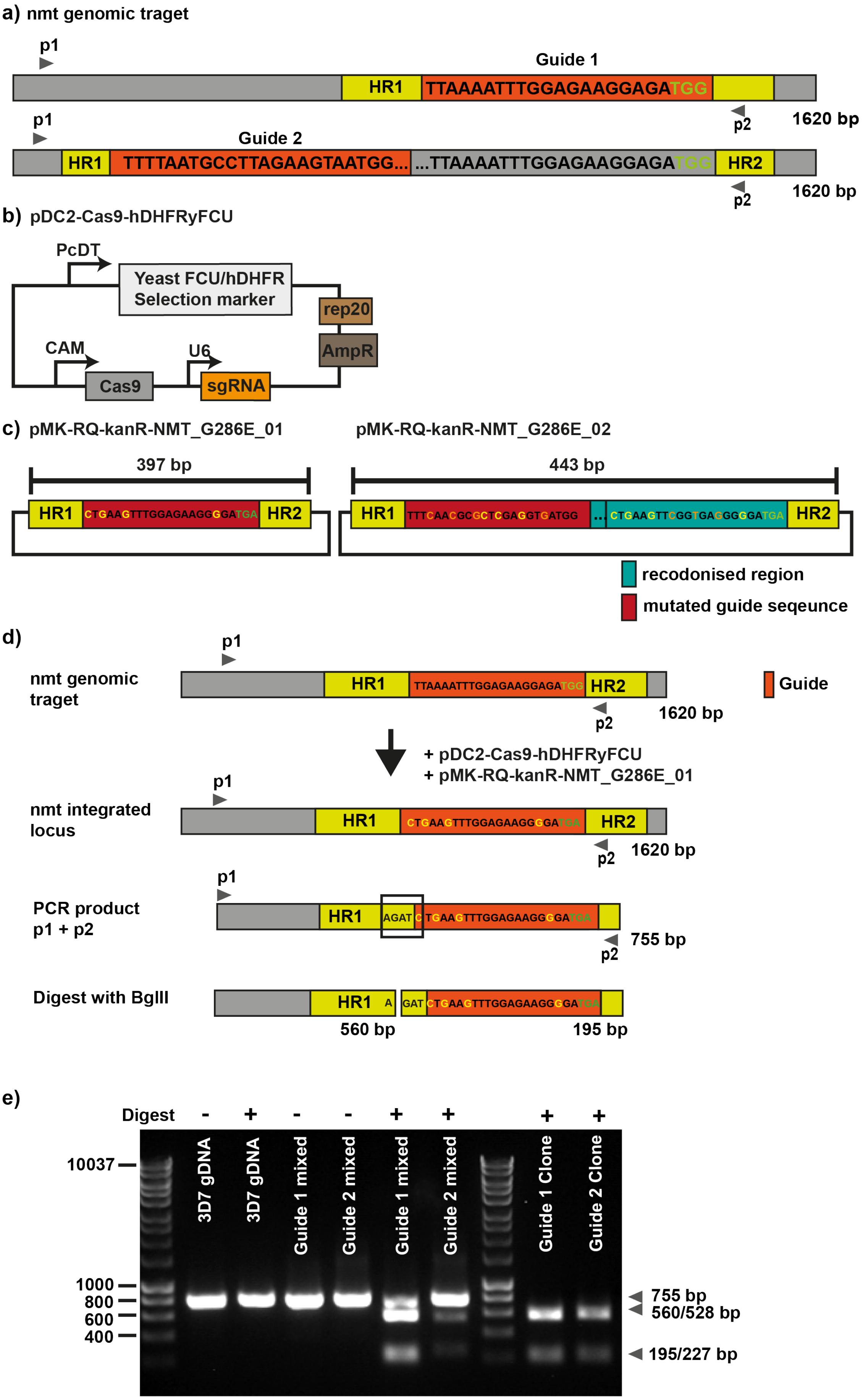

a) nmt genomic traget
p1
Guide 1
HR1
TTAAAATTTGGAGAAGGAGATGG
p2
1620 bp
p1
Guide 2
HR1
TTTTAATGCCTTAGAAGTAATGG...
...TTAAAATTTGGAGAAGGAGATGG
HR2
p2
1620 bp
b) pDC2-Cas9-hDHFRyFCU
PcDT
Yeast FCU/hDHFR Selection marker
rep20
AmpR
CAM
U6
Cas9
sgRNA
c) pMK-RQ-kanR-NMT_G286E_01
pMK-RQ-kanR-NMT_G286E_02
397 bp
443 bp
HR1
CTGAAGTTTGGAGAAGGGGATGA
HR2
HR1
TTTCAACGCGCTCGAGGTGATGG
...
CTGAAGTTCGGTGAGGGGGATGA
HR2
recodonised region
mutated guide seqeunce
d)
nmt genomic traget
p1
HR1
TTAAAATTTGGAGAAGGAGATGG
HR2
p2
1620 bp
Guide
+ pDC2-Cas9-hDHFRyFCU
+ pMK-RQ-kanR-NMT_G286E_01
nmt integrated locus
p1
HR1
CTGAAGTTTGGAGAAGGGGATGA
HR2
p2
1620 bp
PCR product p1 + p2
p1
HR1
AGATCTGAAGTTTGGAGAAGGGGATGA
p2
755 bp
Digest with BglII
HR1
A
GATCTGAAGTTTGGAGAAGGGGATGA
560 bp
195 bp
e)
Digest
- + - - + + + +
3D7 gDNA
3D7 gDNA
Guide 1 mixed
Guide 2 mixed
Guide 1 mixed
Guide 2 mixed
Guide 1 Clone
Guide 2 Clone
10037
1000
800
600
400
755 bp
560/528 bp
195/227 bp

Figure S3. Generation of *P. falciparum* parasite lines containing the G386E mutation in the *nmt* gene related to figure 2 and 3.

a) Schematic of WT *nmt* ORF highlighting the two guide sequences and sequence before the desired mutation codon TGG highlighted in green. The PAM sequence of guide 1 was destroyed and mutated to yield the desired G386E mutation. For Guide 2 that lies upstream of the introduced mutation, a stretch of 45 bp was recodonised. b) Schematic of the Cas9/guide RNA plasmid. c) Schematic of the rescue plasmids with the modified open reading frame (OFR) containing the G386E point mutation (TGA) in green. Homology regions (HRs) on rescue plasmids used for targeting were 200 bp (HR1) and 169/174 bp (HR2) long. The two mutated guide sequences are shown in red. Silent point mutations for the generation of unique restriction sites in the integrated construct are highlighted in yellow. Additional silent mutations are highlighted in orange. d) Screening by PCR and restriction enzyme digest to distinguish WT from integrated parasites. Only integration of G286E_01 is shown. The restriction site is highlighted by a box. e) Agarose gel staining for DNA showing restriction digest of DNA amplified from parental line (3D7), mixed population for both transfections and the two clones used for the analysis from two independent transfections.

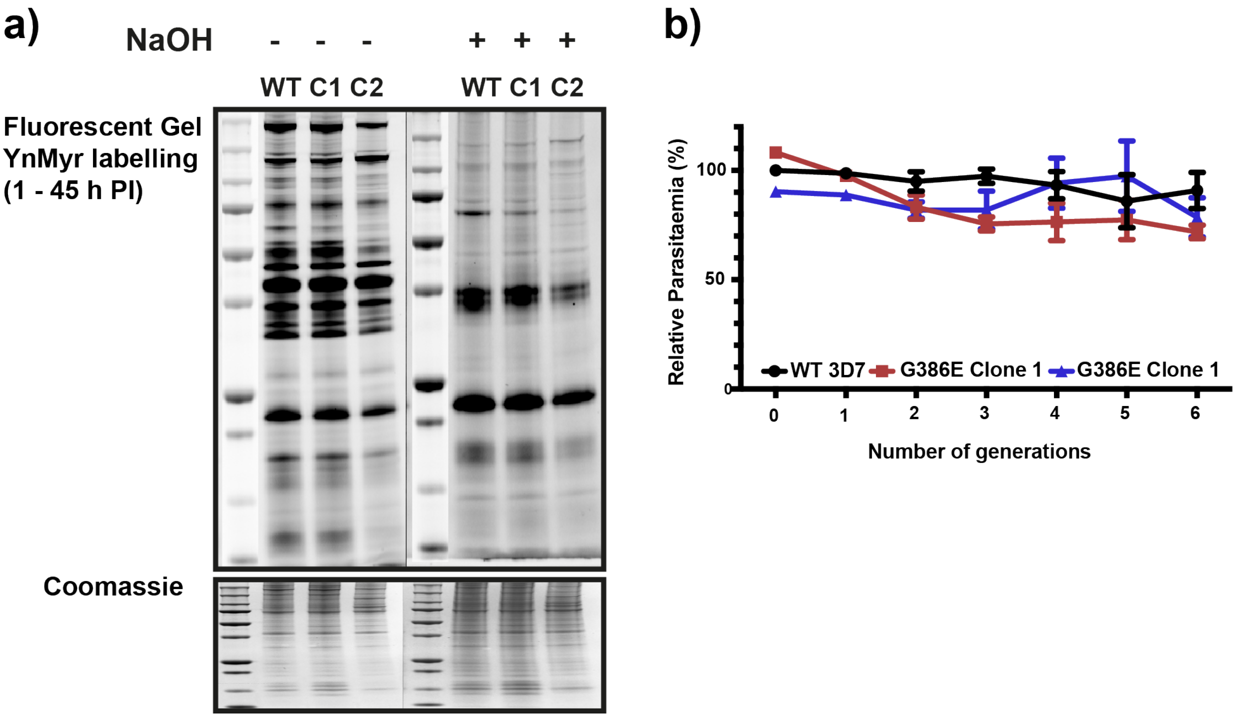

Figure S4. *P. falciparum* G386E parasites do not show a phenotype and show normal myristoylation patterns related to figure 3.

a) YnMyr labelling of *P. falciparum* 3D7 G386E and WT parasites during the entire erythrocyte life cycle. YnMyr labelling was subjected to NaOH treatment to hydrolyse the base-labile incorporation of YnMyr into GPI-anchored proteins. Coomassie stain indicates loading for each lane. b) Growth assay of *P. falciparum* 3D7 WT and G386E parasites over six generations. Starting parasitemia was determined by flow cytometry (Hoechst stain). WT parasitemia was determined after each cycle and a dilution factor calculated to give 0.8% parasitemia. All three lines were diluted using this same factor to avoid cultures overgrowing. Parasitemias were measured after each cycle using flow cytometry. Total parasitemia was normalized to the DMSO control replicate with the highest value. N = 3 biological replicates with 3 technical replicas each. Error bars equal SD. Multiple t-tests were performed for each generation (two-stage linear step-up procedure of Benjamini, Krieger and Yekutieli, with false-discovery

rate = 1 %). There was no significant difference in relative parasitemia between the three lines.

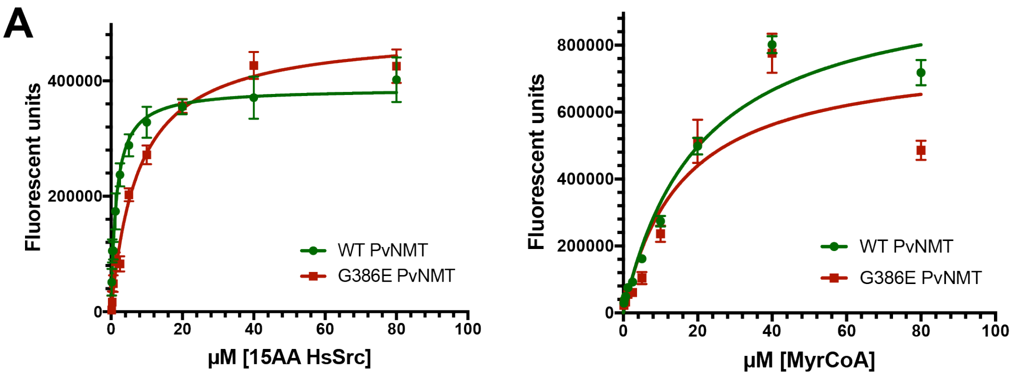

	15AA peptide substrate				MyrCoA	
	PvNMT WT		PvNMT[G386E]		PvNMT WT	PvNMT[G386E]
Biological replicates	1	2	1	2	1	1
Km (µM)	1.5	1.2	7.7	8.2	4.1	1.1
Vmax (FU/sec)	6446	5603	8096	8309	28568	25000
Kcat	19323	16795	24268	24907	90008	78769
Vmax/Km	4353	4661	1058	1008	6915	22666

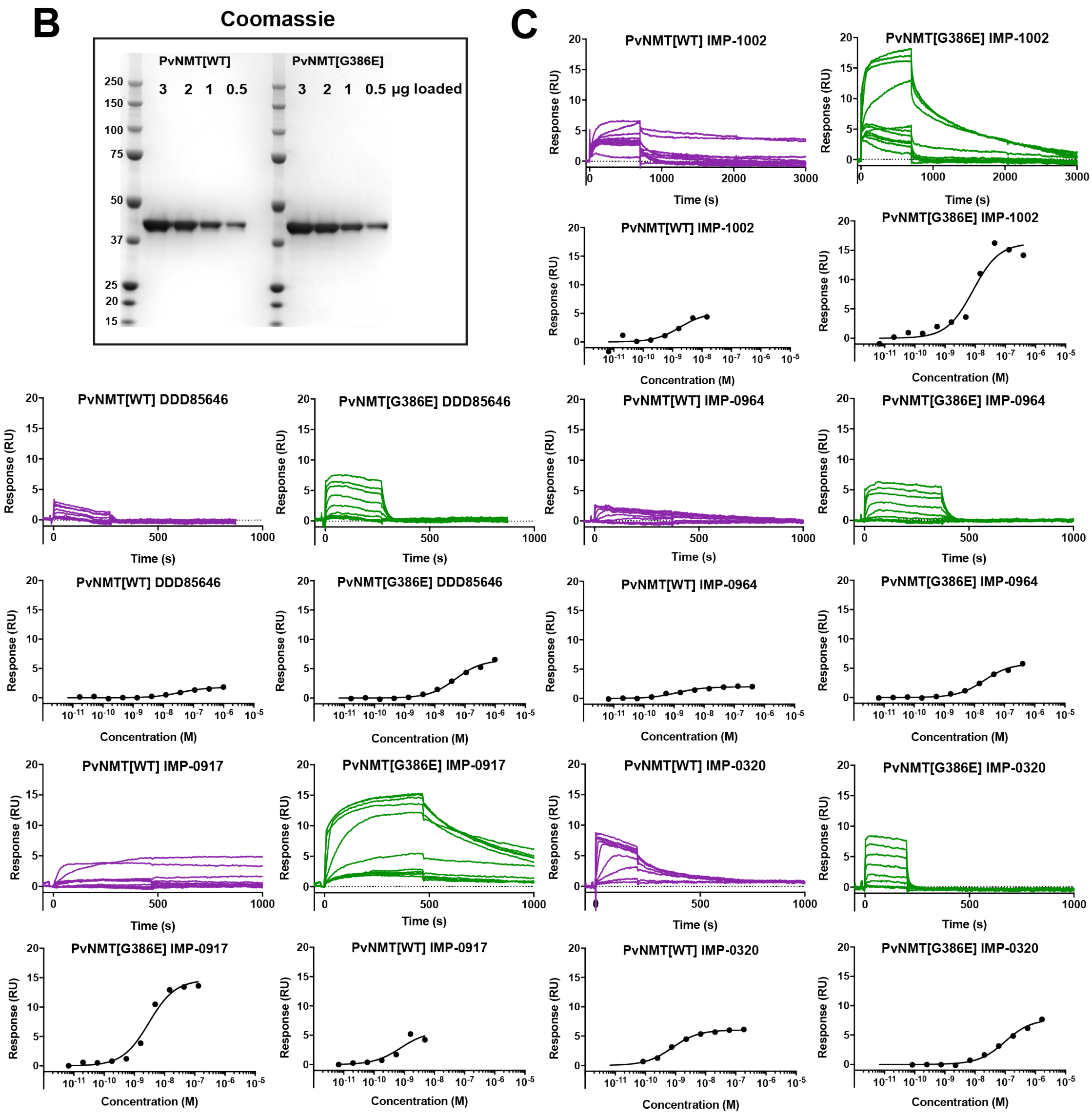

Figure S5. SPR equilibrium analysis of recombinant PvNMT[WT] and [G386E] variant related to figure 4 and 6.

A) Michaelis-Menten Plot for the KM Vmax determination for the myristoyl-CoA substrate and the 15-residue HsSrc peptide. PvNMT[WT] is in green and PvNMT[G386E] is in red. Table of KM, Vmax, Kcat and the catalytic efficiency (Vmax/KM) for the myrCoA and peptide kinetics. (n = 2 for peptide, n = 1 for MyrCoA). B) Coomassie of purified protein showing an indication of the purity of the sample. C) Surface plasmon resonance analysis of the interaction of NMTi with PvNMT[WT] and PvNMT[G386E]. The relationship between each point in the concentration series and the report points from sensorgrams measured for each of the NMTi compounds was fitted to a 1:1 steady state affinity model to calculate the equilibrium dissociation constant (K_D)

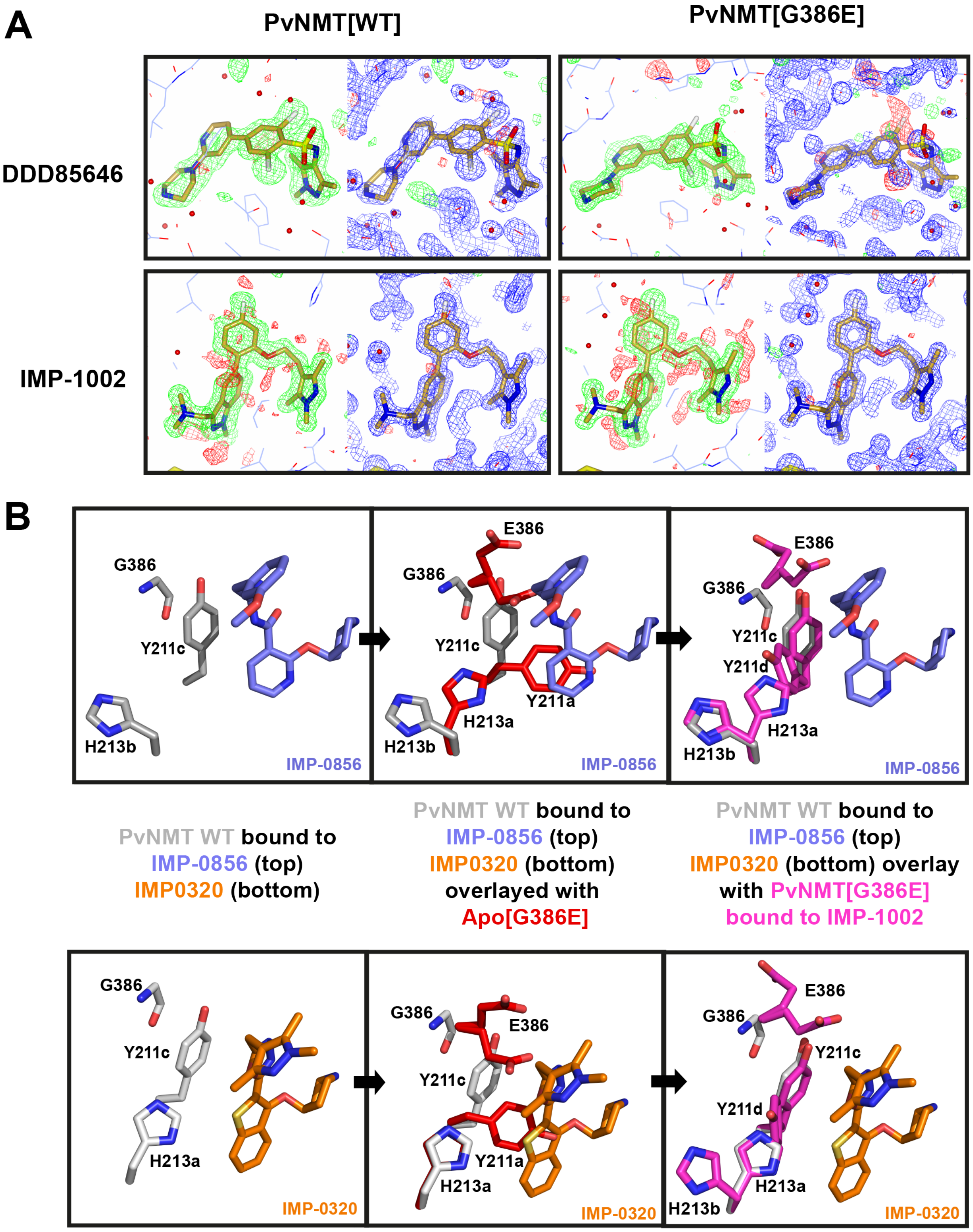

Figure S6: Crystallographic analysis of NMT inhibitor interaction with PvNMT[WT] and PvNMT[G386E] related to figure 5. A) Omit map for DDD85646 and IMP-1002 bound to PvNMT[WT] and PvNMT[G386E]. For each panel, FO-FC (contoured at 3.0 sigma) density for

fully refined structure with inhibitor atoms removed is shown on the left. For clarity, inhibitor stick representation is overlaid. For each panel, on the right is shown 2FO-FC (contoured at 1.0 sigma) and FO-FC (contoured at 3.0 sigma) density from fully refined structure including inhibitor atoms. B) Movement of residues as a result of the G386E mutation upon binding of two different NMT inhibitors: IMP-0856 at the top (PDB ID: 4UFX) and IMP-0320 (PDB ID: 2YNE) at the bottom. First image shows the PvNMT[WT] with respective inhibitor bound. Upon overlay with Apo[G386E] it becomes apparent that Y211 can no longer position in the Y211c rotamer because of the G386E substitution, and the Y211a rotamer cannot be adopted with IMP-0320 or IMP-0856 bound. Therefore, in the last image we predict that a PvNMT[G386E] bound to the respective NMTi would have similar characteristics as PvNMT[G386E] bound to IMP-1002 (PDB: 6MB0) with movement of Y211 from Y211c to Y211d.

	G386E IMP1002	WT IMP1002	G386E IMP0366	G386E (apo)
**Wavelength**	0.97872	0.97872	0.97872	0.97872
**Resolution range**	50 - 1.55<br>(1.59 - 1.55)	50 - 1.5<br>(1.54 - 1.50)	50 - 1.55<br>(1.59 - 1.55)	50 - 2.05<br>(2.10 - 2.05)
**Space group**	P 21 21 21	P 21 21 21	P 21 21 21	P 21 21 21
**Unit cell**	57.44 121.19<br>178.25 90 90 90	57.56 119.11<br>176.08 90 90 90	57.5 121.25<br>177.93 90 90 90	57.81 120.81<br>179.06 90 90 90
**Total reflections**	1064299 (76554)	1120216 (63971)	1116596 (81282)	491256 (36093)
**Unique reflections**	176323 (12704)	193711 (14201)	180669 (13216)	79557 (5777)
**Multiplicity**	6.0 (6.0)	5.8 (4.5)	6.2 (6.2)	6.2 (6.2)
**Completeness (%)**	97.6 (96.2)	99.9 (99.9)	100.0 (100.0)	99.9 (100.0)
**Mean I/sigma(I)**	12.78 (3.18)	11.91 (2.62)	18.81 (3.40)	15.01 (3.15)
**Wilson B-factor**	11.32	12.23	12.57	23.55
**R-merge**	0.094 (0.544)	0.090 (0.462)	0.066 (0.537)	0.096 (0.572)
**R-meas**	0.102 (0.595)	0.099 (0.523)	0.073 (0.586)	0.105 (0.624)
**CC1/2**	0.997 (0.838)	0.997 (0.822)	0.999 (0.887)	99.8 (0.875)
**Reflections used in refinement**	176228 (17160)	193683 (19198)	180657 (17875)	79549 (7843)
**Reflections used for R-free**	1978 (196)	2060 (195)	2028 (207)	2024 (183)
**R-work**	0.1526 (0.2258)	0.1549 (0.2129)	0.1450 (0.1861)	0.1571 (0.1907)
**R-free**	0.1780 (0.2812)	0.1839 (0.2363)	0.1657 (0.2327)	0.2178 (0.2542)
**Number of non-hydrogen atoms**	11918	11841	11941	10882
**macromolecules**	9752	9687	9707	9462
**ligands**	314	325	314	230
**solvent**	1852	1829	1920	1190
**Protein residues**	1144	1144	1143	1136
**RMS(bonds)**	0.006	0.005	0.006	0.007
**RMS(angles)**	0.82	0.79	0.84	0.88
**Ramachandran favored (%)**	97.8	97.71	97.71	97.07
**Ramachandran allowed (%)**	2.2	2.29	2.29	2.84
**Ramachandran outliers (%)**	0	0	0	0.09
**Rotamer outliers (%)**	0.55	0.55	0.28	0.58
**Clashscore**	1.55	0.9	2.56	1.5
**Average B-factor**	16.06	17.1	17.82	29.01
**macromolecules**	13.85	14.98	15.32	27.7
**ligands**	11.87	14.28	19.58	48.67
**solvent**	28.38	28.85	30.16	35.6
**Number of TLS groups**	16	17	22	9

Table S1: Data collection and refinement statistics of crystallographic data related to figure 5.

Growth assay (EC_{50} determination)

	PfNMT[WT]			PfNMT[G398E] Clone 1			PfNMT[G398E] Clone 2			Ratio PfNMT[G386] / PfNMT[WT]	
	EC_{50} (nM)	95% CI (nM)	Hill Slope	EC_{50} (nM)	95% CI (nM)	Hill Slope	EC_{50} (nM)	95% CI (nM)	Hill Slope	Clone 1	Clone 2
**IMP-1002**	9.6	8.2 - 11	-2.3	230	190 - 280	-1.7	201	170 - 250	-1.5	24	21
**DDD85646**	69	51 - 93	-0.65	91	75 - 110	-1.4	103	86 - 120	-1.5	1.3	1.5
**IMP-0964**	93	83 - 103	-2.9	1080	913 - 1300	-1.3	947	78 - 83	-0.89	12	10
**IMP-0320**	80	68 - 93	-2.6	1500	1200 - 1800	-1.4	1400	1100 - 1900	-0.91	19	18

Table S2. Raw data for SYBR Green growth assay related to figure 4 and 6. SYBR Green growth assay data to determine the EC_{50} of [WT] and [G386E] parasites with two biological replicates in the form of two clones from independent transfections with different guides. All numbers are given to two significant figures. In the last column the ratio is calculated of the EC_{50} of [G386E]/[WT].

CPM enzyme assay (IC_{50} determination)

		PvNMT[WT]			PvNMT[G398E]			
	**Replicate**	**IC_{50} (nM)**	**95% CI (nM)**	**Hill Slope**	**IC_{50} (nM)**	**95% CI (nM)**	**Hill Slope**	**Ratio PvNMT[G386E]/ PvNMT[WT]**
**IMP-1002**	**1**	1.9	1.6 - 2.1	-2.1	6.0	5.4 - 6.5	-1.8	3.1
	**2**	2.1	2.0 - 2.3	-1.7	7.1	2.3 - 41	-0.82	3.4
	**3**	4.9	4.4 - 5.4	-2.0	6.9	6.2 - 7.7	-1.5	1.4
**DDD85646**	**1**	53	38 - 65	-0.9	55	41 - 62	-0.9	1.0
	**2**	47	37 - 66	-1.1	77	61 - 110	-1.5	1.6
	**3**	48	40 - 62	-1.1	80	52 - 204	-0.9	1.7
**IMP-0964**	**1**	3.7	3.3 - 4.2	-1.2	21	18 - 24	-1.2	5.5
	**2**	4.7	2.3 - 7.2	-3.6	17	12 - 24	-1.3	3.6
	**3**	12	12 - 13	-1.9	35	33 - 38	-1.5	2.9
**IMP-0320**	**1**	10	9.2 - 11	-1.2	380	180 - 4900	-0.9	38
	**2**	9.1	8.5 - 10	-1.3	340	140 - 1800	-0.9	37
	**3**	10	9.4 - 11	-1.3	300	150 - 6800	-1.0	29

Table S3. Raw data for CPM enzyme assay related to figure 4 and 6. CPM enzyme assay data to determine the IC_{50} of PvNMT[WT] and PvNMT[G386E] with three biological replicates of four different NMTi compounds. All numbers are given to two significant figures. The Hill Slope of the IC_{50} gives an indication how steep the curve is. For very high hill slopes < -1, the IC_{50} value cannot be accurately determine and is presumably lower than indicated. In the last column the ratio is calculated of the IC_{50} of PvNMT[G386E]/PvNMT[WT].

a)

All Tm in °C	Tm	Tm	ΔTm	ΔTm	ΔΔTm
**Protein**					PvNMT[WT] -
- **MyrCoA**	PvNMT[WT]	PvNMT[G386E]	PvNMT[WT]	PvNMT[G386E]	PvNMT[G386E]
**Compound ↓**					
1% DMSO reference	64.6 ± 0.6	61.0 ± 0.7	0	0	0
IMP-0320	>70.4 #	61.7 ± 0.8	>5.7	0.7	>5.0
DDD85646	69.0 ± 0.1	62.4 ± 0.7	4.4	1.3	3
IMP-1002	>73.4 #	62.8 ± 0.7	>8.8	1.8	>7.0

Protein was not fully denatured at the end of the thermal ramp, therefore these values represent the lower estimates of Tm

b)

All Tm in °C	Tm	Tm	ΔTm	ΔTm	ΔΔTm
**Protein**					PvNMT[WT] -
**+ 4μM MyrCoA**	PvNMT[WT]	PvNMT[G386E]	PvNMT[WT]	PvNMT[G386E]	PvNMT[G386E]
**Compound ↓**					
1% DMSO reference	67.8 ± 0.6	61.6 ± 0.5	0	0	0
IMP-0320	>73 #	63.2 ± 2.2	>5.2	1.6	>3.6
DDD85646	71.0 ± 0.1	63.0 ± 0.3	3.2	1.4	1.8
IMP-1002	>75 #	66.6 ± 0.6	>7.2	5	>2.2

Protein was not fully denatured at the end of the thermal ramp, therefore these values represent the lower estimates of Tm.

Table S4: Thermal shift assay data related to figure 4. The degree of stabilisation of PvNMT[WT] and PvNMT[G386E] was determined using at least two experimental replicates with two technical replicates (n>5) with three different NMTi compounds in the absence (a) and (b) presence of (myrCoA). All Tm values are given in °C and ΔTm is determined by subtracting the Tm for the DMSO control to determine the compound induced stabilisation each protein. There is significantly greater stabilisation of the PvNMT [WT] for compounds such as IMP-0320 and IMP-1002 over PvNMT [G386E], as evident from the ΔΔTm values. For the PvNMT [WT], where the Tm of the compound bound protein exceeds the top of the temperature range for the instrument, a greater than value for the Tm is given. These lower estimates of Tm may lead to compression of the difference window between PvNMT [WT] and PvNMT [G386E] for IMP-0320 and IMP-1002. This is most noticeable with the higher baseline Tm of PvNMT [WT] in the presence of myrCoA.

	HsNMT1	HsNMT2	PvNMT	HsNMT1/PvNMT	HsNMT2/PvNMT
**IMP-1002**	< 0.0072 ×/÷ 3.7821 (n=4)	0.0119 ×/÷ 2.3488 (n=3)	< 0.0031 ×/÷ 2.5220 (n=7)	2.32	3.84
**DDD85646**	0.0127 ×/÷ 1.4054 (n=3)	0.0200 (n=1)	0.0407 ×/÷ 1.6927 (n=5)	0.3	0.5

Table S5. IC_{50} of HsNMT1 HsNMT2 and PvNMT with IMP-1002 and DDD85646 determined through CPM assay related to figure 1. Values are concentrations in µM. Values are the geometric mean with associated standard error showing the variation from the biological replicates. The last two columns highlight the fold difference in IC_{50} of PvNMT compared to HsNMT1 and HsNMT2. While there is a 2-3 fold shift in selectivity towards PvNMT with IMP-1002 this selectivity is diminished for DDD85646.

a)

Compound	PvNMT [WT] KD (expt1)	PvNMT [WT] (expt2)	PvNMT [WT] (expt3)	PvNMT [WT] (expt4)
**IMP-0320**	7.79E-10	2.41E-09	7.47E-09	6.87E-10
**DDD85646**	3.61E-08	2.96E-08	1.51E-08	2.18E-08
**IMP-0964**	1.29E-09	6.47E-10	8.17E-09	3.66E-10
**IMP-1002**	1.80E-09			
**IMP-0917**	6.48E-10	7.52E-10		

b)

Compound	PvNMT[G386E] KD (expt1)	PvNMT[G386E] (expt2)	PvNMT[G386E] (expt3)	PvNMT[G386E] (expt4)
**IMP-0320**	9.72E-08	1.88E-07	1.68E-07	9.95E-08
**DDD85646**	4.90E-08	4.47E-08	2.23E-08	4.91E-08
**IMP-0964**	2.07E-08	1.64E-08	1.52E-08	1.22E-08
**IMP-1002**	8.37E-09	4.83E-09	3.63E-09	2.98E-09
**IMP-0917**	2.58E-09	3.29E-09		

c)

Compound	PvNMT[G386E]/ PvNMT[WT] KD (expt1)	PvNMT[G386E]/ PvNMT[WT] (expt2)	PvNMT[G386E]/ PvNMT[WT] (expt3)	PvNMT[G386E]/ PvNMT[WT] (expt4)
**IMP-0320**	77.8	22.5	124.8	144.9
**DDD85646**	1.5	1.5	2.3	1.4
**IMP-0964**	25.3	41.5	16.1	11.7
**IMP-1002**	4.7			
**IMP-0917**	4	4.4		

d)

Compound	K_D (PvNMT[G86E]) / K_D PvNMT[WT])
IMP-0320	92.5 ±54.5
DDD85646	1.6 ±0.4
IMP-0964	19.9 ±14.2
IMP-1002	4.7 (n =1)
IMP-0917	4.2 ±0.3

Table S6. Fold difference of equilibrium dissociation constants (KD) determined from SPR analyses for the interaction between each NMTi compound with PvNMT[WT] or PvNMT[G386E] related to figure 4 and 6. a) Raw data of K_D values for PvNMT[WT] (n = 1-4) and b) PvNMT[G386E] (n = 2-4). c) The SPR experiment was configured so that the same concentration titrations were flowed over surfaces immobilized with the three proteins. To focus on the selectivity between proteins and remove inter experiment variability, the ratio of KD's between the three proteins was calculated in each experiment (n = 1-4) as this allows better differentiate between differences in affinity. d) The K_D of each compound with either

PvNMT[G386E] was divided by the K_D with PvNMT[WT] to calculate fold difference. The standard deviation of the mean fold difference is stated.
