# Supplementary material for: Structure-Guided Identification of Resistance Breaking Antimalarial N‑Myristoyltransferase Inhibitors
Source: Cell Chem Biol. 2019 Jul 18;26(7):991–1000.e7. doi: 10.1016/j.chembiol.2019.03.015 (PMC6658617; doi:10.1016/j.chembiol.2019.03.015)
Supplement: Document S2. Article plus Supplemental Information [file mmc2.pdf]

Article

Cell Chemical Biology

# Structure-Guided Identification of Resistance Breaking Antimalarial *N*-Myristoyltransferase Inhibitors

## Graphical Abstract

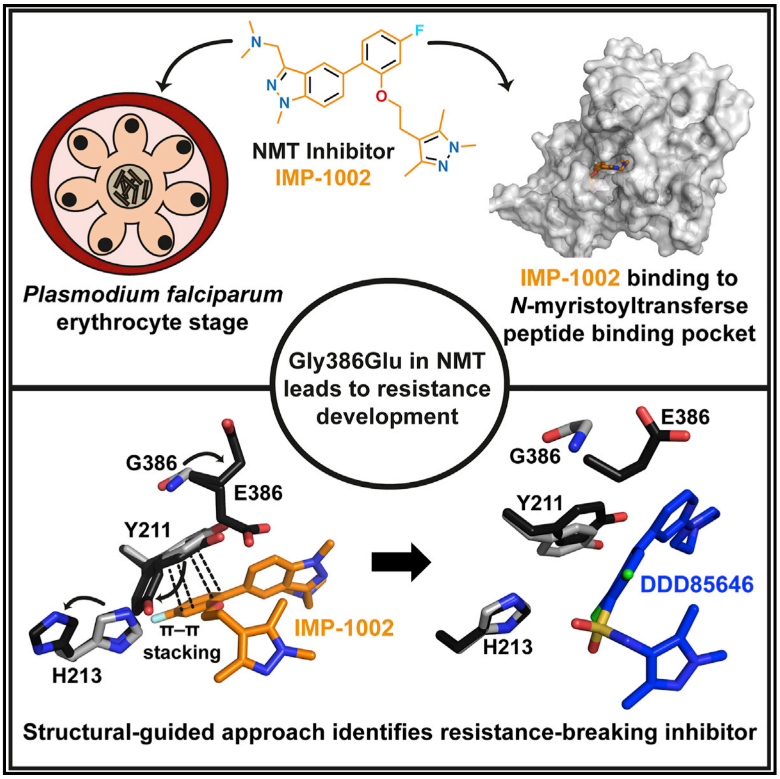


## Authors

Anja C. Schlott, Stephen Mayclin, Alexandra R. Reers, ..., David A. Fidock, Edward W. Tate, Anthony A. Holder

## Correspondence

anja.schlott@crick.ac.uk (A.C.S.),
e.tate@imperial.ac.uk (E.W.T.),
tony.holder@crick.ac.uk (A.A.H.)

## In Brief

Structural studies of N-myristoyltransferase (NMT) of the malaria parasite *Plasmodium falciparum* combined with inhibitors decipher how a point mutation in *nmt* leads to the development of resistance against an inhibitor series, and enables identification of an NMT inhibitor that can overcome this resistance phenotype.

## Highlights

- Discovery of a mutant offering resistance against *P. falciparum* NMT inhibitor IMP-1002
- Structural basis of the mechanism of resistance revealed by X-ray crystallography
- Genetic and chemical validation of resistance using CRISPR-Cas9 and enzyme assays
- Crystal structures of PvNMT[G386E] with IMP-1002 versus DDD85646, overcoming resistance

Schlott et al., 2019, Cell Chemical Biology *26*, 991–1000
July 18, 2019 © 2019 The Authors. Published by Elsevier Ltd.
https://doi.org/10.1016/j.chembiol.2019.03.015

CellPress

Cell Chemical Biology
Article

# Structure-Guided Identification of Resistance Breaking Antimalarial *N*-Myristoyltransferase Inhibitors

Anja C. Schlott,[1,2,*] Stephen Mayclin,[3,4] Alexandra R. Reers,[3,5] Olivia Coburn-Flynn,[6] Andrew S. Bell,[2] Judith Green,[1] Ellen Knuepfer,[1] David Charter,[7] Roger Bonnert,[8] Brice Campo,[8] Jeremy Burrows,[8] Sally Lyons-Abbott,[3,5,13] Bart L. Staker,[3,5] Chun-Wa Chung,[7,9] Peter J. Myler,[3,5,10,11] David A. Fidock,[6,12] Edward W. Tate,[2,14,*] and Anthony A. Holder[1,*]
[1]Francis Crick Institute, 1 Midland Road, London NW1 1AT, UK
[2]Molecular Sciences Research Hub, Imperial College, White City Campus Wood Lane, London W12 0BZ, UK
[3]Seattle Structural Genomics Center for Infectious Disease (SSGCID), Seattle, WA 98109, USA
[4]UCB Pharma, 7869 NE Day Road West, Bainbridge Island, WA 98110, USA
[5]Center for Global Infectious Disease Research, Seattle Children's Research Institute, 307 Westlake Avenue North, Suite 500, Seattle, USA
[6]Department of Microbiology & Immunology, Columbia University Medical Center, New York, NY 10032, USA
[7]Structural and Biophysical Sciences, GlaxoSmithKline, Stevenage, Hertfordshire, UK
[8]Medicines for Malaria Venture, Route de Pré-Bois 20, Post Box 1826, 1215 Geneva 15, Switzerland
[9]Crick–GSK Biomedical LinkLabs, GSK Medicines Research Centre, Stevenage, UK
[10]Department of Biomedical Informatics & Medical Education, University of Washington, Seattle, USA
[11]Department of Global Health, University of Washington, Seattle, USA
[12]Division of Infectious Diseases, Department of Medicine, Columbia University Medical Center, New York, NY 10032, USA
[13]Present address: Novo Nordisk Research Center, Seattle, WA 98109, USA
[14]Lead Contact
*Correspondence: anja.schlott@crick.ac.uk (A.C.S.), e.tate@imperial.ac.uk (E.W.T.), tony.holder@crick.ac.uk (A.A.H.)
https://doi.org/10.1016/j.chembiol.2019.03.015

## SUMMARY

The attachment of myristate to the N-terminal glycine of certain proteins is largely a co-translational modification catalyzed by N-myristoyltransferase (NMT), and involved in protein membrane-localization. Pathogen NMT is a validated therapeutic target in numerous infectious diseases including malaria. In *Plasmodium falciparum*, NMT substrates are important in essential processes including parasite gliding motility and host cell invasion. Here, we generated parasites resistant to a particular NMT inhibitor series and show that resistance in an *in vitro* parasite growth assay is mediated by a single amino acid substitution in the NMT substrate-binding pocket. The basis of resistance was validated and analyzed with a structure-guided approach using crystallography, in combination with enzyme activity, stability, and surface plasmon resonance assays, allowing identification of another inhibitor series unaffected by this substitution. We suggest that resistance studies incorporated early in the drug development process help selection of drug combinations to impede rapid evolution of parasite resistance.

## INTRODUCTION

Malaria, caused by parasitic protozoa of the genus *Plasmodium*, led to an estimated 216 million clinical cases and nearly half a million deaths in 2016 (World Health Organization, 2017). In humans, malaria is caused by five species of the genus *Plasmodium* (Sutherland et al., 2010), of which *P. falciparum* is the most important. The escalating incidence of resistance to front-line drugs including artemisinin-based combination therapies threatens global efforts to control and eliminate malaria and highlights the urgent need to identify new chemotherapeutic strategies. In an experimental research setting, selection of drug resistance can be exploited to identify and validate drug targets, understand the molecular mechanisms underlying decreased parasite killing, guide targeted drug discovery efforts, and to improve inhibitor binding, specificity and selectivity (Ariey et al., 2014; Cowell et al., 2018; Flannery et al., 2013).

The enzyme NMT, which acylates the N-terminal glycine of substrate proteins, is a promising target for antimalarial drug development (Wright et al., 2014). This enzyme is important for the survival and viability of a wide range of parasites (*Plasmodium*, *Leishmania*, and *Trypanosoma* species) and fungi (Bell et al., 2012; Brannigan et al., 2010; Fang et al., 2015; Frearson et al., 2010; Goncalves et al., 2012b; Hutton et al., 2014; Mousnier et al., 2018; Olaleye et al., 2014; Rackham et al., 2014; Schlott et al., 2018; Tate et al., 2013; Wright et al., 2014, 2016; 2015; Yu et al., 2012), which each encode a single *nmt* gene (PF3D7_1412800). Using coenzyme A (CoA)-activated lipid, NMT transfers myristate to the substrate protein, after removal of the initiator methionine, in a predominantly co-translational process (Ritzefeld et al., 2017; Schlott et al., 2018). Given the importance of myristoylation for several vital processes in *P. falciparum* (Wright et al., 2014), the development of selective and potent small-molecule NMT inhibitors (NMTi) could form the basis of new medicines against malaria. The design of inhibitors against both *P. falciparum* (Pf) and *P. vivax* (Pv) NMTs has


© 2019 The Authors. Published by Elsevier Ltd.
This is an open access article under the CC BY license (http://creativecommons.org/licenses/by/4.0/).

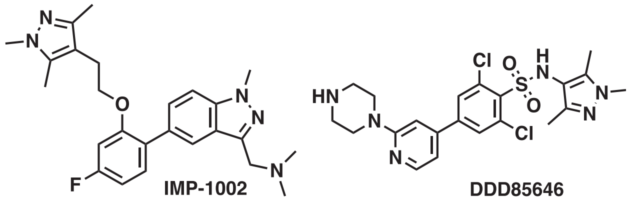


**Figure 1. Chemical Structures of NMT Inhibitors IMP-1002 and DDD86646**

focused on generating potent compounds selective over the two human (Hs) NMTs known as HsNMT1 and HsNMT2, targeting the peptide-binding pocket, and optimizing the pharmacokinetic profile. Recent developments include exploitation of inhibitor scaffolds identified in two large high-throughput screens (Bell et al., 2012; Goncalves et al., 2012b; Mousnier et al., 2018). These compounds include a quinoline series (Goncalves et al., 2012b) and four additional series used to address the issue of selectivity over host NMTs. Unfortunately, several of these chemical starting points lacked cell-based activity, potentially because of poor cellular uptake (Bell et al., 2012), as reviewed by Ritzefeld et al. (2017). A further series was explored against *Leishmania donovani* (Ld) NMT as well as PfNMT, showing improved selectivity over HsNMT1 and HsNMT2 and with promising activity against liver-stage parasites (Rackham et al., 2014, 2015; Yu et al., 2015).

Here we report discovery of an enzyme variant, PfNMT [G386E] that gives rise to parasite resistance to NMTi IMP-1002, and demonstrate its sufficiency for resistance using CRISPR-Cas9-based genome editing. The effect of this amino acid substitution on enzyme function and inhibition is explored biochemically and in parasites using a range of NMTi series. This allowed selection of compounds to solve the X-ray structure for the corresponding PvNMT wild type (WT) and variant in complex with inhibitor. Structure-guided analysis of these data enabled discovery of an inhibitor series that can overcome parasite resistance to NMTi. Collectively, our studies identify a mechanism of NMTi resistance early in the drug-development process, and provide a strategy to overcome parasite resistance to NMTi.

## RESULTS

### A Single Amino Acid Substitution in the *P. falciparum* N-Myristoyltransferase Active Site Leads to Parasite Resistance to the NMT Inhibitor IMP-1002

We first sought to generate a *P. falciparum* mutant line resistant to NMTi IMP-1002 (Figure 1). IMP-1002 is a previously undisclosed member of our recently reported series of human NMT inhibitors (Mousnier et al., 2018), and a close analog of IMP-0917 (Figure S1) that was discovered through a fragment reconstruction approach based on hits from screens against PvNMT and PfNMT. With parasite growth half maximal effective concentration ($EC_{50}$) inhibitory activity of 34 nM (Figure S2A), IMP-1002 is 4-fold more potent in killing parasites than the most potent previously reported PfNMT inhibitor DDD85646 (Figure S2A) (Wright et al., 2014). We have shown previously that *P. falciparum* 3D7 parasites normally progress to schizont forms that contain 20–30 nuclei at the end of the 48-h intracellular development in the red blood cell. Treatment at the beginning of this cycle with four times the $EC_{50}$ concentration of DDD85646 caused the parasite to develop abnormally and stall at a morphologically distinct ''pseudoschizont'' stage with 4–6 nuclei (Wright et al., 2014) (Figure S2B). Furthermore, NMTi blocked formation of the parasite inner membrane complex (IMC), a critical subcellular compartment comprising a set of flattened vesicular sacs, which form part of the parasite's surface pellicle essential for red blood cell invasion. Consistent with a conserved mode of action, at four times the $EC_{50}$ value IMP-1002 phenocopied this stage-specific block in 3D7 parasite development, preventing parasite growth beyond the four- to five-nuclei stage and inhibiting accumulation of the NMT substrate glideosome-associated protein 45 (GAP45). This protein bridges the parasite plasma membrane and the IMC. GAP45 is anchored at the plasma membrane through myristoylation and palmitoylation of its N terminus and at the IMC through palmitoylation of its C terminus, and serves as a marker for correct IMC formation and recruitment of other proteins, such as MyoA and MTIP, to form the glideosome involved in invasion (Perrin et al., 2018) (Figures S2B and S2C).

The *P. falciparum* Dd2-B2 parasite line (recloned from the Dd2 parental line by limiting dilution) was used to select for resistant parasites (Flannery et al., 2013). IMP-1002 $EC_{50}$ for Dd2 parasites was comparable with that of the 3D7 line (Figure 2A). After 36 days in the presence of 120 nM IMP-1002 (3.4 times $EC_{50}$ in this parasite line) parasite growth was detected in one culture with an initial $10^6$ parasite inoculum. Resistance to IMP-1002 was confirmed in the bulk population and three parasite clones (Figure 2B). Both IMP-1002-resistant clones showed a 14-fold increase in IMP-1002 $EC_{50}$ values (Figure 2B). To discover the genetic basis for this shift in $EC_{50}$ values, the *nmt* gene of two parasite clones as well as the resistant Dd2 bulk population was sequenced and compared with that of the Dd2 parental line. The same single non-synonymous point mutation (G1544A), leading to an amino acid substitution from glycine to glutamic acid (G386E) in the NMT protein, was present in the bulk population and each individual clone.

### CRISPR-Cas Gene Editing of *P. falciparum* 3D7 Demonstrates that PfNMT[G386E] Is Sufficient to Mediate Resistance to IMP-1002

To confirm the sufficiency of the G386E substitution for IMP-1002 resistance, we introduced the mutation into the *nmt* gene in 3D7 parasites. Genetic modification of *P. falciparum* 3D7 (Figure S3) was performed using a CRISPR-Cas9 approach (Ghorbal et al., 2014; Wagner et al., 2014; Zhang et al., 2014) as described by Knuepfer et al. (2017), using two independent guide RNA sequences to target Cas9 nuclease to the *nmt* locus. Parasites were visible 17 days post-transfection, and were subsequently screened for DNA integration, treated with 5-fluorocytosine, and cloned by limiting dilution using the plaque assay to confirm single parasites per well (Thomas et al., 2016). Integration of the single nucleotide change leading to the G386E substitution was confirmed by sequence analysis following PCR amplification of genomic DNA. The SYBR Green-based growth assay revealed a 22-fold increase in IMP-1002 $EC_{50}$ over the parental 3D7 line for the two tested clones from independent transfections

A

| | $EC_{50}$ (nM) | 95% CI (nM) |
|---|---|---|
| 3D7 parasites | 34 ± 5.8 | 29 - 41 |
| Dd2 parental | 20.4 ± 4.9 | 16 - 26 |
| uncloned population | 320 ± 50 | 270 - 370 |
| Clone 01 | 270 ± 40 | 230 - 310 |
| Clone 02 | 300 ± 140 | 220 - 490 |
| Clone 03 | 240 ± 63 | 190 - 320 |

B

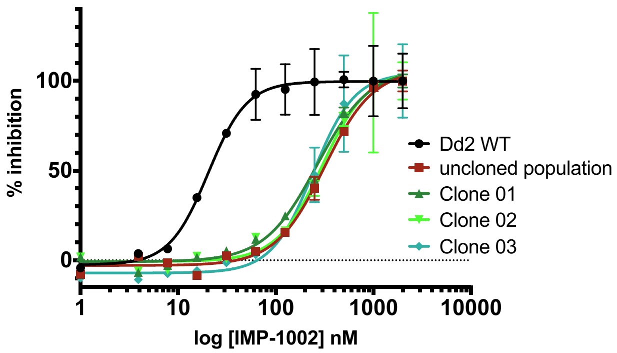


**Figure 2. IMP-1002 Inhibition of Parental and Resistant Parasite Lines**

(A) Inhibition of *P. falciparum* 3D7 and Dd2 parental parasites used to raise the resistant clones 01, 02, 03, and the uncloned population, showing the respective $EC_{50}$ and confidence interval values rounded to two significant figures.

(B) Measurement of the inhibition of growth of the Dd2 parental line, the resistant uncloned population and three IMP-1002-resistant clones to determine the $EC_{50}$ after 72 h (n = 3). There is a clear shift of the $EC_{50}$ for the resistant bulk population and the three parasite clones. Error bars indicate standard deviation of the mean.

(Figure 3A). These data confirm that this amino acid substitution is responsible for resistance to IMP-1002.

Parasites expressing PfNMT[G386E] had been selected by growth *in vitro* in the presence of IMP-1002, and it was possible that there would be a fitness cost associated with this amino acid change in absence of inhibitor, for example, by interference with the binding of one or more of the large number of substrates that are myristoylated. Therefore, we searched for any resultant phenotype at the level of protein myristoylation and long-term growth rate. To investigate potential differences in substrate engagement and myristoylation pattern, we biosynthetically labeled both WT parasites and two PfNMT[G386E] parasite clones with the myristic acid analog YnMyr throughout the complete intraerythrocytic cycle (Wright et al., 2014). There was very little difference in labeling pattern, and therefore no detectable effect of the G386E substitution on global myristoylation (Figure S4A), while continuous culture of the two PfNMT[G386E] clones revealed no significant growth defect over six generations as assessed by relative parasitemia, normalized to the WT population (Figure S4B). These data provide evidence that enzyme function is not compromised in the G386E variant.

## PfNMT[G386E] Parasites Resist Pharmacological Inhibition of Myristoylation and Parasite Development, Confirming On-Target Specificity of IMP-1002

To address the on-target specificity of IMP-1002 we investigated whether PfNMT[G386E] parasites also resist inhibition of N-myristoylation. WT and PfNMT[G386E] parasite clones were labeled with YnMyr in the presence or absence of 140 nM IMP-1002 (approximately 4-fold $EC_{50}$ for WT 3D7 parasites) during the period 36–45 h post-invasion, when myristoylation is at its peak (Wright et al., 2014). Although there was a clear decrease in YnMyr labeling in WT parasites treated with IMP-1002, there was no reduction in labeling of the PfNMT[G386E] parasite clones with the inhibitor present (Figure 3B).

To determine whether PfNMT[G386E] parasite clones develop normally in the presence of 140 nM IMP1002, synchronized ring stages from WT parasites and one PfNMT[G386E] clone were treated with either 140 nM IMP-1002 or control DMSO for a period of 45 h. We then determined the number of nuclei within the cells by flow cytometry (Figure 3C). In the presence of IMP-1002, the WT parasite population contained a reduced number of nuclei compared with the DMSO control, while PfNMT [G386E] parasites showed no difference between IMP-1002-treated parasites at 140 nM and DMSO-treated control parasites (Figures 3C and 3D).

## PvNMT[G386E] Is Refractory to Binding and Inhibition by IMP-1002

We next turned our attention to the biochemical basis for resistance to IMP-1002 *in vitro* using *P. vivax* NMT (PvNMT) as a tractable model for PfNMT. PvNMT possesses 80% sequence identity and 93% similarity to PfNMT and is an excellent platform for structural studies, while to date PfNMT has proven intractable for X-ray crystallography. Activity measurements with PvNMT[WT] and PvNMT[G386E] enzymes used MyrCoA and a 15-residue NMT substrate peptide. The enzymes were of similar purity (Figure S5B) and had similar kinetic profiles over 45 min in a continuous assay (Figure S5A). Reactions used a coumarin derivative containing a thiol-reactive maleimide called 7-diethylamino-3-(4-maleimidophenyl)-4-methylcoumarin (CPM), to monitor the formation of the CoA thiol product (Goncalves et al., 2012a). A quenched assay after 30 min was used to determine IMP-1002 half maximal inhibitory concentration ($IC_{50}$) for PvNMT[G386E] compared with PvNMT[WT], which indicated a shift to higher concentration for the variant enzyme visualized through a ratio of G386E/WT of 2.6 on average (Figure 4A). In comparison, the ratio of G386E/WT $EC_{50}$ values in the parasite growth assay, was approximately 20-fold (Figures 3A and 4B). With the high potency of IMP-1002, the CPM assay is at its detection limit resulting in a reduced shift in the variant enzyme. To complement the CPM assay we performed a direct binding thermal shift assay with PvNMT[WT] and PvNMT[G386E]. Consistent with the high affinity of IMP-1002 for the PvNMT[WT] this protein was stabilized by >8.8°C with 5 μM of IMP-1002 even in the absence of MyrCoA. In contrast, the thermal stabilization was only 1.8°C with PvNMT[G386E], leading to a $\Delta\Delta T_m$ of >7.0°C between the two enzymes (Figure 4C; Table S4). The same assay was conducted in the presence of 4 μM MyrCoA, which showed a similar trend; however,

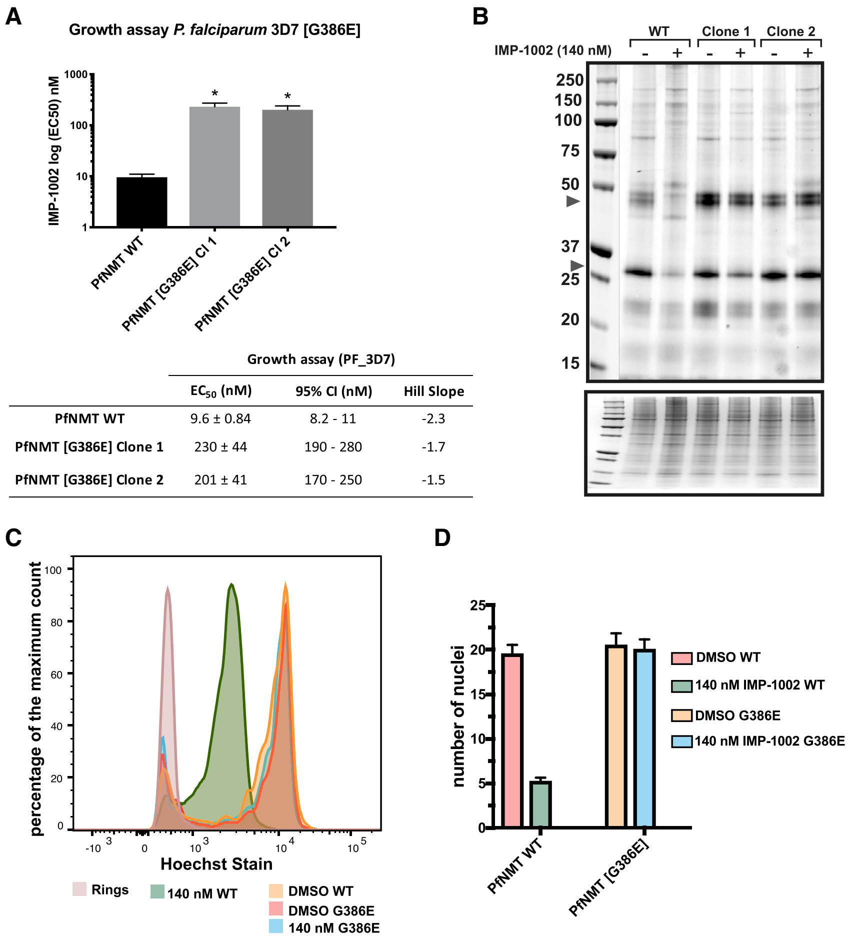


Growth assay (PF_3D7)

| | $EC_{50}$ (nM) | 95% CI (nM) | Hill Slope |
|---|---|---|---|
| PfNMT WT | 9.6 ± 0.84 | 8.2 - 11 | -2.3 |
| PfNMT [G386E] Clone 1 | 230 ± 44 | 190 - 280 | -1.7 |
| PfNMT [G386E] Clone 2 | 201 ± 41 | 170 - 250 | -1.5 |

**Figure 3. NMTi IMP-1002 Shows On-Target Specificity**

(A) Growth assay with *P. falciparum* 3D7[G386E] clones and [WT] showing shift in $EC_{50}$ for both clones from transfections with independent guides (n = 3). Unpaired Welch t test not assuming equal standard deviations, p = 0.01.

(B) NMT substrates labeled with YnMyr in WT and G386E parasites after treatment with NaOH to hydrolyze the base-labile ester-linkage incorporation of YnMyr into GPI-anchored proteins. Although WT parasites show a decreased band intensity in response to the NMTi treatment, G386E parasite proteins remain unchanged compared with those of the DMSO control. The Coomassie blue-stained gel shows proportional loading of total protein for each sample.

(C) Percentage of maximum count of Hoechst-stained WT and G386E parasites used to determine the number of nuclei per sample.

(D) *P. falciparum* [G386E] parasites show 0% inhibition at 140 nM. Graph shows flow cytometry data of Hoechst-stained parasites in (C) treated with 140 nM IMP-1002 from 1 to 45 h post-invasion. The median fluorescent intensity (MFI) of the Hoechst-positive parasites was normalized to the MFI of parasites containing one nucleus (ring sample) in (C) to determine the average number of nuclei per sample. While WT parasites show a drop-in the number of nuclei and therefore an arrest in development at 140 nM of NMTi, G386E parasites show no arrest in development. All errors bars in this figure indicate standard deviation of the mean.

because of the higher baseline melting temperature for the MyrCoA-bound proteins the stabilization window was compressed (Table S4B).

## X-Ray Crystallography Identifies the Structural Basis for IMP-1002 Resistance in PvNMT[G386E]

Having demonstrated that the G386E substitution in NMT was responsible for parasite resistance to NMTi, we next focused on understanding the structural basis for resistance. G386 does not directly contact the inhibitor in previously reported NMT co-crystal structures (Bell et al., 2012; Mousnier et al., 2018; Wright et al., 2014) leading us to consider a hypothesis based on indirect conformational changes in the inhibitor binding site. In contrast to G386, E386 places a large, charged side chain in direct proximity to the nearby Y211 residue. From comparisons of PvNMT and HsNMT1, Y211 is known to adopt at least two different preferred rotamers, with HsNMT showing a strong preference for one of these conformations; compounds which disfavor this preferred rotamer can achieve a high degree of selectivity for parasite NMT over HsNMT (Brannigan et al., 2014; Yu et al., 2012). The binding mode of IMP-1002 suggests that it packs tightly against Y211 (Figure 5B) and shows good selectivity over HsNMT, but this binding mode leaves little space for a glutamate side chain at position 386.

To test this hypothesis experimentally, recombinant PvNMT [G386E] was crystallized in the presence of MyrCoA ± IMP-1002 (PDB: 6MB0) (Table S1). There is minimal difference in the position of Y211 between the crystal structure of PvNMT [G386E] with bound MyrCoA (but no inhibitor) (PDB: 6MAY) and that of PvNMT[WT] (PDB: 2YNC) (Wright et al., 2014) (Figure 5A), with the tyrosine adopting one of two observed rotamers (designated "rotamer Y211a") in the PvNMT[G386E] protein crystal and both rotamers Y211a and Y211b in the WT, indicating a degree of flexibility. Interestingly, the nearby His213 displays a larger degree of flexibility in the PvNMT [WT] structure, modeled as two possible rotamers (H213a, H213b), whereas the residue adopts a single conformation in the G386E variant (H213a). On IMP-1002 interaction with PvNMT[WT] (PDB: 6MB1), Y211 moves to a previously unobserved third rotamer conformation (Y211c) to form a $\pi$-$\pi$ stacking interaction with the pendant phenyl ring of IMP-1002, while H213 remains in the unliganded position H213a (Figure 5B). On IMP-1002 binding to PvNMT[G386E], the glutamate side chain prevents formation of the more favorable $\pi$-$\pi$ stacking interaction with the pendant phenyl ring, and forces H213 to move from rotamer position H213a to the more constrained H213b (Figure 5B). Both Y211 and H213 thus adopt alternative rotamers from those seen in PvNMT[WT] to create space for the inhibitor, leading to the loss of beneficial $\pi$-$\pi$

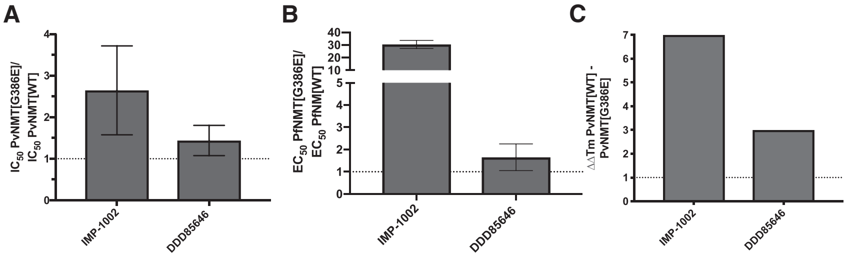


**Figure 4. Consequences of G386E Substitution for NMT Function**

Bar graphs showing the effect of the G386E substitution on (A) $IC_{50}$ in the biochemical assay, (B) $EC_{50}$ in the parasite growth assay, and (C) thermal shift in a protein stability assay with IMP-1002 (n = 2–3). For comparison, the effect of the substitution on these values is shown for another NMT inhibitor, DDD85646, which is not affected. All errors bars in this figure indicate standard deviation of the mean. For Figure 4C refer to the Tables S4A and S4B.

stacking interactions consistent with the observed weaker affinity of IMP-1002 for the G386E variant (Figure 5B).

## Predictive Structure-Guided Identification of NMT Inhibitor that Overcomes G386E Resistance Both Biochemically and in the Parasite

Based on these structural insights, we explored NMTi binding modes that might deliver resistance-breaking activity. Examination of our previously reported structure of NMTi DDD85646 in complex with PvNMT[WT] (PDB: 2YND) (Wright et al., 2014) revealed a binding mode distinct from that of IMP-1002, which largely avoids direct interactions with Y211 (Figure 5C). Therefore, we measured the $IC_{50}$ of DDD85646 with PvNMT[WT] and [G386E] enzyme and its $EC_{50}$ with Pf3D7 [WT] and [G386E] parasite clones; compared with IMP-1002, the ratio of G386E/WT for DDD85646 was between 1 and 1.7 in both assays (CPM assay, $IC_{50}$ ratio = 1.4 ± 0.35; growth assay, $EC_{50}$ ratio = 1 ± 0.1), strengthening the hypothesis that DDD85646 binding is unaffected by G386E (Figures 4A and 4B; Tables S2 and S3). This was supported by thermal shift data with DDD85646 that confirmed the difference in stabilization ($\Delta\Delta T_m$) between PvNMT[WT] and PvNMT[G386E] was only 3°C, significantly less than the >7.0°C in favor of WT observed for IMP-1002 (Table S4A).

Structural studies supported this concept. We solved the structure of PvNMT[G386E] with bound DDD85646 (PDB: 6MAZ) (Table S1), and discovered that there is minimal movement of either Y211 or H213 in the inhibitor-bound form compared with PvNMT[WT], confirming the molecular basis for similar affinity (Figure 5C). Analysis of the PvNMT[G386E] structure with either IMP-1002 or DDD85646 bound demonstrated that the positions of all three amino acids (E386, Y211, and H213) were influenced by the binding mode of the inhibitor (Figure 5D). By including the PvNMT[WT] structure in the comparison, Y211 was observed to adopt at least four different conformations. A preferred conformation (rotamer Y211a) was adopted in both the unliganded PvNMT[WT] and [G386E] enzymes, and, on binding of DDD85646 to PvNMT [WT], Y211 moved only slightly to rotamer Y211b when DDD85646 was bound to PvNMT[G386E]. For PvNMT[WT] with bound IMP-1002, Y211 adopts rotamer Y211c, allowing the $\pi$-$\pi$ stacking interaction with IMP-1002, which likely results in the increased potency and selectivity (over HsNMT) of this compound compared with DDD85646. However, for PvNMT [G386E] with bound IMP-1002, Y211 moves to rotamer position Y211d to avoid a steric clash with E386: this restricts the ability of Y211 to adopt its preferred conformation, preventing the $\pi$-$\pi$ stacking interaction with IMP-1002 and leading to the shift of H213 from H213a to H213b. Figure 5D also shows the considerable flexibly of E386 in the different structures. We found, depending on the inhibitor bound (IMP-1002, DDD85646 or in the apo form), that E386 can adopt up to four distinct occupancies showing its flexible nature. Overall the minimal disruption of Y211 and H213 by DDD85646 binding results in the conserved affinity and binding mode for both WT and G386E variants (Figure 5C), and these data together with minimally perturbed $IC_{50}$, $EC_{50}$, and thermal stability (Figure 4; Tables S2, S3, and S5) lead us to conclude that DDD85646 can break resistance in NMT[G386E] parasites.

To analyze further the binding mode of other NMT inhibitors, the published crystal structures of PvNMT[WT] with two bound NMTi, IMP-0320 (PDB: 2YNE) (Wright et al., 2014) and IMP-0856 (PDB: 4UFX) (Yu et al., 2015) (Figures 1 and S6B) were overlaid on the PvNMT[G386E] structure to examine whether the binding of these inhibitors would be affected by the G386E substitution. The movement of Y211 from position Y211c to Y211d, accompanied by the prevention of favorable interactions with both inhibitors, led us to hypothesize that their binding would also be affected by the G386E substitution, unlike DDD85646. To test this, we performed the same $EC_{50}$ shift assay with the parasite and $IC_{50}$ determination with the CPM assay using IMP-0964 (a close derivative of IMP-0856) and IMP-0320. We also included the NMTi IMP-0917, a close derivative of IMP-1002 that has recently been reported (Mousnier et al., 2018), which we expected to behave like IMP-1002 because of its similar structural properties (Figure S1). Shifts in $EC_{50}$ (Figure 6A; Table S2) correlated very well with shifts in $IC_{50}$ (Figure 6B; Table S3). These results confirmed that binding of both IMP-0320 and IMP-0964 were affected by the G386E substitution. In the same experiments, the potency of DDD85646 in killing parasites in the cell-based assay (Figure 6A) and binding in the enzyme assay (Figure 6B) was unaffected by the G386E change.

To complement the CPM assay and potentially increase the accuracy of measuring $IC_{50}$ values below 10 nM in the case of highly potent compounds, we used surface plasmon resonance (SPR) to determine the relative $K_D$ values of different NMTi with the two forms of the enzyme. SPR improved differentiation between the binding affinities of NMTi for PvNMT[WT] and PvNMT[G386E], as summarized in Figure S5C and Table S6. The affinities of IMP-1002 and IMP-0917 decreased by approximately 5-fold because of the substitution, whereas binding of DDD85646 was relatively insensitive. IMP-0320 and IMP-0964 showed the most marked decrease in affinity, almost 100- and 20-fold, respectively, between the PvNMT[G386E] and [WT] protein (Figures 6C and S5C; Table S6).

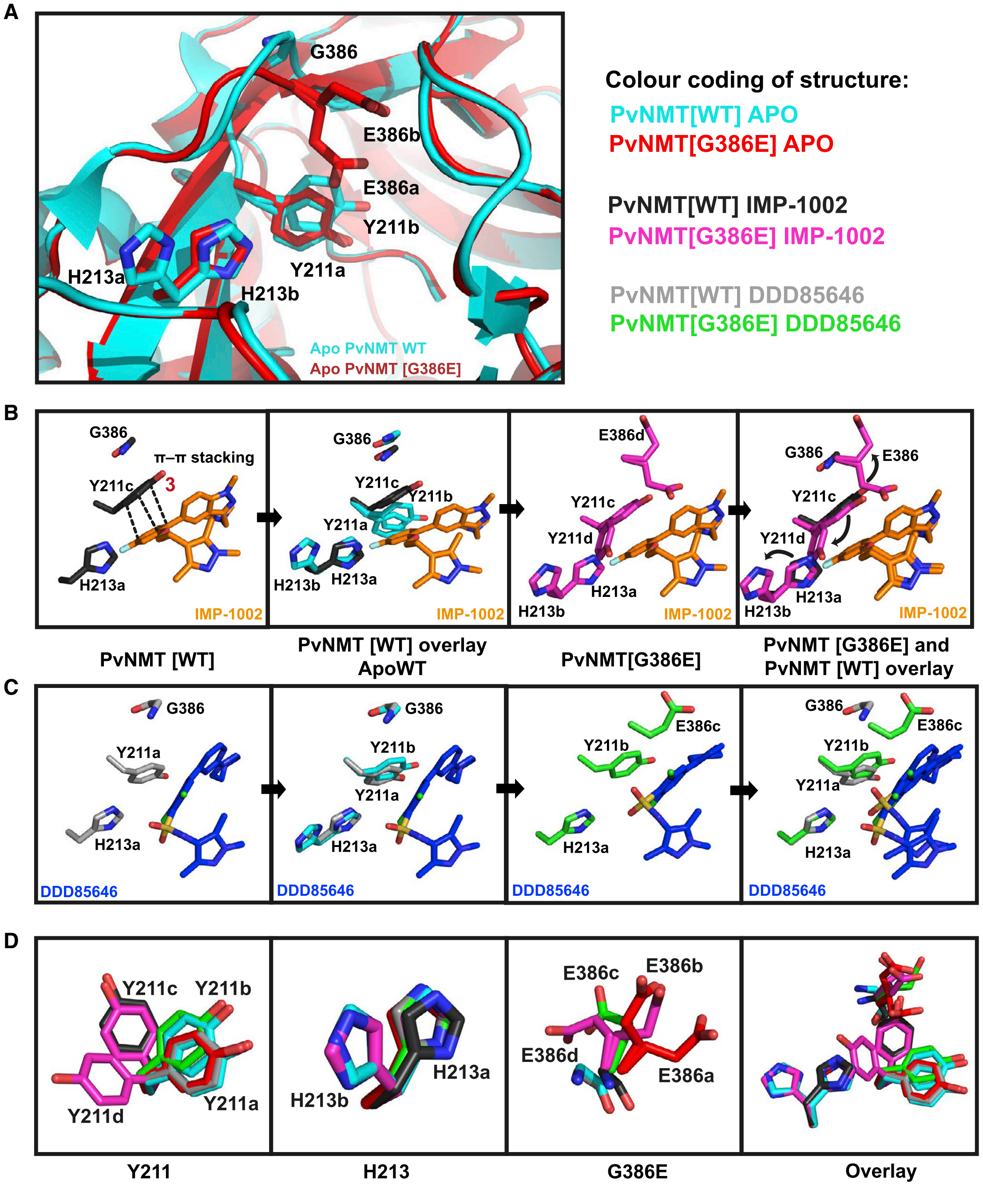


**Figure 5. Crystallographic Analysis of NMT Inhibitor Interaction with PvNMT[WT] and PvNMT[G386E]**

(A) Crystal structure of PvNMT[WT] active site without inhibitor bound, overlaid with PvNMT[G386E].

(B) Movement of residues in the active site of PvNMT[G386E] compared with PvNMT[WT] on interaction with IMP-1002.

(C and D) (C) Movement of residues in the active site of PvNMT[G386E] compared with PvNMT[WT] on DDD85646 binding (D) Y211 movement in the active site in the six different crystal structures. Y211 can adopt four distinct rotamers depending on the G386E mutation and type of inhibitor bound. This is followed by H213 that can adopt two different rotamer occupancies to accommodate the extra space taken up by Y211 when G386 is mutated to E386.

The kinetic differences between the compounds with the WT and G386E enzymes reflected this generally weaker binding, with faster off rates apparent from the sensorgram traces. IMP-1002 and IMP-0917 exhibited interesting kinetics: both showed a slow dissociation rate with PvNMT[WT] (Figure S5C), suggesting that several hours may be required for complete dissociation. Although the effect was largest for IMP-1002, the long dissociation rates of both compounds probably led to an underestimate of their affinities because of reduced binding capacity in sequential injections during a standard equilibrium $K_D$

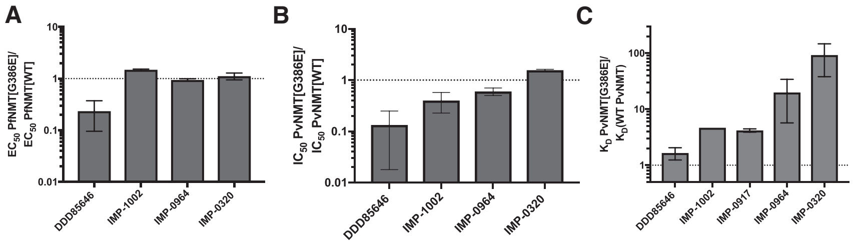


**Figure 6. The NMT[G386E] Variant is Affected Differently by a Range of NMT Inhibitors**

(A) Mean increase of $EC_{50}$ between PfNMT[G386E] and PfNMT[WT] parasites measured through SYBR Green growth assay (visualized on a log scale) (n = 2). A mean increase of 10 is indicated by a dotted line.

(B) Enzymatic CPM assay measuring the mean increase in $IC_{50}$ with different NMTi with purified PvNMT[WT] and PvNMT[G386E] (visualized on a log scale) (n = 3). A mean increase of 10 is indicated by a dotted line.

(C) Mean increase in binding affinity of NMTi for PvNMT[WT] and PvNMT[G386E] (visualized on a log scale) (n = 1–3). The $K_D$ of each compound with PvNMT[G386E] was divided by the $K_D$ with PvNMT[WT] to calculate the fold difference. $K_D$ is the equilibrium dissociation constant of NMTi calculated from fitted response-concentration plots measured by SPR analyses. Only one $K_D$ could be accurately determined for IMP-1002 and so error bars are not shown. A mean increase of 10 is indicated by a dotted line. All errors bars in this figure indicate standard deviation of the mean.

SPR analysis. In combination with the shift in $EC_{50}$, $IC_{50}$, and thermal stability, the SPR data clearly show that DDD85646 can overcome resistance mediated by G386E.

## DISCUSSION

The current study describes identification and validation of the consequence of a single amino acid substitution (G386E) in the substrate-binding pocket of *Plasmodium* NMT, identified by drug selection of a non-synonymous point mutation in the *nmt* open reading frame. The importance of this mutation in *P. falciparum* parasites for this drug target, was confirmed by CRISPR-Cas9-mediated substitution of this single nucleotide in the WT parasite, recapitulating resistance against three chemically distinct NMT inhibitor series. Data from the PfNMT [G386E]-resistant parasite line were used to guide biochemical and structural analysis of this variant in recombinant PvNMT, providing both enzymatic inhibition data and the crystal structures of the WT and G386E variant with bound MyrCoA and the NMT inhibitor (NMTi) used for mutation selection. Structural, $IC_{50}$ and $K_D$ data for PvNMT[G386E] are consistent and correlated with the $EC_{50}$ shift data obtained in G386E parasites, although the active site of PvNMT differs from PfNMT in 2 out of 23 residues (F212Y and F334Y, PfNMT to PvNMT) (Yu et al., 2012). These structural data provide insights into changes in the active site induced by the G386E substitution, including the modes of binding for different inhibitors and the flexibility required within the peptide-binding pocket to accommodate them. Using these data, a structure-guided approach was used to identify compounds that can overcome resistance mediated by the G386E substitution, exploiting the differential binding mode of DDD85646. This predictive structural model can guide further discovery of drugs active against NMT, and current work is focused on optimizing a new lead inhibitor series unaffected by G386E. The identification of NMTi that circumvent the G386E mutation suggests opportunities to combine different inhibitors to overcome selective resistance, and thus thwart the emergence of resistant variants.

Interestingly, the G386E variant parasite shows no apparent reduction of fitness in growth at the asexual blood stage, although it is currently unknown whether this observation would apply throughout the complete parasite life cycle. Even though G386E reduces the space in the peptide-binding pocket, the flexibility of Y211, E386, and H213 (Figure 5C) provides a plausible explanation for why substrate myristoylation is unaffected, leading to no reduction in fitness or change in global myristoylation (Figure S3). Future studies on the potential of various substrates with different sequences downstream of the N-terminal glycine to differentially stabilize intermediate states during the catalytic cycle of NMT, in combination with studies on the flexibility of turn-over of different substrates in the WT and G386E variant NMTs, could give further insights into substrate specificity.

Together, these data provide direct genetic evidence for the specific on-target activity of each of the NMTi classes described here, which, in combination with our previously reported chemical biology studies (Wright et al., 2014), further supports the validity of this target in malaria. The relatively fast selection of resistance to NMTi may suggest the potential for rapid development of resistance in the clinic. However, the SNP responsible for G386E is not present in any of the field isolates cataloged in PlasmoDB. In the 202 available isolates, there are 6 non-synonymous mutations in the coding region resulting in five amino acid changes, but these are all distal to the substrate-binding site and unlikely to interfere with NMTi binding. Furthermore, the repeated selection of this particular mutant in the Dd2 cloned line over a short period of drug pressure may imply the rapid acquisition of this mutation. We would therefore expect to see this mutation arising in the field when drug pressure is applied, considering that there was no fitness cost associated with G386E *in vitro*. Structural biology has been applied extensively to drug resistance in other fields including influenza (Wu et al., 2016), hepatitis C (Lagacé et al., 2011), and HIV (Slaughter et al., 2014), highlighting the general importance of structure-guided design for the discovery of resistance-breaking inhibitors.

In the future it would be of interest to attempt selection of parasites resistant to DDD85646, because the on-target activity of DDD85646 (compound 1a in Wright et al., 2014) rules out an off-target killing of PfNMT[G386E] parasites. DDD85646 is insensitive to the resistance mechanism generated in response to IMP-1002 treatment, but at a cost of lower selectivity over HsNMT (Table S5), because Y211 needs to move from the "in" Y211c rotamer occupancy, which provides improved selectivity over HsNMT (Yu et al., 2012), to the "out" Y211d rotamer, leaving room for G386E. Future studies will focus on identification of an inhibitor series that can overcome the G386E-mediated resistance without compromising selectivity,

based on either IMP-1002 analogs or, more likely, a new chemical series with a distinct binding mode. For example, the series that includes IMP-1002 has recently been adapted to increase HsNMT selectivity with a relatively simple adaptation (Mousnier et al., 2018). This shows that the flexibility of the series has the scope to find analogs of IMP-1002 that can also overcome G386E-mediated resistance. In summary, this structure-guided analysis provides mechanistic rationalization and enables discovery of an NMTi that can overcome the identified NMT resistance mechanism resulting from the G386E substitution. This approach defines a strategy to overcome acquisition of NMTi resistance, and will facilitate further drug development efforts.

## SIGNIFICANCE

**Malaria is one of the most significant infectious diseases worldwide, and the escalating threat of parasite resistance to all current antimalarial drugs highlights the need for drugs with new modes of action. The enzyme NMT is an antimalarial target, and here we provide genetic evidence for the on-target activity of several classes of NMT inhibitors through resistance selection. High-resolution crystal structures of NMT and the resistant variant provide deep insights into the flexibility of the substrate-binding pocket and allowed us to identify an inhibitor to overcome resistance. This work reveals the possibility of optimizing NMT inhibitors by incorporating resistance studies early in the drug discovery process, an approach with wider application in drug discovery and development.**

## STAR★METHODS

Detailed methods are provided in the online version of this paper and include the following:

- KEY RESOURCES TABLE
- CONTACT FOR REAGENT AND RESOURCE SHARING
- EXPERIMENTAL MODEL AND SUBJECT DETAILS
  - Parasite Culture
- METHOD DETAILS
  - Synthesis Route of IMP-1002
  - Indirect Immunofluorescence Assay (IFA)
  - Drug Treatment and $EC_{50}$ Determination Using the SYBR Green Assay
  - Selection of IMP-1002-Resistant *P. falciparum* Dd2 Parasites Using a Single-Step Strategy
  - NMT gDNA Amplification and Sequencing from IMP-1002 Resistant *P. falciparum*
  - Cloning of Constructs and Transfection of 3D7 *P. falciparum*
  - Parasite Growth Assay
  - Metabolic Tagging of Parasites and CuAAC Labelling
  - Base Treatment to Cleave the Esters of GPI-Anchored Proteins
  - Flow Cytometry
  - Cloning, Expression and Purification of PvNMT
  - CPM-Based PvNMT $IC_{50}$ Determination
  - Thermal Shift Assay
  - Surface Plasmon Resonance (SPR) Assay
  - Crystallography
- QUANTIFICATION AND STATISTICAL ANALYSIS
- DATA AND SOFTWARE AVAILABILITY

## SUPPLEMENTAL INFORMATION

Supplemental Information can be found online at https://doi.org/10.1016/j.chembiol.2019.03.015.

## ACKNOWLEDGMENTS

A.C.S. was a Francis Crick Institute/Imperial College London Ph.D. student. Work in the Holder laboratory and Tate satellite laboratory was supported by the Francis Crick Institute, which receives its core funding from Cancer Research UK (FC001097, FC010636), the UK Medical Research Council (FC001097, FC010636), and the Wellcome Trust (FC001097, FC010636). We like to thank the Flow Cytometry STP (especially Philip Hobson and Graham Preece) at the Francis Crick Institute for helping with the flow cytometry data. We thank Adam Flinders for support with SPR and thermal stability assays at GlaxoSmithKline UK. E.W.T. and A.S.B. thank Medicines for Malaria Venture for support (project no. 15/0054). The Seattle Structural Genomics Center for Infectious Disease (SSGCID) is funded in part with Federal funds from the National Institute of Allergy and Infectious Diseases, NIH, Department of Health and Human Services, under contract no. HHSN272201700059C. B.L.S. is supported in part by NIAID grant 1R21AI137815-01. D.A.F. gratefully acknowledges funding support from the Medicines for Malaria Venture (MMV). MMV partially funded the work and MMV donors are listed on the website (http://www.mmv.org/about-us/our-donors). The authors declare that they have no competing interests, beyond the fact that MMV is involved in supporting the development of malaria medicines.

## AUTHOR CONTRIBUTIONS

Conceptualization, A.C.S., E.W.T., and A.A.H.; Methodology, A.C.S., S.M., J.G., and E.K.; Investigation, A.C.S., S.M., A.R.R., O.C.-F., A.S.B., J.G., E.K., D.C., and S.L.-A.; Supervision, B.L.S., C.-W.C., P.J.M., D.A.F., E.W.T., and A.A.H.; Writing – Original Draft, A.C.S., E.W.T., and A.A.H.; Writing – Review & Editing, A.C.S., E.W.T., and A.A.H.; Visualization, A.C.S.; Project Administration, A.C.S.; Funding Acquisition, R.B., B.C., J.B., E.W.T., and A.A.H.

## DECLARATION OF INTERESTS

A.S.B. and E.W.T. are inventors on a patent application that describes NMT inhibitor IMP-1002 (Bell, A.C.S.; Tate, E.W.; Leatherbarrow, R.J.; Hutton, J.A.; Brannigan, J.A., Compounds and their use as inhibitors of N-myristoyl transferase, Patent Cooperation Treaty International Application (2017) WO 2017001812).

Received: January 2, 2019
Revised: February 25, 2019
Accepted: March 25, 2019
Published: May 9, 2019

## REFERENCES

Adams, P.D., Afonine, P.V., Bunkóczi, G., Chen, V.B., Davis, I.W., Echols, N., Headd, J.J., Hung, L.-W., Kapral, G.J., Grosse-Kunstleve, R.W., et al. (2010). PHENIX: a comprehensive Python-based system for macromolecular structure solution. Acta Crystallogr. D Biol. Crystallogr. *66*, 213–221.

Ariey, F., Witkowski, B., Amaratunga, C., Beghain, J., Langlois, A.-C., Khim, N., Kim, S., Duru, V., Bouchier, C., Ma, L., et al. (2014). A molecular marker of artemisinin-resistant *Plasmodium falciparum* malaria. Nature *505*, 50–55.

Bell, A.S., Mills, J.E., Williams, G.P., Brannigan, J.A., Wilkinson, A.J., Parkinson, T., Leatherbarrow, R.J., Tate, E.W., Holder, A., and Smith, D.F. (2012). Selective inhibitors of protozoan protein N-myristoyltransferases as

starting points for tropical disease medicinal chemistry programs. PLoS Negl. Trop. Dis. *6*, e1625.

Brannigan, J.A., Roberts, S.M., Bell, A.S., Hutton, J.A., Hodgkinson, M.R., Tate, E.W., Leatherbarrow, R.J., Smith, D.F., and Wilkinson, A.J. (2014). Diverse modes of binding in structures of *Leishmania* major N-myristoyltransferase with selective inhibitors. IUCrJ *1*, 250–260.

Brannigan, J.A., Smith, B.A., Yu, Z., Brzozowski, A.M., Hodgkinson, M.R., Maroof, A., Price, H.P., Meier, F., Leatherbarrow, R.J., Tate, E.W., et al. (2010). N-Myristoyltransferase from *Leishmania donovani*: structural and functional characterisation of a potential drug target for visceral leishmaniasis. J. Mol. Biol. *396*, 985–999.

Bryan, C.M., Bhandari, J., Napuli, A.J., Leibly, D.J., Choi, R., Kelley, A., Van Voorhis, W.C., Edwards, T.E., and Stewart, L.J. (2011). High-throughput protein production and purification at the Seattle structural genomics center for infectious disease. Acta Crystallogr. Sect. F Struct. Biol. Cryst. Commun. *67*, 1010–1014.

Chen, V.B., Arendall, W.B., Headd, J.J., Keedy, D.A., Immormino, R.M., Kapral, G.J., Murray, L.W., Richardson, J.S., and Richardson, D.C. (2010). MolProbity: all-atom structure validation for macromolecular crystallography. Acta Crystallogr. D Biol. Crystallogr. *66*, 12–21.

Choi, R., Kelley, A., Leibly, D., Hewitt, S.N., Napuli, A., and Van Voorhis, W. (2011). Immobilized metal-affinity chromatography protein-recovery screening is predictive of crystallographic structure success. Acta Crystallogr. Sect. F Struct. Biol. Cryst. Commun. *67*, 998–1005.

Cowell, A.N., Istvan, E.S., Lukens, A.K., Gomez-Lorenzo, M.G., Vanaerschot, M., Sakata-Kato, T., Flannery, E.L., Magistrado, P., Owen, E., Abraham, M., et al. (2018). Mapping the malaria parasite druggable genome by using in vitro evolution and chemogenomics. Science *359*, 191–199.

Emsley, P., and Cowtan, K. (2004). Coot: model-building tools for molecular graphics. Acta Crystallogr. D Biol. Crystallogr. *60*, 2126–2132.

Evans, P. (2006). Scaling and assessment of data quality. Acta Crystallogr. D Biol. Crystallogr. *62*, 72–82.

Fang, W., Robinson, D.A., Raimi, O.G., Blair, D.E., Harrison, J.R., Lockhart, D.E.A., Torrie, L.S., Ruda, G.F., Wyatt, P.G., Gilbert, I.H., et al. (2015). N-Myristoyltransferase is a cell wall target in *Aspergillus fumigatus*. ACS Chem. Biol. *10*, 1425–1434.

Flannery, E.L., Fidock, D.A., and Winzeler, E.A. (2013). Using genetic methods to define the targets of compounds with antimalarial activity. J. Med. Chem. *56*, 7761–7771.

Frearson, J.A., Brand, S., McElroy, S.P., Cleghorn, L.A.T., Smid, O., Stojanovski, L., Price, H.P., Guther, M.L.S., Torrie, L.S., Robinson, D.A., et al. (2010). N-Myristoyltransferase inhibitors as new leads to treat sleeping sickness. Nature *464*, 728–732.

Gerold, P., Schofield, L., Blackman, M.J., Holder, A.A., and Schwarz, R.T. (1996). Structural analysis of the glycosyl-phosphatidylinositol membrane anchor of the merozoite surface proteins-1 and -2 of *Plasmodium falciparum*. Mol. Biochem. Parasitol. *75*, 131–143.

Ghorbal, M., Gorman, M., Macpherson, C.R., Martins, R.M., Scherf, A., and Lopez-Rubio, J.J. (2014). Genome editing in the human malaria parasite *Plasmodium falciparum* using the CRISPR-Cas9 system. Nat. Biotechnol. *32*, 819–821.

Goncalves, V., Brannigan, J.A., Thinon, E., Olaleye, T.O., Serwa, R., Lanzarone, S., Wilkinson, A.J., Tate, E.W., and Leatherbarrow, R.J. (2012a). A fluorescence-based assay for N-myristoyltransferase activity. Anal. Biochem. *421*, 342–344.

Goncalves, V., Brannigan, J.A., Whalley, D., Ansell, K.H., Saxty, B., Holder, A., Wilkinson, A.J., Tate, E.W., and Leatherbarrow, R.J. (2012b). Discovery of *Plasmodium vivax* N-myristoyltransferase inhibitors: screening, synthesis, and structural characterization of their binding mode. J. Med. Chem. *55*, 3578–3582.

Green, J.L., Moon, R.W., Whalley, D., Bowyer, P.W., Wallace, C., Rochani, A., Kumar, R., Howell, S.A., Grainger, M., Jones, H.M., et al. (2015). Imidazopyridazine inhibitors of *Plasmodium falciparum* calcium-dependent protein kinase 1 also target cyclic GMP-dependent protein kinase and heat shock protein 90 to kill the parasite at different stages of intracellular development. Antimicrob. Agents Chemother. *60*, 1464–1475.

Harris, P.K., Yeoh, S., Dluzewski, A.R., O'Donnell, R.A., Withers-Martinez, C., Hackett, F., Bannister, L.H., Mitchell, G.H., and Blackman, M.J. (2005). Molecular identification of a malaria merozoite surface sheddase. PLoS Pathog. *1*, 241–251.

Heal, W.P., Wright, M.H., Thinon, E., and Tate, E.W. (2011). Multifunctional protein labeling via enzymatic N-terminal tagging and elaboration by click chemistry. Nat. Protoc. *7*, 105–117.

Hutton, J.A., Goncalves, V., Brannigan, J.A., Paape, D., Wright, M.H., Waugh, T.M., Roberts, S.M., Bell, A.S., Wilkinson, A.J., Smith, D.F., et al. (2014). Structure-based design of potent and selective *Leishmania* N-myristoyltransferase inhibitors. J. Med. Chem. *57*, 8664–8670.

Kabsch, W. (2010). Integration, scaling, space-group assignment and post-refinement. Acta Crystallogr. D Biol. Crystallogr. *66*, 133–144.

Knuepfer, E., Napiorkowska, M., van Ooij, C., and Holder, A. (2017). Generating conditional gene knockouts in *Plasmodium* - a toolkit to produce stable DiCre recombinase-expressing parasite lines using CRISPR/Cas9. Sci. Rep. *7*, 3881.

Lagacé, L., White, P.W., Bousquet, C., Dansereau, N., Dô, F., Llinas-Brunet, M., Marquis, M., Massariol, M.-J., Maurice, R., Spickler, C., et al. (2011). In vitro resistance profile of the hepatitis C virus NS3 protease inhibitor BI 201335. Antimicrob. Agents Chemother. *56*, 569–572.

Lim, M.Y.-X., LaMonte, G., Lee, M.C.S., Reimer, C., Tan, B.H., Corey, V., Tjahjadi, B.F., Chua, A., Nachon, M., Wintjens, R., et al. (2016). UDP-galactose and acetyl-CoA transporters as *Plasmodium* multidrug resistance genes. Nat. Microbiol. *1*, 16166.

Macpherson, C.R., and Scherf, A. (2015). Flexible guide-RNA design for CRISPR applications using Protospacer Workbench. Nat. Biotechnol. *33*, 805–806.

McNicholas, S., Potterton, E., Wilson, K.S., and Noble, M.E.M. (2011). Presenting your structures: the CCP4mg molecular-graphics software. Acta Crystallogr. D Biol. Crystallogr. *67*, 386–394.

Moon, R.W., Hall, J., Rangkuti, F., Ho, Y.S., Almond, N., Mitchell, G.H., Pain, A., Holder, A., and Blackman, M.J. (2013). Adaptation of the genetically tractable malaria pathogen *Plasmodium knowlesi* to continuous culture in human erythrocytes. Proc. Natl. Acad. Sci. U S A *110*, 531–536.

Motulsky, H.J., and Brown, R.E. (2006). Detecting outliers when fitting data with nonlinear regression - a new method based on robust nonlinear regression and the false discovery rate. BMC Bioinformatics *7*, 123.

Mousnier, A., Bell, A.S., Swieboda, D.P., Morales-Sanfrutos, J., Pérez-Dorado, I., Brannigan, J.A., Newman, J., Ritzefeld, M., Hutton, J.A., Guedán, A., et al. (2018). Fragment-derived inhibitors of human N-myristoyltransferase block capsid assembly and replication of the common cold virus. Nat. Chem. *12*, 1–606.

Olaleye, T.O., Brannigan, J.A., Roberts, S.M., Leatherbarrow, R.J., Wilkinson, A.J., and Tate, E.W. (2014). Peptidomimetic inhibitors of N-myristoyltransferase from human malaria and leishmaniasis parasites. Org. Biomol. Chem. *12*, 8132–8137.

Perrin, A.J., Collins, C.R., Russell, M.R.G., Collinson, L.M., Baker, D.A., and Blackman, M.J. (2018). The actinomyosin motor drives malaria parasite red blood cell invasion but not egress. MBio *9*, 47.

Rackham, M.D., Brannigan, J.A., Rangachari, K., Meister, S., Wilkinson, A.J., Holder, A., Leatherbarrow, R.J., and Tate, E.W. (2014). Design and synthesis of high affinity inhibitors of *Plasmodium falciparum* and *Plasmodium vivax* N-myristoyltransferases directed by ligand efficiency dependent lipophilicity (LELP). J. Med. Chem. *57*, 2773–2788.

Rackham, M.D., Yu, Z., Brannigan, J.A., Heal, W.P., Paape, D., Barker, K.V., Wilkinson, A.J., Smith, D.F., Leatherbarrow, R.J., and Tate, E.W. (2015). Discovery of high affinity inhibitors of *Leishmania donovani* N-myristoyltransferase. MedChemComm *6*, 1761–1766.

Rees-Channer, R.R., Martin, S.R., Green, J.L., Bowyer, P.W., Grainger, M., Molloy, J.E., and Holder, A. (2006). Dual acylation of the 45 kDa

gliding-associated protein (GAP45) in *Plasmodium falciparum* merozoites. Mol. Biochem. Parasitol. *149*, 113–116.

Ritzefeld, M., Wright, M.H., and Tate, E.W. (2017). New developments in probing and targeting protein acylation in malaria, leishmaniasis and African sleeping sickness. Parasitology *8*, 1–18.

Rosario, V. (1981). Cloning of naturally occurring mixed infections of malaria parasites. Science *212*, 1037–1038.

Schlott, A.C., Holder, A.A., and Tate, E.W. (2018). N-Myristoylation as a drug target in malaria: exploring the role of N-myristoyltransferase substrates in inhibitor mode of action. ACS Infect. Dis. *4*, 449–457.

Schrodinger. (2014). The PyMOL Molecular Graphics System. Version 1.7 (Schrodinger LLC).

Slaughter, A., Jurado, K.A., Deng, N., Feng, L., Kessl, J.J., Shkriabai, N., Larue, R.C., Fadel, H.J., Patel, P.A., Jena, N., et al. (2014). The mechanism of H171T resistance reveals the importance of Nδ-protonated His171 for the binding of allosteric inhibitor BI-D to HIV-1 integrase. Retrovirology *11*, 161.

Smilkstein, M., Sriwilaijaroen, N., Kelly, J.X., Wilairat, P., and Riscoe, M. (2004). Simple and inexpensive fluorescence-based technique for high-throughput antimalarial drug screening. Antimicrob. Agents Chemother. *48*, 1803–1806.

Sutherland, C.J., Tanomsing, N., Nolder, D., Oguike, M., Jennison, C., Pukrittayakamee, S., Dolecek, C., Hien, T.T., do Rosário, V.E., Arez, A.P., et al. (2010). Two nonrecombining sympatric forms of the human malaria parasite *Plasmodium ovale* occur globally. J. Infect. Dis. *201*, 1544–1550.

Tate, E.W., Bell, A.S., Rackham, M.D., and Wright, M.H. (2013). N-Myristoyltransferase as a potential drug target in malaria and leishmaniasis. Parasitology *141*, 37–49.

Thomas, J.A., Collins, C.R., Das, S., Hackett, F., Graindorge, A., Bell, D., Deu, E., and Blackman, M.J. (2016). Development and application of a simple plaque assay for the human malaria parasite *Plasmodium falciparum*. PLoS One *11*, e0157873.

Trager, W., and Jensen, J.B. (2005). Human malaria parasites in continuous culture. J. Parasitol. *91*, 484–486.

Vagin, A., and Lebedev, A. (2015). MoRDa, an automatic molecular replacement pipeline. Acta Crystallogr. Sect. A *A71*. https://doi.org/10.1107/s2053273315099672.

Wagner, J.C., Platt, R.J., Goldfless, S.J., Zhang, F., and Niles, J.C. (2014). Efficient CRISPR-Cas9-mediated genome editing in *Plasmodium falciparum*. Nat. Methods *11*, 915–918.

World Health Organization (2017). World Malaria Report 2017.

Wright, M.H., Clough, B., Rackham, M.D., Rangachari, K., Brannigan, J.A., Grainger, M., Moss, D.K., Bottrill, A.R., Heal, W.P., Broncel, M., et al. (2014). Validation of N-myristoyltransferase as an antimalarial drug target using an integrated chemical biology approach. Nat. Chem. *6*, 112–121.

Wright, M.H., Paape, D., Price, H.P., Smith, D.F., and Tate, E.W. (2016). Global profiling and inhibition of protein lipidation in vector and host stages of the sleeping sickness parasite *Trypanosoma brucei*. ACS Infect. Dis. *2*, 427–441.

Wright, M.H., Paape, D., Storck, E.M., Serwa, R.A., Smith, D.F., and Tate, E.W. (2015). Global analysis of protein N-myristoylation and exploration of N-myristoyltransferase as a drug target in the neglected human pathogen *Leishmania donovani*. Chem. Biol. *22*, 342–354.

Wu, Y., Gao, F., Qi, J., Bi, Y., Fu, L., Mohan, S., Chen, Y., Li, X., Pinto, B.M., Vavricka, C.J., et al. (2016). Resistance to mutant group 2 influenza virus neuraminidases of an oseltamivir-zanamivir hybrid inhibitor. J. Virol. *90*, 10693–10700.

Yeoh, S., O'Donnell, R.A., Koussis, K., Dluzewski, A.R., Ansell, K.H., Osborne, S.A., Hackett, F., Withers-Martinez, C., Mitchell, G.H., Bannister, L.H., et al. (2007). Subcellular discharge of a serine protease mediates release of invasive malaria parasites from host erythrocytes. Cell *131*, 1072–1083.

Yu, Z., Brannigan, J.A., Moss, D.K., Brzozowski, A.M., Wilkinson, A.J., Holder, A., Tate, E.W., and Leatherbarrow, R.J. (2012). Design and synthesis of inhibitors of *Plasmodium falciparum* N-myristoyltransferase, a promising target for anti-malarial drug discovery. J. Med. Chem. *55*, 8879–8890.

Yu, Z., Brannigan, J.A., Rangachari, K., Heal, W.P., Wilkinson, A.J., Holder, A., Leatherbarrow, R.J., and Tate, E.W. (2015). Discovery of pyridyl-based inhibitors of *Plasmodium falciparum* N-myristoyltransferase. MedChemComm *6*, 1767–1772.

Zhang, C., Xiao, B., Jiang, Y., Zhao, Y., Li, Z., Gao, H., Ling, Y., Wei, J., Li, S., Lu, M., et al. (2014). Efficient editing of malaria parasite genome using the CRISPR/Cas9 system. MBio *5*, e01414.

# STAR★METHODS

## KEY RESOURCES TABLE

| REAGENT or RESOURCE | SOURCE | IDENTIFIER |
|---|---|---|
| Antibodies | | |
| Rabbit anti GAP45 antibody used in Figure S2B. | Rabbit anti GAP45 antibody has been validated in: Rees-Channer et al., 2006 | N/A |
| Experimental Models: Cell Lines | | |
| Dd2 Plasmodium falciparum | Clone B2 from David Fidock's Lab - recloned from the Dd2 parental line by limiting dilution. | N/A |
| 3D7 Plasmodium falciparum | parasite line originating from National Institute for Medical Research. | N/A |
| Chemicals, Peptides, and Recombinant Proteins | | |
| YnMyr, AzTB | Heal et al., 2011 | N/A |
| DDD85646 | Frearson et al., 2010 | N/A |
| IMP-0964 | Yu et al., 2012 | N/A |
| IMP-0856 | Yu et al., 2012 | N/A |
| IMP-0320 | Wright et al., 2014 | N/A |
| IMP-0917 | Mousnier et al., 2018 | N/A |
| IMP-1002 | synthesis route in STAR Methods section of this study | N/A |
| Myristoyl coenzyme A lithium salt | Sigma Aldrich | Cat# M4414 |
| Hoechst 33342 viability stain | New England biolabs, | Cat# 4082S |
| SYBR Green | Life Technologies | Cat# S7563 |
| HsSrc_15AA_peptide | Synthesied at the Francis Crick Institute Nicola O'Reilly and Ganka Bineva-Todd | N/A |
| PvNMT G386E and PvNMT WT enzyme | expressed and purified by Alexandra R. Reers at Seattle Structural Genomics Center for Infectious Disease (SSGCID); Center for Global Infectious Disease Research, Seattle Children's Research Institute | N/A |
| CPM | Sigma Aldrich | Cat# C1484 |
| Deposited Data | | |
| Crystal structure deposition | The PDB files that support the findings of this study have been deposited in Protein Data Bank | accession codes 6MAY (PvNMT G386E + MyrCoA), 6MAZ (PvNMT G386E, + MyrCoA + IMP-0366) 6MB0 (PvNMT G386E + MyrCoA + IMP-1002), and 6MB1 (PvNMT WT + MyrCoA + IMP-1002). |
| Crystal structure previously publsihed | The PDB files used in this study from previously published work | 5O6H (PvNMT WT + MyrCoA + IMP-0917), 2YND (PvNMT WT + MyrCoA + IMP-0366), 2YNE PvNMT WT + MyrCoA + IMP-0320), 4UFX (PvNMT WT + MyrCoA + IMP-0856) |
| Oligonucleotides | | |
| G386E-guide-01_F (Figure S3) | Sigma Aldrich Oligos | sequence: ATTGTTAAAATTTGGA GAAGGAGA |
| G386E-guide-01_R (Figure S3) | Sigma Aldrich Oligos | sequence: AAACTCTCCTTCTCC AAATTTTAA |
| G386E-guide-01_F (Figure S3) | Sigma Aldrich Oligos | sequence: ATTGTTTTAATGCCTTAG AAGTAA |

(*Continued on next page*)

| *Continued* | | |
|---|---|---|
| REAGENT or RESOURCE | SOURCE | IDENTIFIER |
| G386E-guide-01_R (Figure S3) | Sigma Aldrich Oligos | sequence: AAACTTACTTCTAAGGC ATTAAAA |
| P1 (Figure S3) | Sigma Aldrich Oligos | sequence: TATGGCAAGCTATATATA CAGCAGG |
| P2 (Figure S3) | Sigma Aldrich Oligos | sequence: CCTCAGACTATATAACA TTAGAATG |
| Software and Algorithms | | |
| Flow cytometry data collection in Figure 3B | FACSDiva software v8.0.1 | N/A |
| Flow cytometry data analysis of Figure 3B | FlowJo 10.3. | N/A |
| CPM assay in Figures 4A and 6B | EnVision Workstation version 1.13.3009.1409 | N/A |
| EC50 determination in Figures 4B and 6A | FLUOStar Omega Plate reader software was used from BMG Labtech | N/A |
| SPR data in Figure 6C | collected using Biacore T200 Evaluation Software v2.0 | N/A |
| Immunofluorescence images Figure S2B | collected using the Nikon's NIS Elements imaging software | N/A |
| Analysis of data from Figures 2, 3A, 3D, 4, 6, S2A, S2C, S4B, S5A, and S5C | Prism 7 GraphPad and Microsoft Excel | N/A |
| Chemical structure in Figures 1A and S1. | ChemDraw Professional 17.0 | N/A |
| Crystallography in Figures 5, S6A, and S6B, Table S1 | The structures were refined in Phenix, with manual model building in Coot. Quality of the models was assessed with MolProbity. Figures were generated using Pymol and CCP4MG. SPR data (Figure 6C) was analyzed using Biacore T200 Evaluation Software v2.0. | N/A |

## CONTACT FOR REAGENT AND RESOURCE SHARING

Further information and requests for resources and reagents should be directed to and will be fulfilled by the Lead Contact Prof. Edward W. Tate, e.tate@imperial.ac.uk.

## EXPERIMENTAL MODEL AND SUBJECT DETAILS

### Parasite Culture

*P. falciparum* 3D7 parasites were cultured *in vitro* in RPMI 1640 medium containing 0.5 % w/v Albumax II at 2-5 % hematocrit as described (Trager and Jensen, 2005). Parasites cultures were gassed with 90 % $N_2$, 5 % $CO_2$ and 5 % $O_2$ and incubated at 37°C. Parasites were synchronized using 70 % Percoll gradients to purify schizont stages with a subsequent reinvasion followed by sorbitol treatment as described (Knuepfer et al., 2017).

## METHOD DETAILS

### Synthesis Route of IMP-1002

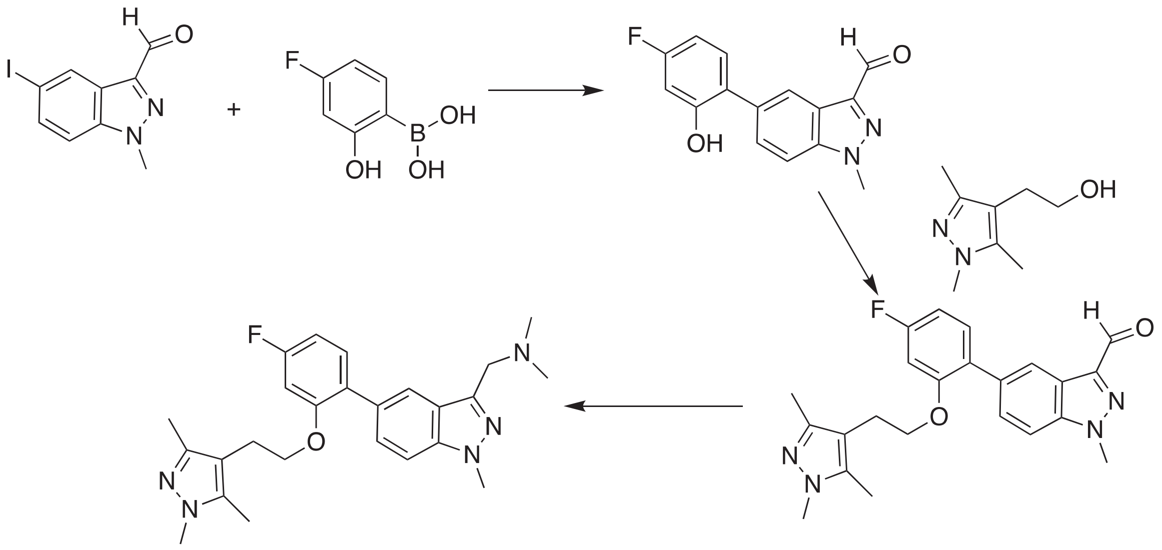


***Step 1***

A solution of 5-iodo-1-methyl-1*H*-indazole-3-carbaldehyde (500 mg, 1.7mmol) was dissolved in dioxane (5 mL) and treated with 4-fluoro-2-hydroxybenzene boronic acid (360 mg, 2.2 mmol) and tetrakis(triphenylphosphine) palladium(0) (20 mg), followed by a solution of potassium phosphate (560 mg, 2.6 mmol) in water (1 mL). The reaction mixture was heated under reflux for 2 h, cooled to room temperature and evaporated under reduced pressure. The residue was partitioned between EtOAc (20 mL) and saturated sodium bicarbonate solution (20 mL). The organic phase was dried over $Na_2SO_{4,}$ concentrated under reduced pressure and the crude product purified by flash column chromatography by elution with DCM/EtOAc (100:0, then 95:5) to give 5-(4-fluoro-2-hydroxyphenyl)-1methyl-1*H*-indazole-3-carbaldehyde as a colourless solid (450mg, yield: 95%); mp 212-214°C. $^1$H NMR ($CD_3OD$, δ, ppm) 10.15 (s, 1H), 8.30 (d, *J* = 1.3 Hz, 1H), 7.73 (d, *J* = 1.5 Hz, 1H), 7.69 (s, 1H), 7.32 (dd, *J* = 9.2, 6.7 Hz, 1H), 6.74 – 6.64 (m, 2H), 4.62 (s, 1H), 4.24 (s, 3H).

***Step 2***

A solution of 5-(4-fluoro-2-hydroxyphenyl)-1-methyl-1*H*-indazole-3-carbaldehyde (135 mg; 0.5 mmol) and triphenylphosphine (262 mg; 1.0 mmol) in THF (4 mL) was treated with a solution of 2-(1,3,5-trimethyl-1*H*-pyrazol-4-yl)ethanol (154 mg, 1.0 mmol) in THF (3 mL). The reaction mixture was cooled to 0°C before being treated with di-isopropyl azodicarboxylate (291 mL, 1.48 mmol). The reaction mixture was allowed to warm to room temperature, stirred overnight and concentrated under reduced pressure. The crude product was purified by column chromatography by elution with hexane/IPA (gradient 80:20 to 70:30) to give a mixture of the desired product and unreacted alcohol. The crude product was further purified by elution with DMC/methanol (97:3) to give 5-(4-fluoro-2-(2-(1,3,5-trimethyl-1H-pyrazol-4-yl)ethoxy)phenyl)-1-methyl-1H-indazole-3-carbaldehyde as a colourless oil (86 mg, 42%). $^1$H NMR ($CDCl_3$, δ, ppm) 10.22 (s, 1H), 8.37 (d, *J* = 1.5 Hz, 1H), 7.57 (dd, *J* = 8.8, 1.5 Hz, 1H), 7.46 (d, *J* = 8.7 Hz, 1H), 7.29 (dd, *J* = 8.4, 6.8 Hz, 1H), 6.72 (td, *J* = 8.3, 2.5 Hz, 1H), 6.67 (dd, *J* = 11.1, 2.4 Hz, 1H), 4.21 (s, 3H), 3.94 (t, *J* = 7.2 Hz, 2H), 3.62 (s, 3H), 2.74 (t, *J* = 7.1 Hz, 2H), 2.04 (s, 3H), 1.95 (s, 3H).

***Step 3***

A solution of 5-(4-fluoro-2-(2-(1,3,5-trimethyl-1H-pyrazol-4-yl)ethoxy)phenyl)-1-methyl-1H-indazole-3-carbaldehyde (43 mg, 0.11 mmol) in THF (3 mL) was treated with a solution of dimethylamine in THF (2 M, 160 mL 0.33 mmol), followed by glacial acetic acid (145 mL, 0.66 mmol). The solution was stirred at room temperature for 10 min before addition of solid sodium triacetoxy-borohydride (70 mg, 0.33 mmol) and DCE (2 mL). The reaction mixture was stirred at room temperature for 18 h before being quenched with sodium carbonate solution (2 M, 5 mL). DCM (20 mL) was added and the layers were separated. The organic extract was dried ($Na_2SO_4$) and concentrated under reduced pressure. The crude product was purified by column chromatography by elution with EtOAc/diethylamine (95:5) to provide **1**-(5-(4-fluoro-2-(2-(1,3,5-trimethyl-1H-pyrazol-4-yl)ethoxy)phenyl)-1-methyl-1H-indazol-3-yl)-N,N-dimethylmethanamine as a colourless oil (13.5 mg, 31%). LC-MS Rt = 11.6min, $MH^+$ 436; $^1$H NMR ($CDCl_3$, δ, ppm) 7.88 (d, *J* = 1.8 Hz, 1H), 7.48 (dd, *J* = 8.7, 1.5 Hz, 1H), 7.35 (d, *J* = 8.7 Hz, 1H), 7.33 – 7.26 (m, 1H), 6.73 (td, *J* = 8.3, 2.5 Hz, 1H), 6.67 (dd, *J* = 10.8, 2.4 Hz, 1H), 4.08 (s, 3H), 3.93 (t, *J* = 7.2 Hz, 2H), 3.83 (s, 2H), 3.63 (s, 3H), 2.75 (t, *J* = 7.3 Hz, 2H), 2.33 (s, 6H), 2.08

(s, 3H), 1.92 (s, 3H); $^{13}C$ NMR ($CDCl_3$, δ, ppm) 162.76 (d, $J$ = 245.2 Hz), 156.81 (d, $J$ = 9.9 Hz), 145.75, 142.38, 140.07, 136.91, 131.89 (d, $J$ = 10.0 Hz), 130.14, 128.66, 127.19, 123.46, 121.30, 111.62, 108.19, 107.20 (d, $J$ = 21.0 Hz), 100.39 (d, $J$ = 25.8 Hz), 68.63, 55.81, 45.59, 35.75, 35.41, 23.66, 11.71, 9.34; ESI HRMS, found 436.2510 ($C_{25}H_{31}FN_5O$, $[M + H]^+$, requires 436.2513).

### Indirect Immunofluorescence Assay (IFA)

For IFA, slides were air dried, fixed in 4 % paraformaldehyde (PFA) in PBS for 10–20 min, permeabilized in 0.1 % Triton X-100 in PBS for 10 min, and blocked with 3 % BSA in PBS for at least 30 min at 4°C. GAP45 detection was achieved using a primary rabbit anti-GAP45-antibody (Rees-Channer et al., 2006). Slides were mounted in ProLong® Gold Antifade mounting medium containing DAPI (4',6-diamidino-2-phenylindole), and viewed on a Nikon Eclipse Ni-E imaging system with a Hamamatsu Orca-flash 4.0 digital camera and a Plan apo λ 100x/1.45 oil immersion objective. Images were captured using Nikon's NIS-Elements imaging software generating Z-stack images of individual parasites, using deconvolution options and exporting the image as a tiff file, which was further edited using Adobe Photoshop.

### Drug Treatment and $EC_{50}$ Determination Using the SYBR Green Assay

Aliquots of 100 μL ring-stage parasites (1–6 h post invasion [PI]) were diluted in 96-well flat bottom black plates to 0.3 % parasitemia and 2 % hematocrit. Inhibitors were added in doubling dilutions ranging from 7.8 nM – 5 μM for DDD85646 and 0.54 nM – 400 nM for IMP-1002 for the initial $EC_{50}$ determination in 3D7 in a final volume of 100 μL. For the $EC_{50}$ of PfNMT[G386E] compared to PfNMT[WT] a range from 0.2 nM – 10 μM for IMP-1002 and DDD85646 and 0.6 nM - 40 μM for IMP-0964 and IMP-0320 was used. The final DMSO percentage of 0.08% did not interfere with parasite development determined by Giemsa-stained thin blood smears. Cultures were incubated in gassed humidity chambers (90 % $N_2$, 5 % $CO_2$ and 5 % $O_2$) at 37°C for a further 96 h to increase the dynamic range and processed as described (Green et al., 2015). After 96 h, parasites were lysed and DNA was stained by adding to each well 25 μl lysis buffer (20 mM Tris-HCl, pH 8.0, 2 mM EDTA, pH 8.0, 1.6 % Triton X-100, 0.16 % saponin, containing 0.1% SYBR Green [Life Technologies]) (final SYBR Green concentration of 0.02%) (Smilkstein et al., 2004). Plates were incubated for 1 h in the dark at room temperature (RT) before measurements on a FLUOStar Omega Plate reader (BMG Labtech) with excitation and emission filters of 485 nm and 520 nm, respectively. $EC_{50}$ values were calculated from a four-parameter logistical fit of the data using Prism software (GraphPad Software, Inc.), with subtraction of the background signal from uninfected RBC. When comparing the $EC_{50}$ of two parasite lines, Prism software was used to fit and constrain $EC_{50}$ curves at the top and bottom to improve plateau definition through a shared value of all data sets.

### Selection of IMP-1002-Resistant *P. falciparum* Dd2 Parasites Using a Single-Step Strategy

Prior to selection, genomic DNA was harvested and a frozen stock of the parental Dd2 parasite line was generated. Selection was initiated with three wells of $10^6$ parasite inocula and three wells of $10^7$ parasite inocula. A final concentration of 120 nM IMP-1002 (3.4 x the 35 nM $EC_{50}$) was added to a predominantly ring stage culture. The culture was fed daily with drug-containing complete medium for six days and every other day thereafter. The parasitemia was monitored and kept below 10 % over the next days to prevent overgrowth. Fresh RBCs (equivalent to 0.5 % hematocrit) were added at day 7. At day 14, and weekly thereafter, the cultures were passaged (¾ cultured cells were replaced ¼ with fresh RBC and new medium) to prevent lysis. Drug pressure was maintained for 60 days.

### NMT gDNA Amplification and Sequencing from IMP-1002 Resistant *P. falciparum*

The genomic DNA sequence of the *nmt* gene in *P. falciparum* 3D7 and Dd2 is identical (www.PlasmoDB.org) and was used for primer design. Following PCR amplification, the product was sequenced and cloned into pGEM-T Easy vector after ligation of a 3'-A overhang using Amplitaq polymerase to improve the sequence quality. Sequence data were analyzed using DNASTAR SeqMAN Pro and a consensus sequence for each of the parasite lines was fed into CLC Sequence Viewer 7 to create a nucleotide alignment. The nucleotide sequence was also translated into protein, which was aligned to the WT sequence using CLC Sequence Viewer 7.

### Cloning of Constructs and Transfection of 3D7 *P. falciparum*

The construct to generate the G386E NMT mutation was generated using Gene Art and provided on a pMK-RQ kanamycin resistance plasmid. The two independent guides were designed using protospacer.com and cloned into the pDC2-cam-Cas9-U6-hDHFRyFCU-plasmid (Knuepfer et al., 2017; Lim et al., 2016; Macpherson and Scherf, 2015). Guide and rescue plasmids were paired and ethanol precipitated prior to transfection. Asexual blood-stage cultures of *P. falciparum* clone 3D7 were maintained *in vitro* and synchronized according to standard procedures (Gerold et al., 1996; Yeoh et al., 2007). Transfection DNA was added to mature schizonts, enriched from highly synchronous cultures using Percoll (GE Healthcare) gradients as described (Harris et al., 2005). Parasites were transfected by electroporation using the Amaxa 4D electroporator (Lonza) and the P3 Primary cell 4D Nucleofector X Kit L (Lonza) and program FP158, as recently described for *P. knowlesi* (Moon et al., 2013). Electroporations were performed with 60 μg of linearized rescue plasmid and 20 μg of the CRISPR/Cas9 plasmid carrying the respective guide RNA. Selections were carried out as recently described (Knuepfer et al., 2017). Parasites were cultured in the presence of 2.5 nM WR99210 drug pressure for five days to select for parasites with the Cas9/guide plasmid. Transfected parasites reappeared after 17 days. After 29 days, positive selection with 1 μM ancotil was initiated to minimize the existence of excess Cas9/guide plasmid. Transfected parasites were cloned by limiting

dilution after 37 days (Rosario, 1981) and screened for single parasites with a plaque assay (Thomas et al., 2016). Individual clones were screened for integration by PCR amplification and digestion with BglII (guide 1 transfection) and XhoI (guide 2 transfection).

### Parasite Growth Assay

Analysis of the growth of WT and two NMT[G386E] parasite clones over eight generations was performed by first equalizing the starting parasitemia (0.67 % WT, 0.73 % Clone 1 and 0.61 % Clone 2). Three biological replicates were generated in three different blood dilutions with three technical replicates each. WT parasitemia was determined following Giemsa staining after each cycle and used to determine the dilution factor to dilute all three lines down to 0.8%. Parasitemia was measured after each cycle using flow cytometry and Hoechst-stained parasites. Multiple t-tests were performed for each generation (two-stage linear step-up procedure of Benjamini, Krieger and Yekutieli, with false-discovery rate = 1 %).

### Metabolic Tagging of Parasites and CuAAC Labelling

Synchronized populations of stage-specific parasites were labelled with 25 μM of YnMyr for the specified length of time. Parasites were then lysed in 0.15 % saponin for 10 min on ice. The pellet was washed until the supernatant was colorless and then stored at -80°C. Thawed parasite pellets were extracted in 1 % Triton X-100, 0.1 % SDS in PBS on ice for 20 min with 1 min sonication. The protein concentration of the lysate was measured by BCA assay and adjusted to 1 mg/mL with PBS. The capture reagent was 'clicked' (100 μM capture reagent AzRB2, 1 mM $CuSO_4$, 1 mM TCEP, 100 μM TBTA), the reaction was quenched with EDTA, and then precipitated proteins were washed with ice cold methanol as described previously (Heal et al., 2011; Wright et al., 2015). Proteins were re-dissolved in 2 % SDS, 10 mM DTT, in PBS at 1 mg/mL and heated for 7 min at 95°C in sample loading buffer prior to SDS-PAGE.

### Base Treatment to Cleave the Esters of GPI-Anchored Proteins

Following the click reaction, the protein solution in PBS containing 2 % SDS, 10 mM DTT was treated with 0.2 M NaOH for 1 h at room temperature with vortexing. Samples were neutralized with an equivalent volume and concentration of HCl and diluted with PBS to 1 mg/mL prior to gel analysis.

### Flow Cytometry

Synchronized parasites were incubated with DMSO or NMTi and samples were fixed in 2% PFA, 0.2 % glutaraldehyde for 1 hour at 45 h PI. Subsequently samples were washed in PBS and labelled with 1:500 of 10 mg/mL Hoechst 33342 for 10 min with a subsequent wash in PBS. For analysis, a Flow Cytometry CL1 Fortessa D Analyzer from BD with FACSDiva software v8.0.1 was used with the 450-50 filter counting 50,000 RBC per sample. Data were analyzed using FlowJo LLC 2006-2015 to determine the median fluorescence intensity (MFI) of each sample after gating out the RBC population using an RBC only sample as a control. The MFI of each sample was divided by the MFI of a control sample of synchronized rings with a known MFI of one nucleus.

### Cloning, Expression and Purification of PvNMT

The purification method has been adapted from earlier work (Bryan et al., 2011; Choi et al., 2011). A region of the PvNMT gene encoding residues 27-410 and tagged at the N-terminus with a hexaHis sequence was cloned into a pET11a expression vector. The G386E mutation was generated using QuickChange II mutagenesis by exchanging base pairs 1079 and 1080 of the PvNMT open reading frame from GC to AA, which changes glycine at position 386 to glutamic acid. The constructs were confirmed by sequencing and the plasmid DNAs were used to transform Rosetta-gami (DE3) *E. coli* competent cells, using ampicillin for pET11a selection and chloramphenicol for the pRARE2 plasmid present in the Rosetta-gami DE3 cells to supply the tRNAs for rare codons used to codon-optimize PvNMT. PvNMT[WT] and PvNMT[G386E] were expressed following inoculation of a single colony overnight at 37°C in 5 mL Lysogeny Broth. The PvNMT[WT] protein was expressed in 8 x 500 mL auto-induction medium (92 % ZY, 0.1 % 1M $MgSO_4$, 0.1 % 1000x Trace Metals, 5 % 20xNPS, 2 % 50x 5052) (2x 500mL for small scale) and the PvNMT[G386E] protein in 19 L due to low yield. Auto-induction medium was combined with appropriate antibiotics and incubated with 1:100 dilution factor from the overnight culture at 37 C for 4 h. Temperature was then reduced to 18°C for 18-22 h. Bacterial cells were harvested by centrifugation and pellets from 4 L (for the WT protein) re-suspended in 180 mL of lysis buffer (20 mM HEPES, 5 % glycerol, 10 mM imidazole, 0.5 % CHAPS, 21 mM $MgCl_2$, 1 mM DTT and Complete Protease Inhibitor Cocktail EDTA-free). For the PvNMT[G386E] protein cells from the 15 L were resuspended in 2x 300 mL, sonicated separately and then combined prior to application to the column. They were lysed using a BRANSON 550 sonication probe (20 % strength, 5 sec on 10 sec off for 10 min, 15 min total of sonication). The lysate was cleared by centrifugation at 48,384*g* RCF for 60 min at 4°C in a 25.50 JA rotor.

For large scale preparations the lysate was filtered through a 0.45 μM syringe filter and applied to a HisTRAP FF 5 mL column at 4°C and eluted with a linear gradient over 15 column volumes with 20 mM HEPES, 0.3 M NaCl, 5 % glycerol, 0.5 M imidazole; pH 7.0. Fractions containing the target protein were pooled and concentrated to about 2-3 mL using 10 kDa-cut off Pall centrifuge concentrators.

For small scale preps the lysate was incubated in batch with 20 mL of Ni-NTA Agarose beads (QIAGEN) equilibrated with lysis buffer in a 500 mL Duran bottle at 4°C for 1 hour. The beads were washed using a re-usable glass Fritted Funnel column and a pressure pump with 20x bead volume of wash buffer using a pressure pump (20 mM HEPES, 5 % glycerol, 30 mM imidazole, 0.5 % CHAPS, 21 mM $MgCl_2$). The bound protein was eluted from the beads using 3x bead volume of elution buffer through gravity

(0.3 M NaCl, 20 mM HEPES, 5 % glycerol, 0.5 M imidazole; pH 7.0). Eluted proteins were analyzed using instant blue (Expedeon Prod # ISB1L). Nickel elution was filtered and concentrated using Centricon Plus 70 with a 30kDa cut-off.

For both small and large scale preparations concentrated protein was diluted 100-fold with anion exchange buffer (20 mM Tris–HCl, 1 mM TCEP; pH 8.9) and applied directly to a HiTRAP Q HP 5 mL column (1 mL column for small scale) at 2 mL/min. Unbound sample was washed away with 5-8 column volumes (CV) at 2 mL/min for the 1 mL column and 3 mL/min for the 5mL column and eluted with 2 %-50 % of the elution buffer over 15 CV at 0.5 mL/min for the 1 mL column and 1 mL/min for the 5mL column. The 2 mL fraction (5 mL column) or 0.5 mL fractions (1 mL column) were analysed by applying 10 μL to an SDS PAGE gel and visualized using instant blue. For protein for crystallography, the His tag was cleaved from the PvNMT[WT] and PvNMT[G386E] proteins via 3C protease (His-MBP-3C) at a 1:50 protease: protein ratio overnight at 4°C in dialysis buffer (25 mM HEPES pH 7.5, 500 mM NaCl, 5 % (v/v) glycerol, 1 mM TCEP, and 0.025 % sodium azide). NMT was recovered by applying the dialysate to a second IMAC column. Fractions containing target protein were pooled and concentrated to 70 μL for the small scale or 4 mL for large scale and loaded onto Superdex 75 10/300 or 26/600 columns and eluted with size exclusion buffer (0.3 M NaCl, 20 mM HEPES, 5 %(v/v) glycerol, 1 mM TCEP; pH 7.0). Fractions were analyzed on an SDS-PAGE gel and fractions containing the target protein were either pooled in the case of PvNMT[WT] or because of limited material kept separate for the PvNMT[G386E] protein. Fractions were concentrated and flash frozen and stored at – 80°C until further use.

For small scale expression the purified protein was detected by Western blotting, using the anti-PvNMT antibody (PvNMT protein made by Jim Brannigan at York University and antibodies raised by David Moss, MRC National Institute for Medical Research). For Western blots, proteins were transferred from the gel to nitrocellulose membrane with the iBlot™ Transfer System. Following blocking overnight at 4°C, membranes were incubated with primary antibody for 1 h at room temperature in blocking solution and incubated with secondary antibody (goat-anti-rabbit/rat/mouse IgG-HRP, Invitrogen 1:2500) for 1 h in blocking solution. Detection was carried out using GE Healthcare ECL Western Blotting Detection reagents or BioRad Clarity Western ECL Substrate and visualized on a ChemiDoc MP BIO-RAD Imaging System.

### CPM-Based PvNMT $IC_{50}$ Determination

To measure the activity of the purified PvNMT[WT] and PvNMT[G386E], an assay was used with thiol-selective fluorescent dye (CPM) to monitor the formation of coenzyme A. The assay was performed as published in (Goncalves et al., 2012a). Reagents were prepared in 20 mM sodium phosphate, 0.5 mM EDTA, 0.1 % Triton X-100 at pH 7.90-7.95 with the corresponding DMSO concentrations. First, 10 μL of a 10 % DMSO/water (v/v) solution containing a dilution series of NMT inhibitor, 50 μL of NMT in 1 % DMSO (final concentration of 6.3 nM), and 25 μL of myristoyl–CoA solution in 1 % DMSO (4 μM final concentration), were combined in Black microplate 96-well non-sterile flat bottom polypropylene plates (655209). The reaction was started by adding 25 μL of CPM and a 15-residue HsSrc peptide solution in 5 % DMSO (final concentration CPM 8 μM, HsSrc peptide 4 μM). For the continuous assay the fluorescence intensity was monitored over 45 min at 0.5-min intervals (excitation at 380 nm, emission at 470 nm) at 25°C using the EnVision Xcite Plate Reader (exported with EnVision Workstation version 1.13.3009.1409). For the quenched assay the reaction was stopped after 30 min at which point the initial reaction rate was still in the linear range by adding 60 μL of the Quench Solution (50 mM acetic acid, 50 mM sodium acetate, pH 4.74-4.78).

### Thermal Shift Assay

Purified recombinant PvNMT[WT] and PvNMT[G386E] were diluted to 0.1 mg/mL in PBS buffer containing 10% glycerol and 1:1000 dilution of Sypro Orange in the presence or absence of 4 μM myristoylCoA. Test compounds were diluted to 500 μM in DMSO, and 0.5 μL of diluted compound was added to 49 ul of enzyme in a 96-well plate resulting in two technical replicates with a final volume of 20 μL and 1% (final) DMSO with a compound concentration of 5 μM. Each compound was tested twice to give two experimental replicates, each with two technical replicates. Reference thermal melting temperatures (Tms) were obtained using 1% DMSO without any compound present. The plates were subjected to a temperature gradient from 300K to 348K at a rate of 1K/min using a FluoDia fluorescent plate reader. Fluorescence data were acquired with an excitation filter of 486nm and an emission filter at 610nm, and the raw data were exported to Prism or Excel for analysis. Data were processed to identify the fluorescence maxima and minima, and the midpoints of melting curves were determined by fitting to a Boltzmann equation, where Tm is the temperature corresponding to the half maximum response. $(Y=y_{min} + (y_{max}-y_{min})/ (1+\exp((Tm-T)/slope))$. Results were expressed as ΔTm in °C, where ΔTm is the difference between Tm in the presence of compound subtracted from that in the reference without compound present.

### Surface Plasmon Resonance (SPR) Assay

The surface plasmon resonance experiments were performed using a Biacore T200 (GE Healthcare) equipped with research-grade CM5 sensor chips. PvNMT (5.14 mg/mL, 45.0 kDa, 20 mM HEPES pH 7.0, 300 mM NaCl, 5 % glycerol, 1 mM TCEP) was diluted to 0.0514 mg/mL in 10 mM sodium acetate pH 6.0. PvNMT[G386E] (0.95 mg/mL, 47.3 kDa, 20 mM HEPES pH 7.0, 300 mM NaCl, 5 % glycerol, 1 mM TCEP) was diluted to 0.0428 mg/mL in 10 mM sodium acetate pH 6.0. Each ligand was immobilized onto separate flow cells using amine-coupling chemistry. The surfaces of all four flow cells were activated for 7 min with a 1:1 mixture of 0.1 M NHS (N-hydroxysuccinimide) and 0.1 M EDC (3-(N,N-dimethylamino) propyl-N-ethylcarbodiimide) at a flow rate of 10 μL/min. PvNMT was immobilized at a density of 900 - 1100 RU onto flow cell 3, and PvNMT[G386E] at 600-1700 RU onto flow cell 4. Flow cell 1 was left blank to serve as a reference surface. All four surfaces were blocked with a 7 min injection of 1 M ethanolamine, pH 8.0. IMP-0320 was used as a positive control in the assay as it has been extensively used by GSK for routine NMT SPR assays.

The analytes (IMP compounds [400-550 Da] dissolved in DMSO to a concentration of 500 μM,) were serially diluted 1:3 in DMSO, and then 1-3 μL of each dilution was dissolved in an assay buffer of 10 mM HEPES pH 7.4, 150 mM NaCl, 0.005 % Tween 20, 4 μM myristoylCoA to a final DMSO concentration of 1 %. To collect binding affinity data, the analyte concentrations (0-0.4 μM except from DDD85646: 0-1 μM) were injected sequentially over all four flow cells at a flow rate of 30 μL/min and at a temperature of 25°C. The complexes formed were allowed to associate for 200-700 sec and then dissociate for 600-2400 sec, dependent on the analyte kinetics observed. The surfaces were washed with an injection of 50 % DMSO between each analyte injection. The data were analyzed within Biacore T200 Evaluation Software v2.0.

Equilibrium sensorgram values were exported and the offset set to zero before fitting to a global 'One site – Specific binding' nonlinear regression model (Motulsky and Brown, 2006) in GraphPad Prism 7. As differential binding of compounds to PvNMT [G386E] and PvNMT[WT] were of greatest interest, the ratio of $K_D$ PvNMT[G386E] to that of PvNMT [WT] was calculated for each compound dose response on the same chip within an experiment.

### Crystallography

PvNMT[WT] and PvNMT[G386E] were crystallized using the sitting drop vapor diffusion method at 7.8 mg/mL and 12.2 mg/mL respectively against a focused screen generated from a published literature condition (Yu et al., 2012). Drops contained 0.4 μL protein (supplemented with either 0.5 mM MyrCoA or 0.5 mM MyrCoA and 0.5 mM inhibitor) and 0.4 μL crystallant. Drops were equilibrated against 80 μL of the crystallant in Compact Jr crystallization trays (Rigaku Reagents) and incubated at 16°C. Large, rod-morphology crystals appeared between one and two weeks and were cryo-protected with 20-25 % (v/v) ethylene glycol supplemented with ligand(s) prior to vitrification in liquid nitrogen for X-ray data collection.

X-ray diffraction data was collected at the LS-CAT beamline 21-ID-F at the Advanced Photon Source. Collection information and processing statistics are reported in Supplemental Information appendix Table S1. The data were integrated with XDS and reduced with XSCALE (Kabsch, 2010). Data quality assessment and space group assignment were performed using POINTLESS (Evans, 2006). Structures were solved by molecular replacement using MoRDa (Vagin and Lebedev, 2015), using PvNMT chain A (PDB accession code 4B14) in the same crystal form as a search model. The structures were refined in Phenix (Adams et al., 2010), with manual model building in Coot (Emsley and Cowtan, 2004). Quality of the models was assessed with MolProbity (Chen et al., 2010). Figures were generated using Pymol (Schrodinger, 2014) and CCP4MG (McNicholas et al., 2011). Inhibitor omit maps (Figure S6A) were generated by complete removal of inhibitor atoms from fully refined structures and running an additional refinement cycle in Phenix. Coordinates and structure factors have been deposited in the Protein Data Bank under accession codes 6MAY, 6MAZ, 6MB0, and 6MB1.

## QUANTIFICATION AND STATISTICAL ANALYSIS

Data were analyzed using Prism 7.0 by GraphPad. Quantitative data are presented as mean ± standard deviation. Dose response curve fitting in Figures 2, 3A, 4A, 4B, 6A, 6B, and S2A, to calculate $IC_{50}$s $EC_{50}$, Hill coefficients and confidence intervals, was done using four-parameter [Inhibitor] vs. response. For KD determination through SPR in Figures 4C and 6C a 'One site – Specific binding' nonlinear regression model (Motulsky and Brown, 2006) was used. Replicate number and definition of n is indicated in figure legends. For Figure 3A an unpaired Welch T-test was performed not assuming equal SDs. For Figure S4B a multiple t-tests was performed for each parasite cycle (two-stage linear step-up procedure of Benjamini, Krieger and Yekutieli, with false-discovery rate = 1 %).

## DATA AND SOFTWARE AVAILABILITY

The PDB files that support the findings of this study have been deposited in Protein Data Bank under accession codes 6MAY, 6MAZ, 6MB0, and 6MB1.

Cell Chemical Biology, Volume *26*

# Supplemental Information

# Structure-Guided Identification of Resistance Breaking Antimalarial *N*-Myristoyltransferase Inhibitors

**Anja C. Schlott, Stephen Mayclin, Alexandra R. Reers, Olivia Coburn-Flynn, Andrew S. Bell, Judith Green, Ellen Knuepfer, David Charter, Roger Bonnert, Brice Campo, Jeremy Burrows, Sally Lyons-Abbott, Bart L. Staker, Chun-Wa Chung, Peter J. Myler, David A. Fidock, Edward W. Tate, and Anthony A. Holder**

# Supplementary Information for

A novel antimalarial resistance mechanism leads to structure-guided identification of

resistance-breaking Plasmodium N-myristoyltransferase inhibitors

**Author list in order of appearance on the paper:**


Anja C. Schlott, <anja.schlott@crick.ac.uk> Francis Crick Institute, 1 Midland Rd, London NW1 1AT, UK; Molecular Sciences Research Hub, Imperial College, White City Campus Wood Lane, London W12 0BZ, UK, ORCID: 0000-0002-7139-8699

Stephen Mayclin <stephen.mayclin@ucb.com>, Seattle Structural Genomics Center for Infectious Disease (SSGCID), Seattle, WA 98109, - UCB Pharma, 7869 NE Day Road West, Bainbridge Island, WA 98110, ORCID: 0000-0002-1600-4554

Alexandra R. Reers, alexandra.reers@seattlechildrens.org, Seattle Structural Genomics Center for Infectious Disease (SSGCID); Center for Global Infectious Disease Research, Seattle Children's Research Institute, 307 Westlake Avenue North, Suite 500, Seattle, ORCID: 0000-0003-2561-6648

Olivia Coburn-Flynn <ocoburnflynn@gmail.com>, Department of Microbiology & Immunology, Columbia University Medical Center, New York, NY 10032

Andrew S. Bell<a.bell11@imperial.ac.uk>, Molecular Sciences Research Hub, Imperial College, White City Campus Wood Lane, London W12 0BZ, UK, ORCID: 0000-0002-0581-9387

Judith Green <judith.green@crick.ac.uk>, Francis Crick Institute, 1 Midland Rd, London NW1 1AT, UK, ORCID: 0000-0001-6825-9404

Ellen Knuepfer <Ellen.Knuepfer@crick.ac.uk>, Francis Crick Institute, 1 Midland Rd, London NW1 1AT, UK, ORCID: 0000-0002-6090-1877

David Charter <davidjcharter@gmail.com>, Structural and Biophysical Sciences, GlaxoSmithKline, Stevenage, Hertfordshire, UK

Roger Bonnert, rogerbonnert@aol.com, Medicines for Malaria Venture, Route de Pré-Bois 20, Post Box 1826, CH-1215 Geneva 15, Switzerland, ORCID: 0000-0002-2976-7374

Brice Campo, campob@mmv.org, Medicines for Malaria Venture, Route de Pré-Bois 20, Post Box 1826, CH-1215 Geneva 15, Switzerland, ORCID: 0000-0002-6425-5577

Jeremy Burrows, burrowsj@mmv.org, Medicines for Malaria Venture, Route de Pré-Bois 20, Post Box 1826, CH-1215 Geneva 15, Switzerland, ORCID: 0000-0001-8448-6068

*Sally Lyons-Abbott, saox@novonordisk.com, Seattle Structural Genomics Center for Infectious Disease (SSGCID); Center for Global Infectious Disease Research, Seattle Children's Research Institute, 307 Westlake Avenue North, Suite 500, Seattle

Bart L. Staker, bart.staker@seattlechildrens.org, Seattle Structural Genomics Center for Infectious Disease (SSGCID); Center for Global Infectious Disease Research, Seattle Children's Research Institute, 307 Westlake Avenue North, Suite 500, Seattle, ORCID: 0000-0001-9570-5086

Chun-Wa Chung, <chun-wa.h.chung@gsk.com>, Structural and Biophysical Sciences, GlaxoSmithKline, Stevenage, Hertfordshire, UK; Crick–GSK Biomedical LinkLabs, GSK Medicines Research Centre, Stevenage, UK, ORCID: 0000-0002-2480-3110

Peter J. Myler, peter.myler@seattlechildrens.org, Seattle Structural Genomics Center for Infectious Disease (SSGCID); Center for Global Infectious Disease Research, Seattle Children's Research Institute, 307 Westlake Avenue North, Suite 500, Seattle, Department of Biomedical Informatics & Medical Education and Department of Global Health, University of Washington, ORCID: 0000-0002-0056-0513

David A. Fidock, <df2260@cumc.columbia.edu>, Department of Microbiology & Immunology, Columbia University Medical Center, New York, NY 10032 USA and Division of Infectious Diseases, Department of Medicine, Columbia University Medical Center, New York, NY 10032, ORCID: 0000-0001-6753-8938

**Edward W Tate, <e.tate@imperial.ac.uk> Molecular Sciences Research Hub, Imperial College, White City Campus Wood Lane, London W12 0BZ, UK, ORCID: 0000-0003-2213-5814

Anthony A. Holder, <Tony.Holder@crick.ac.uk>, 1 Midland Rd, London NW1 1AT, UK, ORCID: 0000-0002-8490-6058

*Current address: Novo Nordisk Research Center, Seattle, WA 98109, USA

**Lead Contact: Tate, Edward W., e.tate@imperial.ac.uk

**Corresponding Authors:** Tate, Edward W., e.tate@imperial.ac.uk; and Holder, Anthony A., Tony.Holder@crick.ac.uk , and Anja C Schlott anja.schlott@crick.ac.uk

**This PDF file includes:**

- Figure S1
- Figure S2
- Figure S3
- Figure S4
- Figure S5
- Figure S6
- Table S1
- Table S2
- Table S3
- Table S4
- Table S5
- Table S6

# Figures and legends SI Appendix

a)

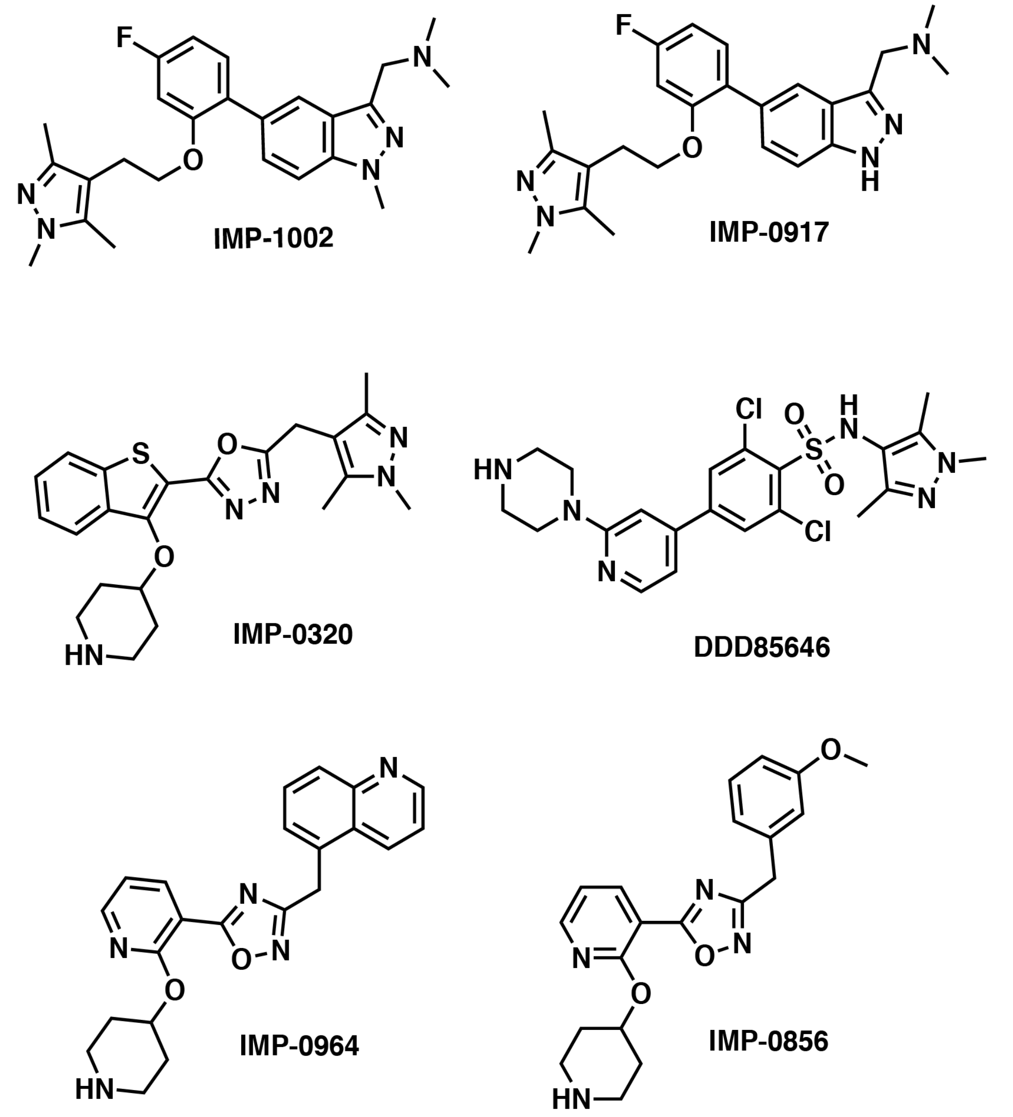


b)

| NMTi name | Series name | Publication | DOI of paper | PDB # WT | PDB # G386E |
|---|---|---|---|---|---|
| IMP-1002 | Series A | | | 6MB1 | 6MB0 |
| IMP-0917 | Series A | Mousnier et al., 2018 | 10.1038/s41557-018-0039-2 | 5O6H | – |
| DDD8646 | Series B | Wright et al. 2014 | 10.1038/nchem.1830 | 2YND | 6MAZ |
| IMP-0320 | Series C | Wright et al. 2014 | 10.1038/nchem.1830 | 2YNE | – |
| IMP-0964 | Series D | Yu et al., 2015 | 10.1039/c5md00242g | – | – |
| IMP-0856 | Series D | Yu et al., 2015 | 10.1039/c5md00242g | 4UFX | – |

**Figure S1.** a) Chemical structures of NMT inhibitors used in this study related to figure 1 and 6. b) Table of NMT inhibitors relating to their series name indicating closely related compounds, publication, and PDB # if a crystal structure is available.

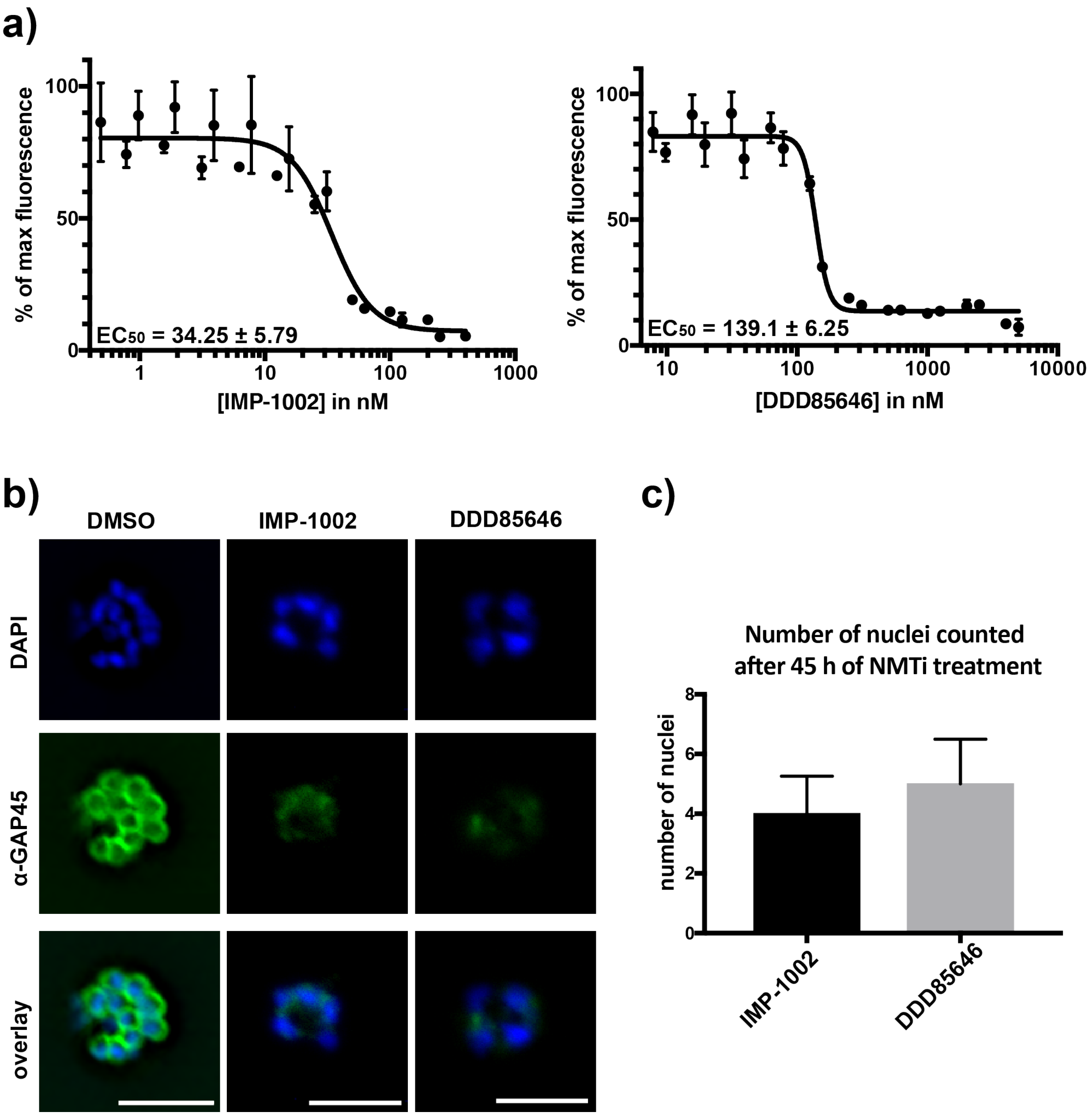


**Figure S2**. *P. falciparum* growth and development is inhibited by treatment with two NMT inhibitors related to figure 1 and 2.

a) *P. falciparum* growth, measured by fluorescence of SYBR Green bound to genomic DNA, in the presence of increasing concentrations of the NMT inhibitors. $EC_{50}$ values for compounds IMP-1002 and DDD85646 are indicated on the respective graphs. b) Indirect immunofluorescence assay of parasites incubated from the ring stage with a control (DMSO)

or four times the $EC_{50}$ concentration of each NMT inhibitor (1 µM DDD85646 and 140 nM IMP1002) and examined at 45 hours post invasion (h PI): nuclei stained by DAPI (blue) and GAP45 localization in green. GAP45 is a well-known NMT substrate and an indicator of the Inner membrane complex (IMC). The number of nuclei in each cell and the abundance and location of GAP45 were affected by drug treatment. All images were acquired with a Nikon PlanApo 100x/1.45 oil immersion objective. Scale bar = 5 µm. c) Number of nuclei counted at 45 h PI for parasites treated with IMP-1002 or DDD85646; error bars equal standard deviation (SD) of the median.

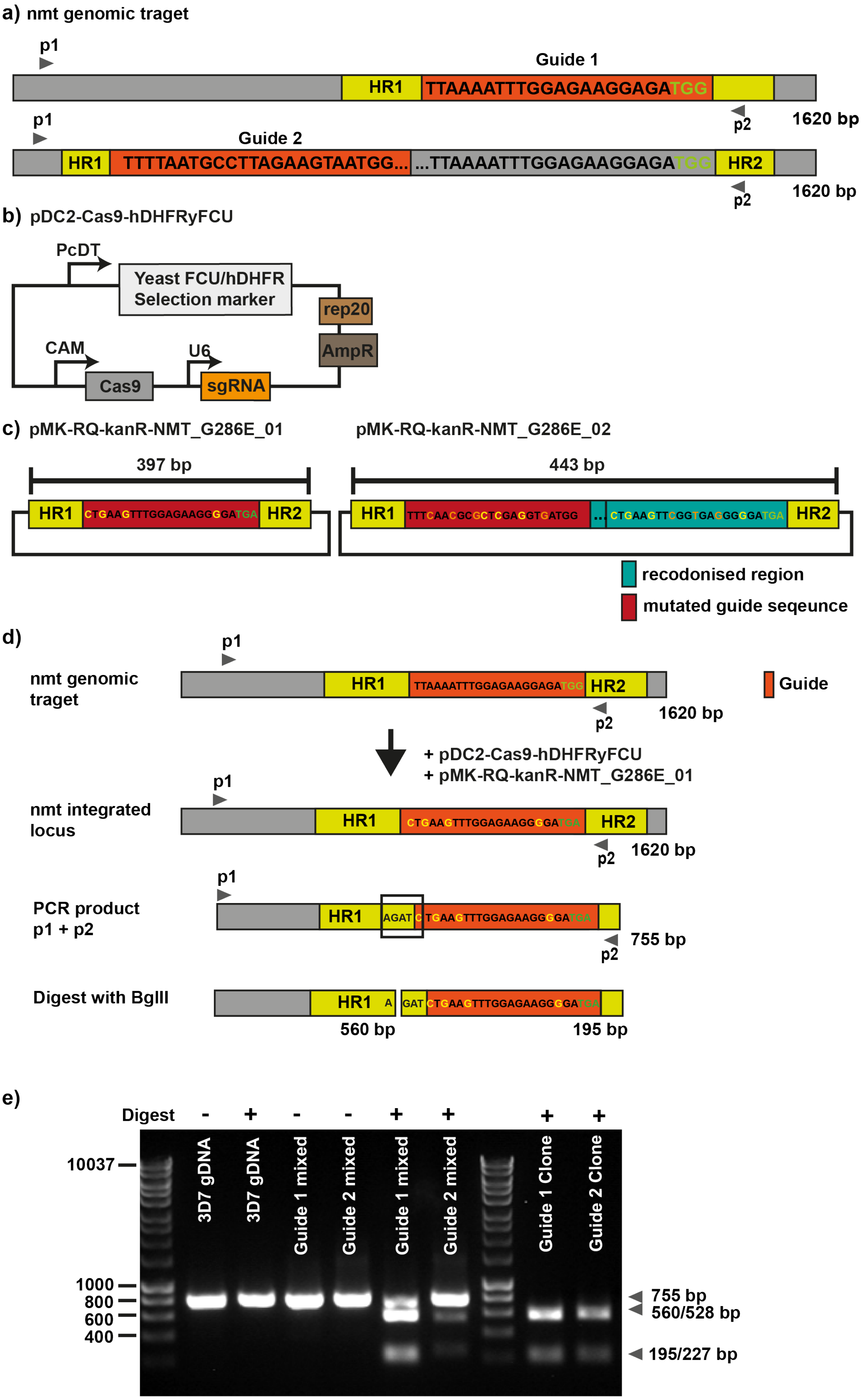
a) nmt genomic traget
p1
Guide 1
HR1
TTAAAATTTGGAGAAGGAGATGG
p2
1620 bp
p1
Guide 2
HR1
TTTTAATGCCTTAGAAGTAATGG...
...TTAAAATTTGGAGAAGGAGATGG
HR2
p2
1620 bp
b) pDC2-Cas9-hDHFRyFCU
PcDT
Yeast FCU/hDHFR Selection marker
rep20
AmpR
CAM
U6
Cas9
sgRNA
c) pMK-RQ-kanR-NMT_G286E_01
pMK-RQ-kanR-NMT_G286E_02
397 bp
443 bp
HR1
CTGAAGTTTGGAGAAGGGGATGA
HR2
HR1
TTTCAACGCGCTCGAGGTGATGG
...
CTGAAGTTCGGTGAGGGGGATGA
HR2
recodonised region
mutated guide seqeunce
d)
nmt genomic traget
p1
HR1
TTAAAATTTGGAGAAGGAGATGG
HR2
p2
1620 bp
Guide
+ pDC2-Cas9-hDHFRyFCU
+ pMK-RQ-kanR-NMT_G286E_01
nmt integrated locus
p1
HR1
CTGAAGTTTGGAGAAGGGGATGA
HR2
p2
1620 bp
PCR product p1 + p2
p1
HR1
AGATCTGAAGTTTGGAGAAGGGGATGA
p2
755 bp
Digest with BglII
HR1
A
GATCTGAAGTTTGGAGAAGGGGATGA
560 bp
195 bp
e)
Digest
-
+
-
-
+
+
+
+
10037
1000
800
600
400
3D7 gDNA
3D7 gDNA
Guide 1 mixed
Guide 2 mixed
Guide 1 mixed
Guide 2 mixed
Guide 1 Clone
Guide 2 Clone
755 bp
560/528 bp
195/227 bp

**Figure S3.** Generation of *P. falciparum* parasite lines containing the G386E mutation in the *nmt* gene related to figure 2 and 3.

a) Schematic of WT *nmt* ORF highlighting the two guide sequences and sequence before the desired mutation codon TGG highlighted in green. The PAM sequence of guide 1 was destroyed and mutated to yield the desired G386E mutation. For Guide 2 that lies upstream of the introduced mutation, a stretch of 45 bp was recodonised. b) Schematic of the Cas9/guide RNA plasmid. c) Schematic of the rescue plasmids with the modified open reading frame (OFR) containing the G386E point mutation (TGA) in green. Homology regions (HRs) on rescue plasmids used for targeting were 200 bp (HR1) and 169/174 bp (HR2) long. The two mutated guide sequences are shown in red. Silent point mutations for the generation of unique restriction sites in the integrated construct are highlighted in yellow. Additional silent mutations are highlighted in orange. d) Screening by PCR and restriction enzyme digest to distinguish WT from integrated parasites. Only integration of G286E_01 is shown. The restriction site is highlighted by a box. e) Agarose gel staining for DNA showing restriction digest of DNA amplified from parental line (3D7), mixed population for both transfections and the two clones used for the analysis from two independent transfections.

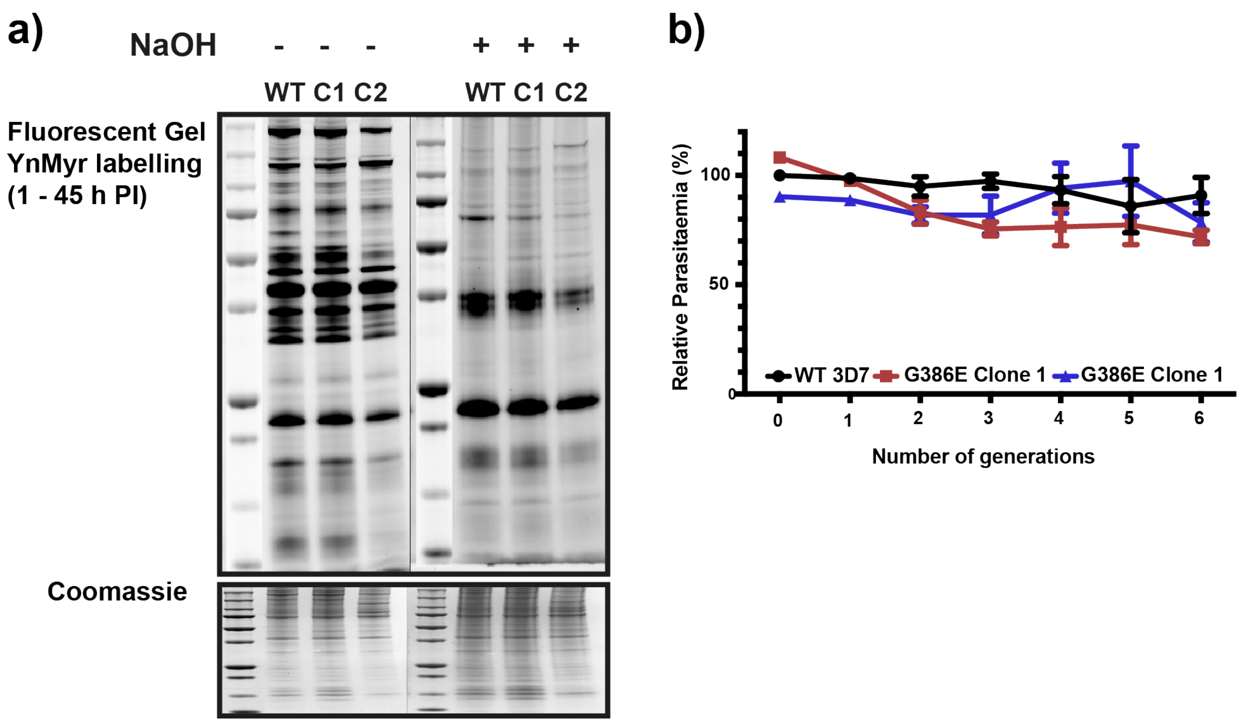


**Figure S4.** *P. falciparum* G386E parasites do not show a phenotype and show normal myristoylation patterns related to figure 3.

a) YnMyr labelling of *P. falciparum* 3D7 G386E and WT parasites during the entire erythrocyte life cycle. YnMyr labelling was subjected to NaOH treatment to hydrolyse the base-labile incorporation of YnMyr into GPI-anchored proteins. Coomassie stain indicates loading for each lane. b) Growth assay of *P. falciparum* 3D7 WT and G386E parasites over six generations. Starting parasitemia was determined by flow cytometry (Hoechst stain). WT parasitemia was determined after each cycle and a dilution factor calculated to give 0.8% parasitemia. All three lines were diluted using this same factor to avoid cultures overgrowing. Parasitemias were measured after each cycle using flow cytometry. Total parasitemia was normalized to the DMSO control replicate with the highest value. N = 3 biological replicates with 3 technical replicas each. Error bars equal SD. Multiple t-tests were performed for each generation (two-stage linear step-up procedure of Benjamini, Krieger and Yekutieli, with false-discovery

rate = 1 %). There was no significant difference in relative parasitemia between the three lines.

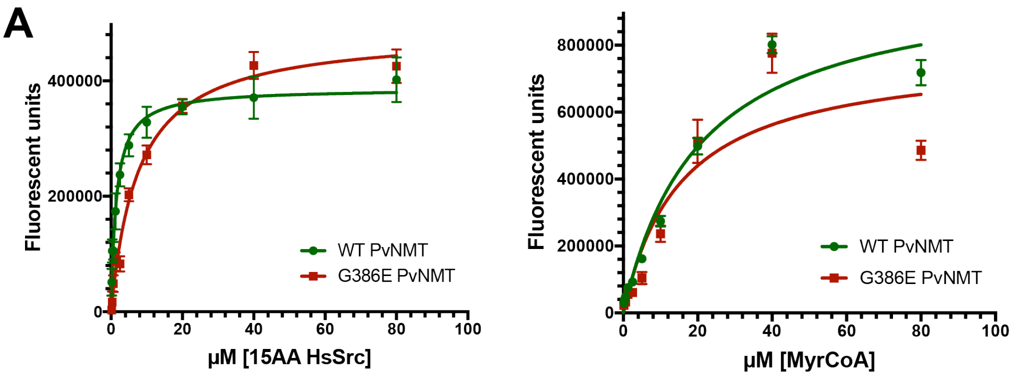


| | 15AA peptide substrate | | | | MyrCoA | |
|---|---|---|---|---|---|---|
| | PvNMT WT | | PvNMT[G386E] | | PvNMT WT | PvNMT[G386E] |
| Biological replicates | 1 | 2 | 1 | 2 | 1 | 1 |
| Km (µM) | 1.5 | 1.2 | 7.7 | 8.2 | 4.1 | 1.1 |
| Vmax (FU/sec) | 6446 | 5603 | 8096 | 8309 | 28568 | 25000 |
| Kcat | 19323 | 16795 | 24268 | 24907 | 90008 | 78769 |
| Vmax/Km | 4353 | 4661 | 1058 | 1008 | 6915 | 22666 |

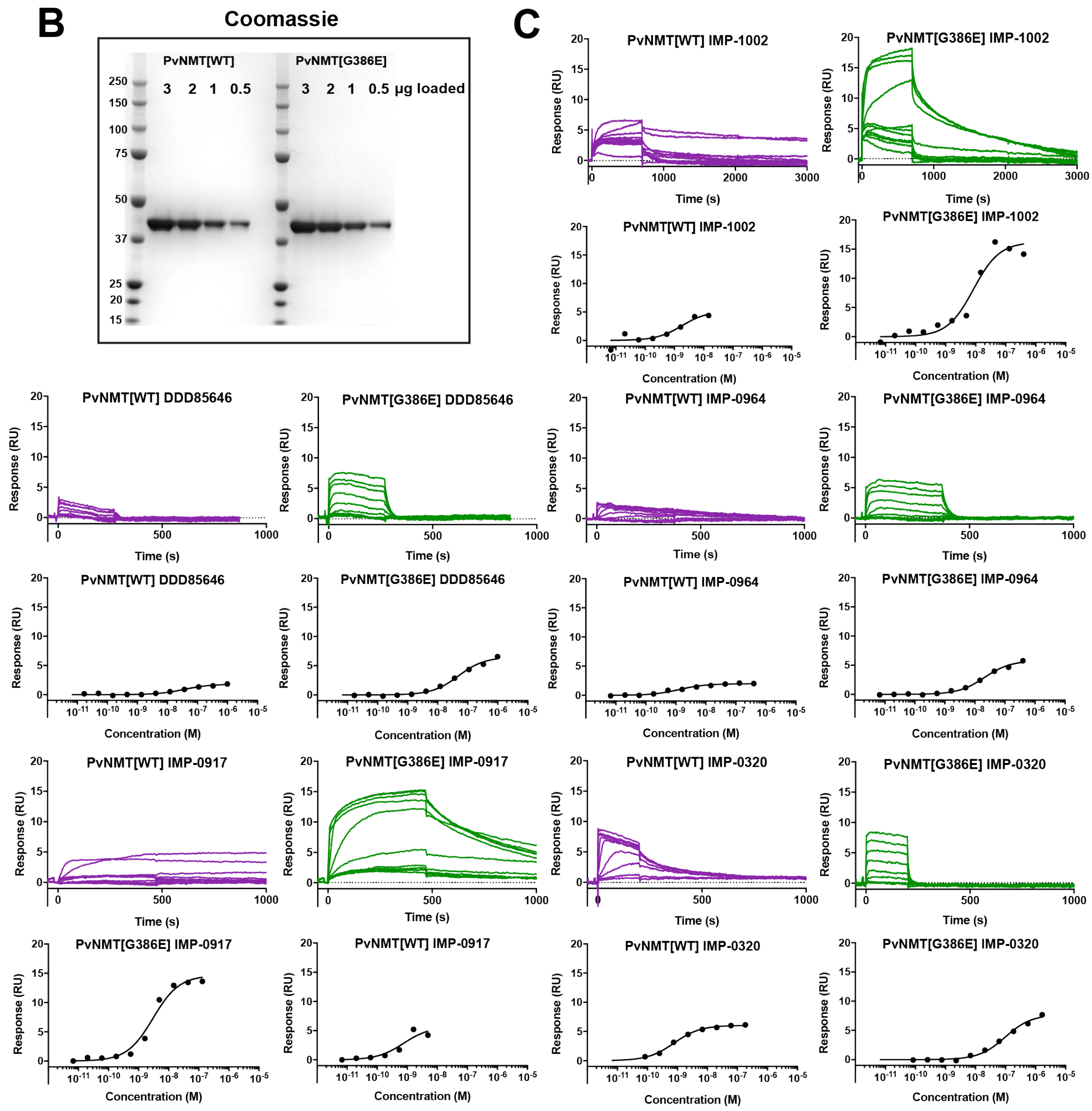

**Figure S5**. SPR equilibrium analysis of recombinant PvNMT[WT] and [G386E] variant related to figure 4 and 6.

A) Michaelis-Menten Plot for the KM Vmax determination for the myristoyl-CoA substrate and the 15-residue HsSrc peptide. PvNMT[WT] is in green and PvNMT[G386E] is in red. Table of KM, Vmax, Kcat and the catalytic efficiency (Vmax/KM) for the myrCoA and peptide kinetics. (n = 2 for peptide, n = 1 for MyrCoA). B) Coomassie of purified protein showing an indication of the purity of the sample. C) Surface plasmon resonance analysis of the interaction of NMTi with PvNMT[WT] and PvNMT[G386E]. The relationship between each point in the concentration series and the report points from sensorgrams measured for each of the NMTi compounds was fitted to a 1:1 steady state affinity model to calculate the equilibrium dissociation constant ($K_D$)

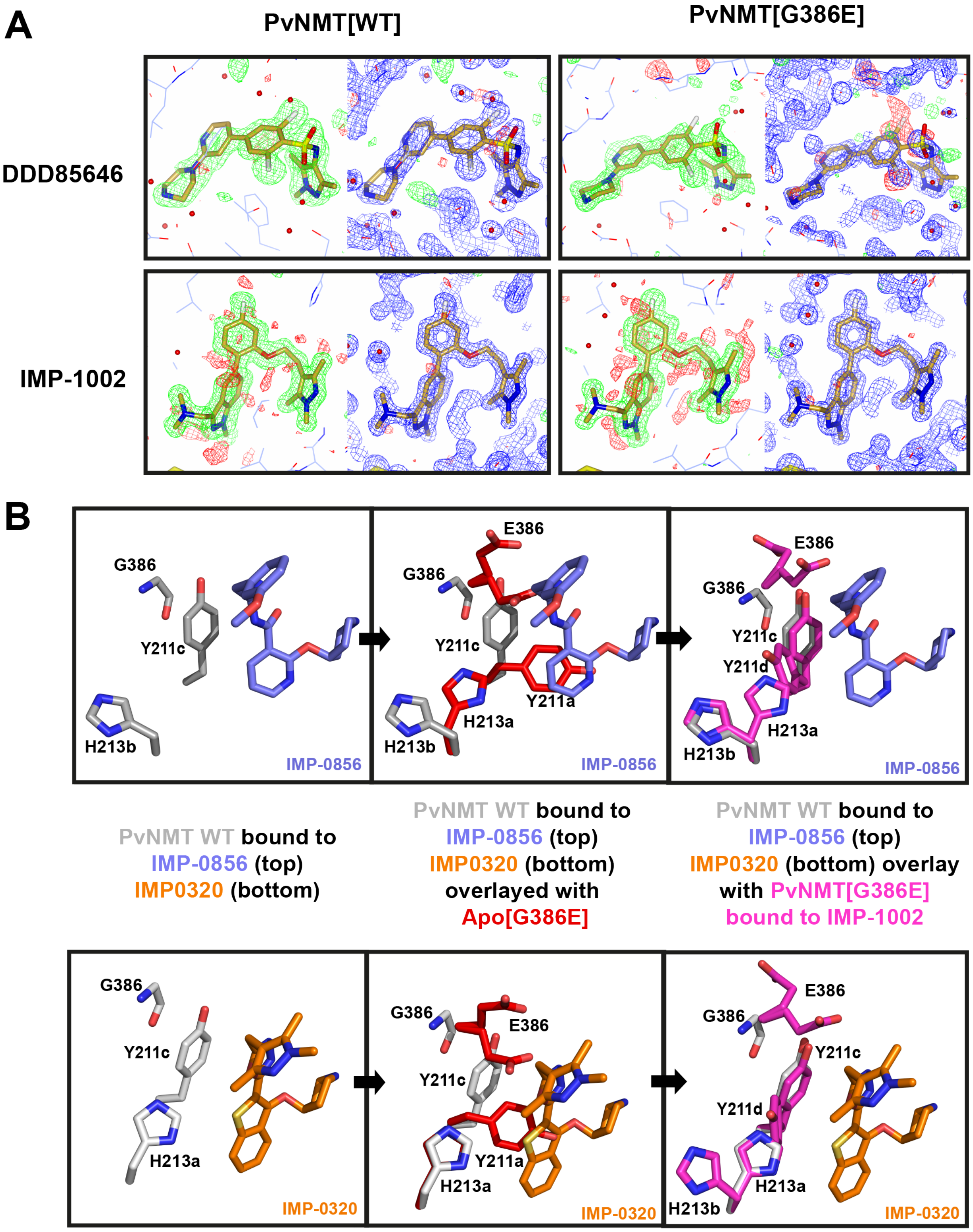


**Figure S6**: Crystallographic analysis of NMT inhibitor interaction with PvNMT[WT] and PvNMT[G386E] related to figure 5. A) Omit map for DDD85646 and IMP-1002 bound to PvNMT[WT] and PvNMT[G386E]. For each panel, FO-FC (contoured at 3.0 sigma) density for

fully refined structure with inhibitor atoms removed is shown on the left. For clarity, inhibitor stick representation is overlaid. For each panel, on the right is shown 2FO-FC (contoured at 1.0 sigma) and FO-FC (contoured at 3.0 sigma) density from fully refined structure including inhibitor atoms. B) Movement of residues as a result of the G386E mutation upon binding of two different NMT inhibitors: IMP-0856 at the top (PDB ID: 4UFX) and IMP-0320 (PDB ID: 2YNE) at the bottom. First image shows the PvNMT[WT] with respective inhibitor bound. Upon overlay with Apo[G386E] it becomes apparent that Y211 can no longer position in the Y211c rotamer because of the G386E substitution, and the Y211a rotamer cannot be adopted with IMP-0320 or IMP-0856 bound. Therefore, in the last image we predict that a PvNMT[G386E] bound to the respective NMTi would have similar characteristics as PvNMT[G386E] bound to IMP-1002 (PDB: 6MB0) with movement of Y211 from Y211c to Y211d.

| | G386E IMP1002 | WT IMP1002 | G386E IMP0366 | G386E (apo) |
|---|---|---|---|---|
| **Wavelength** | 0.97872 | 0.97872 | 0.97872 | 0.97872 |
| **Resolution range** | 50 - 1.55 (1.59 - 1.55) | 50 - 1.5 (1.54 - 1.50) | 50 - 1.55 (1.59 - 1.55) | 50 - 2.05 (2.10 - 2.05) |
| **Space group** | P 21 21 21 | P 21 21 21 | P 21 21 21 | P 21 21 21 |
| **Unit cell** | 57.44 121.19 178.25 90 90 90 | 57.56 119.11 176.08 90 90 90 | 57.5 121.25 177.93 90 90 90 | 57.81 120.81 179.06 90 90 90 |
| **Total reflections** | 1064299 (76554) | 1120216 (63971) | 1116596 (81282) | 491256 (36093) |
| **Unique reflections** | 176323 (12704) | 193711 (14201) | 180669 (13216) | 79557 (5777) |
| **Multiplicity** | 6.0 (6.0) | 5.8 (4.5) | 6.2 (6.2) | 6.2 (6.2) |
| **Completeness (%)** | 97.6 (96.2) | 99.9 (99.9) | 100.0 (100.0) | 99.9 (100.0) |
| **Mean I/sigma(I)** | 12.78 (3.18) | 11.91 (2.62) | 18.81 (3.40) | 15.01 (3.15) |
| **Wilson B-factor** | 11.32 | 12.23 | 12.57 | 23.55 |
| **R-merge** | 0.094 (0.544) | 0.090 (0.462) | 0.066 (0.537) | 0.096 (0.572) |
| **R-meas** | 0.102 (0.595) | 0.099 (0.523) | 0.073 (0.586) | 0.105 (0.624) |
| **CC1/2** | 0.997 (0.838) | 0.997 (0.822) | 0.999 (0.887) | 99.8 (0.875) |
| **Reflections used in refinement** | 176228 (17160) | 193683 (19198) | 180657 (17875) | 79549 (7843) |
| **Reflections used for R-free** | 1978 (196) | 2060 (195) | 2028 (207) | 2024 (183) |
| **R-work** | 0.1526 (0.2258) | 0.1549 (0.2129) | 0.1450 (0.1861) | 0.1571 (0.1907) |
| **R-free** | 0.1780 (0.2812) | 0.1839 (0.2363) | 0.1657 (0.2327) | 0.2178 (0.2542) |
| **Number of non-hydrogen atoms** | 11918 | 11841 | 11941 | 10882 |
| **macromolecules** | 9752 | 9687 | 9707 | 9462 |
| **ligands** | 314 | 325 | 314 | 230 |
| **solvent** | 1852 | 1829 | 1920 | 1190 |
| **Protein residues** | 1144 | 1144 | 1143 | 1136 |
| **RMS(bonds)** | 0.006 | 0.005 | 0.006 | 0.007 |
| **RMS(angles)** | 0.82 | 0.79 | 0.84 | 0.88 |
| **Ramachandran favored (%)** | 97.8 | 97.71 | 97.71 | 97.07 |
| **Ramachandran allowed (%)** | 2.2 | 2.29 | 2.29 | 2.84 |
| **Ramachandran outliers (%)** | 0 | 0 | 0 | 0.09 |
| **Rotamer outliers (%)** | 0.55 | 0.55 | 0.28 | 0.58 |
| **Clashscore** | 1.55 | 0.9 | 2.56 | 1.5 |
| **Average B-factor** | 16.06 | 17.1 | 17.82 | 29.01 |
| **macromolecules** | 13.85 | 14.98 | 15.32 | 27.7 |
| **ligands** | 11.87 | 14.28 | 19.58 | 48.67 |
| **solvent** | 28.38 | 28.85 | 30.16 | 35.6 |
| **Number of TLS groups** | 16 | 17 | 22 | 9 |

**Table S1**: Data collection and refinement statistics of crystallographic data related to figure 5.

**Growth assay ($EC_{50}$ determination)**

| | PfNMT[WT] | | | PfNMT[G398E] Clone 1 | | | PfNMT[G398E] Clone 2 | | | Ratio PfNMT[G386] / PfNMT[WT] | |
|---|---|---|---|---|---|---|---|---|---|---|---|
| | $EC_{50}$ (nM) | 95% CI (nM) | Hill Slope | $EC_{50}$ (nM) | 95% CI (nM) | Hill Slope | $EC_{50}$ (nM) | 95% CI (nM) | Hill Slope | Clone 1 | Clone 2 |
| IMP-1002 | 9.6 | 8.2 - 11 | -2.3 | 230 | 190 - 280 | -1.7 | 201 | 170 - 250 | -1.5 | 24 | 21 |
| DDD85646 | 69 | 51 - 93 | -0.65 | 91 | 75 - 110 | -1.4 | 103 | 86 - 120 | -1.5 | 1.3 | 1.5 |
| IMP-0964 | 93 | 83 - 103 | -2.9 | 1080 | 913 - 1300 | -1.3 | 947 | 78 - 83 | -0.89 | 12 | 10 |
| IMP-0320 | 80 | 68 - 93 | -2.6 | 1500 | 1200 - 1800 | -1.4 | 1400 | 1100 - 1900 | -0.91 | 19 | 18 |

**Table S2**. Raw data for SYBR Green growth assay related to figure 4 and 6. SYBR Green growth assay data to determine the $EC_{50}$ of [WT] and [G386E] parasites with two biological replicates in the form of two clones from independent transfections with different guides. All numbers are given to two significant figures. In the last column the ratio is calculated of the $EC_{50}$ of [G386E]/[WT].

**CPM enzyme assay ($IC_{50}$ determination)**

| | | PvNMT[WT] | | | PvNMT[G398E] | | | |
|---|---|---|---|---|---|---|---|---|
| | **Replicate** | **$IC_{50}$ (nM)** | **95% CI (nM)** | **Hill Slope** | **$IC_{50}$ (nM)** | **95% CI (nM)** | **Hill Slope** | **Ratio PvNMT[G386E]/ PvNMT[WT]** |
| **IMP-1002** | **1** | 1.9 | 1.6 - 2.1 | -2.1 | 6.0 | 5.4 - 6.5 | -1.8 | 3.1 |
| | **2** | 2.1 | 2.0 - 2.3 | -1.7 | 7.1 | 2.3 - 41 | -0.82 | 3.4 |
| | **3** | 4.9 | 4.4 - 5.4 | -2.0 | 6.9 | 6.2 - 7.7 | -1.5 | 1.4 |
| **DDD85646** | **1** | 53 | 38 - 65 | -0.9 | 55 | 41 - 62 | -0.9 | 1.0 |
| | **2** | 47 | 37 - 66 | -1.1 | 77 | 61 - 110 | -1.5 | 1.6 |
| | **3** | 48 | 40 - 62 | -1.1 | 80 | 52 - 204 | -0.9 | 1.7 |
| **IMP-0964** | **1** | 3.7 | 3.3 - 4.2 | -1.2 | 21 | 18 - 24 | -1.2 | 5.5 |
| | **2** | 4.7 | 2.3 - 7.2 | -3.6 | 17 | 12 - 24 | -1.3 | 3.6 |
| | **3** | 12 | 12 - 13 | -1.9 | 35 | 33 - 38 | -1.5 | 2.9 |
| **IMP-0320** | **1** | 10 | 9.2 - 11 | -1.2 | 380 | 180 - 4900 | -0.9 | 38 |
| | **2** | 9.1 | 8.5 - 10 | -1.3 | 340 | 140 - 1800 | -0.9 | 37 |
| | **3** | 10 | 9.4 - 11 | -1.3 | 300 | 150 - 6800 | -1.0 | 29 |

**Table S3**. Raw data for CPM enzyme assay related to figure 4 and 6. CPM enzyme assay data to determine the $IC_{50}$ of PvNMT[WT] and PvNMT[G386E] with three biological replicates of four different NMTi compounds. All numbers are given to two significant figures. The Hill Slope of the $IC_{50}$ gives an indication how steep the curve is. For very high hill slopes < -1, the $IC_{50}$ value cannot be accurately determine and is presumably lower than indicated. In the last column the ratio is calculated of the $IC_{50}$ of PvNMT[G386E]/PvNMT[WT].

**a)**

| All Tm in °C | Tm | Tm | ΔTm | ΔTm | ΔΔTm |
|---|---|---|---|---|---|
| **Protein** | | | | | PvNMT[WT] - |
| **- MyrCoA** | PvNMT[WT] | PvNMT[G386E] | PvNMT[WT] | PvNMT[G386E] | PvNMT[G386E] |
| **Compound ↓** | | | | | |
| 1% DMSO reference | 64.6 ± 0.6 | 61.0 ± 0.7 | 0 | 0 | 0 |
| IMP-0320 | >70.4 # | 61.7 ± 0.8 | >5.7 | 0.7 | >5.0 |
| DDD85646 | 69.0 ± 0.1 | 62.4 ± 0.7 | 4.4 | 1.3 | 3 |
| IMP-1002 | >73.4 # | 62.8 ± 0.7 | >8.8 | 1.8 | >7.0 |

# Protein was not fully denatured at the end of the thermal ramp, therefore these values represent the lower estimates of Tm

**b)**

| All Tm in °C | Tm | Tm | ΔTm | ΔTm | ΔΔTm |
|---|---|---|---|---|---|
| **Protein** | | | | | PvNMT[WT] - |
| **+ 4μM MyrCoA** | PvNMT[WT] | PvNMT[G386E] | PvNMT[WT] | PvNMT[G386E] | PvNMT[G386E] |
| **Compound ↓** | | | | | |
| 1% DMSO reference | 67.8 ± 0.6 | 61.6 ± 0.5 | 0 | 0 | 0 |
| IMP-0320 | >73 # | 63.2 ± 2.2 | >5.2 | 1.6 | >3.6 |
| DDD85646 | 71.0 ± 0.1 | 63.0 ± 0.3 | 3.2 | 1.4 | 1.8 |
| IMP-1002 | >75 # | 66.6 ± 0.6 | >7.2 | 5 | >2.2 |

# Protein was not fully denatured at the end of the thermal ramp, therefore these values represent the lower estimates of Tm.

**Table S4:** Thermal shift assay data related to figure 4. The degree of stabilisation of PvNMT[WT] and PvNMT[G386E] was determined using at least two experimental replicates with two technical replicates (n>5) with three different NMTi compounds in the absence (a) and (b) presence of (myrCoA). All Tm values are given in °C and ΔTm is determined by subtracting the Tm for the DMSO control to determine the compound induced stabilisation each protein. There is significantly greater stabilisation of the PvNMT [WT] for compounds such as IMP-0320 and IMP-1002 over PvNMT [G386E], as evident from the ΔΔTm values. For the PvNMT [WT], where the Tm of the compound bound protein exceeds the top of the temperature range for the instrument, a greater than value for the Tm is given. These lower estimates of Tm may lead to compression of the difference window between PvNMT [WT] and PvNMT [G386E] for IMP-0320 and IMP-1002. This is most noticeable with the higher baseline Tm of PvNMT [WT] in the presence of myrCoA.

| | HsNMT1 | HsNMT2 | PvNMT | HsNMT1/PvNMT | HsNMT2/PvNMT |
|---|---|---|---|---|---|
| **IMP-1002** | < 0.0072 ×/÷ 3.7821 (n=4) | 0.0119 ×/÷ 2.3488 (n=3) | < 0.0031 ×/÷ 2.5220 (n=7) | 2.32 | 3.84 |
| **DDD85646** | 0.0127 ×/÷ 1.4054 (n=3) | 0.0200 (n=1) | 0.0407 ×/÷ 1.6927 (n=5) | 0.3 | 0.5 |

**Table S5**. $IC_{50}$ of HsNMT1 HsNMT2 and PvNMT with IMP-1002 and DDD85646 determined through CPM assay related to figure 1. Values are concentrations in µM. Values are the geometric mean with associated standard error showing the variation from the biological replicates. The last two columns highlight the fold difference in $IC_{50}$ of PvNMT compared to HsNMT1 and HsNMT2. While there is a 2-3 fold shift in selectivity towards PvNMT with IMP-1002 this selectivity is diminished for DDD85646.

**a)**

| Compound | PvNMT [WT] KD (expt1) | PvNMT [WT] (expt2) | PvNMT [WT] (expt3) | PvNMT [WT] (expt4) |
|---|---|---|---|---|
| **IMP-0320** | 7.79E-10 | 2.41E-09 | 7.47E-09 | 6.87E-10 |
| **DDD85646** | 3.61E-08 | 2.96E-08 | 1.51E-08 | 2.18E-08 |
| **IMP-0964** | 1.29E-09 | 6.47E-10 | 8.17E-09 | 3.66E-10 |
| **IMP-1002** | 1.80E-09 | | | |
| **IMP-0917** | 6.48E-10 | 7.52E-10 | | |

**b)**

| Compound | PvNMT[G386E] KD (expt1) | PvNMT[G386E] (expt2) | PvNMT[G386E] (expt3) | PvNMT[G386E] (expt4) |
|---|---|---|---|---|
| **IMP-0320** | 9.72E-08 | 1.88E-07 | 1.68E-07 | 9.95E-08 |
| **DDD85646** | 4.90E-08 | 4.47E-08 | 2.23E-08 | 4.91E-08 |
| **IMP-0964** | 2.07E-08 | 1.64E-08 | 1.52E-08 | 1.22E-08 |
| **IMP-1002** | 8.37E-09 | 4.83E-09 | 3.63E-09 | 2.98E-09 |
| **IMP-0917** | 2.58E-09 | 3.29E-09 | | |

**c)**

| Compound | PvNMT[G386E]/ PvNMT[WT] KD (expt1) | PvNMT[G386E]/ PvNMT[WT] (expt2) | PvNMT[G386E]/ PvNMT[WT] (expt3) | PvNMT[G386E]/ PvNMT[WT] (expt4) |
|---|---|---|---|---|
| **IMP-0320** | 77.8 | 22.5 | 124.8 | 144.9 |
| **DDD85646** | 1.5 | 1.5 | 2.3 | 1.4 |
| **IMP-0964** | 25.3 | 41.5 | 16.1 | 11.7 |
| **IMP-1002** | 4.7 | | | |
| **IMP-0917** | 4 | 4.4 | | |

**d)**

| **Compound** | **$K_D$ (PvNMT[G86E]) / $K_D$ PvNMT[WT])** |
|---|---|
| IMP-0320 | 92.5 ±54.5 |
| DDD85646 | 1.6 ±0.4 |
| IMP-0964 | 19.9 ±14.2 |
| IMP-1002 | 4.7 (n =1) |
| IMP-0917 | 4.2 ±0.3 |

**Table S6**. Fold difference of equilibrium dissociation constants (KD) determined from SPR analyses for the interaction between each NMTi compound with PvNMT[WT] or PvNMT[G386E] related to figure 4 and 6. a) Raw data of $K_D$ values for PvNMT[WT] (n = 1-4) and b) PvNMT[G386E] (n = 2-4). c) The SPR experiment was configured so that the same concentration titrations were flowed over surfaces immobilized with the three proteins. To focus on the selectivity between proteins and remove inter experiment variability, the ratio of KD's between the three proteins was calculated in each experiment (n = 1-4) as this allows better differentiate between differences in affinity. d) The $K_D$ of each compound with either

PvNMT[G386E] was divided by the $K_D$ with PvNMT[WT] to calculate fold difference. The standard deviation of the mean fold difference is stated.
